# Rapid *in vitro* generation of bona fide exhausted CD8+ T cells is accompanied by *Tcf7* promotor methylation

**DOI:** 10.1371/journal.ppat.1008555

**Published:** 2020-06-24

**Authors:** Manzhi Zhao, Caoimhe H. Kiernan, Christopher J. Stairiker, Jennifer L. Hope, Leticia G. Leon, Marjan van Meurs, Inge Brouwers-Haspels, Ruben Boers, Joachim Boers, Joost Gribnau, Wilfred F. J. van IJcken, Eric M. Bindels, Remco M. Hoogenboezem, Stefan J. Erkeland, Yvonne M. Mueller, Peter D. Katsikis

**Affiliations:** 1 Department of Immunology, Erasmus University Medical Center, Rotterdam, The Netherlands; 2 Cancer Immunology and Tumor Microenvironment Program, Sanford Burnham Prebys Medical Discovery Institute, La Jolla, CA, United States of America; 3 Department of Developmental Biology, Erasmus University Medical Center, Rotterdam, The Netherlands; 4 Oncode Institute, Erasmus University Medical Center, Rotterdam, The Netherlands; 5 Center for Biomics, Erasmus University Medical Center, Rotterdam, The Netherlands; 6 Department of Hematology, Erasmus University Medical Center, Rotterdam, The Netherlands; ETH Zurich, SWITZERLAND

## Abstract

Exhaustion is a dysfunctional state of cytotoxic CD8+ T cells (CTL) observed in chronic infection and cancer. Current *in vivo* models of CTL exhaustion using chronic viral infections or cancer yield very few exhausted CTL, limiting the analysis that can be done on these cells. Establishing an *in vitro* system that rapidly induces CTL exhaustion would therefore greatly facilitate the study of this phenotype, identify the truly exhaustion-associated changes and allow the testing of novel approaches to reverse or prevent exhaustion. Here we show that repeat stimulation of purified TCR transgenic OT-I CTL with their specific peptide induces all the functional (reduced cytokine production and polyfunctionality, decreased *in vivo* expansion capacity) and phenotypic (increased inhibitory receptors expression and transcription factor changes) characteristics of exhaustion. Importantly, *in vitro* exhausted cells shared the transcriptomic characteristics of the gold standard of exhaustion, CTL from LCMV cl13 infections. Gene expression of both *in vitro* and *in vivo* exhausted CTL was distinct from T cell anergy. Using this system, we show that *Tcf7* promoter DNA methylation contributes to TCF1 downregulation in exhausted CTL. Thus this novel *in vitro* system can be used to identify genes and signaling pathways involved in exhaustion and will facilitate the screening of reagents that prevent/reverse CTL exhaustion.

## Author summary

In this manuscript, we describe an *in vitro* method that rapidly establishes large numbers of exhausted CD8+ T cells. The exhaustion of CTL induced by this method has been fully validated by multiple approaches (cytokine production, polyfunctionality, cytotoxicity, *in vivo* proliferation, inhibitory receptors, transcription factors, RNAseq and DNA methylation). This method will facilitate not only the study of T cell exhaustion but also the screening of drugs. As proof of point, we use this method to show that TCF-1 downregulation in terminally exhausted T cells is accompanied by *Tcf7* DNA promoter methylation and show that a transmethylase inhibitor can prevent TCF-1 downregulation. Our method presents a critical resource for the study of CTL exhaustion and the screening of drugs and interventions.

## Introduction

Cytotoxic CD8+ T cells (CTL) play a critical role in eliminating viral infection and controlling cancer development. During chronic viral infection and cancer, CTL acquire a state of dysfunction that is often referred as CTL exhaustion which was originally described in chronic Lymphocytic Choriomeningitis Virus (LCMV) infection of mice [1]. CTL exhaustion has been documented in humans in chronic viral infections such as human immunodeficiency virus (HIV), hepatitis B virus (HBV) and hepatitis C virus (HCV) infections and in most human cancers [2–9] and is thought to be a central mechanism behind the failure of CTL to eliminate chronically infected and cancerous cells. Preventing and/or reverting exhaustion therefore constitutes a promising approach to restore the function of these CD8+ T cells. This requires however an in depth understanding of the mechanisms that lead to exhaustion and the stimuli that affect exhausted CD8+ T cells.

In recent years, the characteristics of exhausted CTL have been intensively researched by comparing antigen-specific CTL in chronic viral infection or cancer with effector and memory cells in acute virus infection [10–12]. Exhaustion is characterized by loss of cytokine production, such as interleukin-2 (IL-2), tumor necrosis factor-α (TNF-α) and interferon-γ (IFN-γ), decreased cytokine polyfunctionality, diminished expansion potential [13] and sustained high expression of multiple inhibitory receptors such as PD-1, Tim3, Lag3, TIGIT, CD160 and CD244 [14–17].

The phenotypic and functional changes of exhausted CTL arise from an altered transcriptional profile and modified epigenetic landscape [12, 18–20]. Altered expression of transcription factors and repressors such as T cell factor-1 (TCF) [21, 22], Thymocyte selection-associated HMG box protein (TOX) [23–27], T-box transcription factor 21 (T-bet) [12], Eomesodermin (EOMES) [1], IRF4 [28], NR4a [29] and BAFT [30] are indicative for the exhaustion phenotype. For example, transcription factor T-bet and TCF1 commonly expressed by functional effector and memory CTL, are also expressed by exhausted CTL, but are associated with distinct gene expression [12, 31, 32]. TOX expression has been associated with molecular and epigenetic programs of CTL exhaustion [23, 24, 26, 27]. In addition, in comparison to effector and memory CTL, exhausted CTL also have a distinct epigenetic landscape that contributes to their phenotypic changes and gene expression [19, 20].

To study T cell exhaustion, *in vivo* mouse models are commonly used where T cell exhaustion is either induced through chronic viral infection or cancer. LCMV infection is a well-characterized mouse model for CD8+ T cell exhaustion [10, 33]. The exhausted phenotype is also detected among tumor infiltrating lymphocytes (TIL) in tumor mouse models [34, 35]. The persistence of the antigen has been shown to be the crucial element [36] and chronic antigen stimulation alone is sufficient to induce CTL exhaustion *in vivo* [37, 38]. Previous *in vitro* exhaustion protocols have failed to validate T cell exhaustion, assuming inhibitory receptor expression and reduction of IL-2 production as surrogates for dysfunction [39]. Some of these changes, however, such as expression of PD-1 and CD160 also accompanies T cell activation while loss of IL-2 production is a feature of effector differentiation. Thus a fully validated *in vitro* exhaustion system has yet to be established.

The importance of CTL exhaustion in chronic infections and cancer demands rapid *in vitro* methods to screen new approaches that can prevent or revert CTL exhaustion. Inducing CTL exhaustion *in vivo* is time-consuming, requiring more than 30 days and yields limited numbers of exhausted cells as experimental material. Most importantly the *in vivo* milieu in these models is characterized by inflammation, high viral loads, suppressive cytokines or cells; all of which can obscure the phenotype of exhausted CTL and therefore make it hard to dissect true exhaustion-associated changes. Our newly developed *in vitro* method circumvents all of the above mentioned issues and allows gene manipulation and screening of small molecules or antibodies that could restore the function of exhausted CTL and/or prevent the induction of exhaustion. Using this system, we can show that the *Tcf7* promotor is methylated in exhausted CTL and using DNA transmethylase inhibitors one can prevent the downregulation of TCF1. Therefore, this 5-day *in vitro* exhaustion system enables the rapid testing of new approaches to prevent and/or revert exhaustion in T cells and therefore may facilitate the development of new therapies for chronic infections and cancer.

## Results

### Repeat stimulation with cognate peptide to induce CTL exhaustion

**Fig 1:**
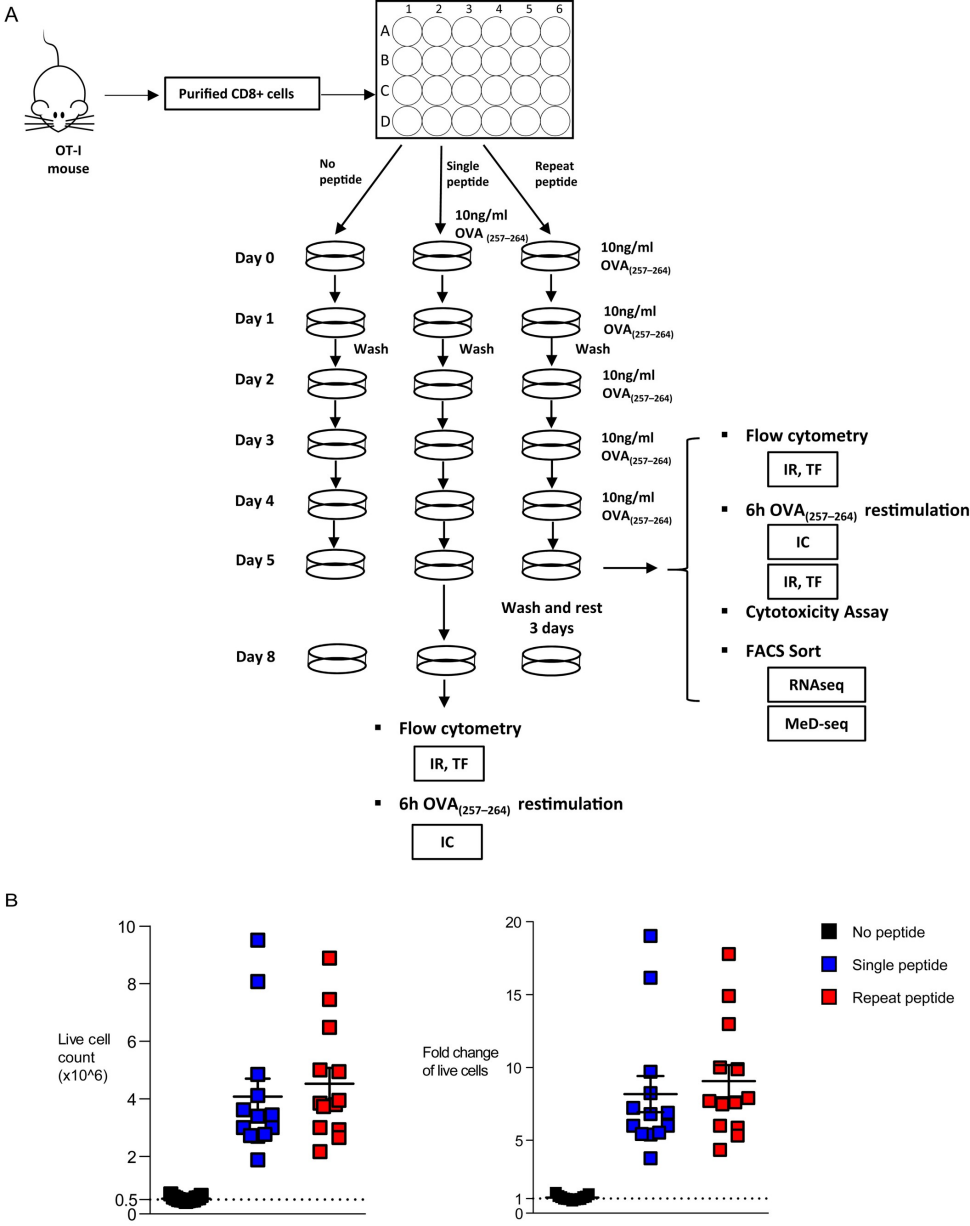
Scheme of the experimental protocol and numbers of live exhausted CTL generated by *in vitro* exhaustion. (A) Scheme of the experimental set up. IR: Inhibitory receptors, TF: Transcription factors, IC: Intracellular (B) Purified CD8+ T cells (0.5x10^6^ cells per well) were cultured either unstimulated, stimulated once with peptide and repeat peptide stimulated. Live cells were counted on day 5. Pooled data showing absolute cell numbers (left) and fold expansion (right). Data are from n = 13 and 10 independent experiments.

Because of the critical role sustained antigen stimulation plays in driving CD8+ T cells to exhaustion [36], we utilized repeat peptide stimulations of OVA_(257–264)_−specific TCR transgenic OT-I cells to induce CTL exhaustion *in vitro*. To induce exhaustion, CD8+ T cells purified from OT-I mice were stimulated daily for five days with 10ng/ml OVA_(257–264)_ peptide in the presence of IL-15 and IL-7 (Repeat peptide stimulation, Fig 1A). As controls, cells were either left unstimulated or stimulated only once with OVA_(257–264)_ peptide for 48 hours and then washed and cultured without peptide for an additional 3 days. All cells were cultured with IL-7 and IL-15. On day 5, cells were either harvested for analysis or in some instances cells were cultured an additional 3 days in the presence of IL-7 and IL-15 without any additional peptide stimulation (Fig 1A).

An important feature of this *in vitro* exhaustion system is that it rapidly yields large numbers of exhausted cells within 5 days (Fig 1B). Single peptide stimulations yielded comparable numbers while unstimulated culture numbers remained largely unchanged. The expansion in the numbers of exhausted T cells represents a ~9-fold increase from the T cell numbers seeded. Thus this *in vitro* exhaustion system can yield large numbers of exhausted CTL that can be used to further study and manipulate the pathways that drive exhaustion.

### Repeat peptide stimulation reduces cytokine production and leads to loss of polyfunctionality

**Fig 2:**
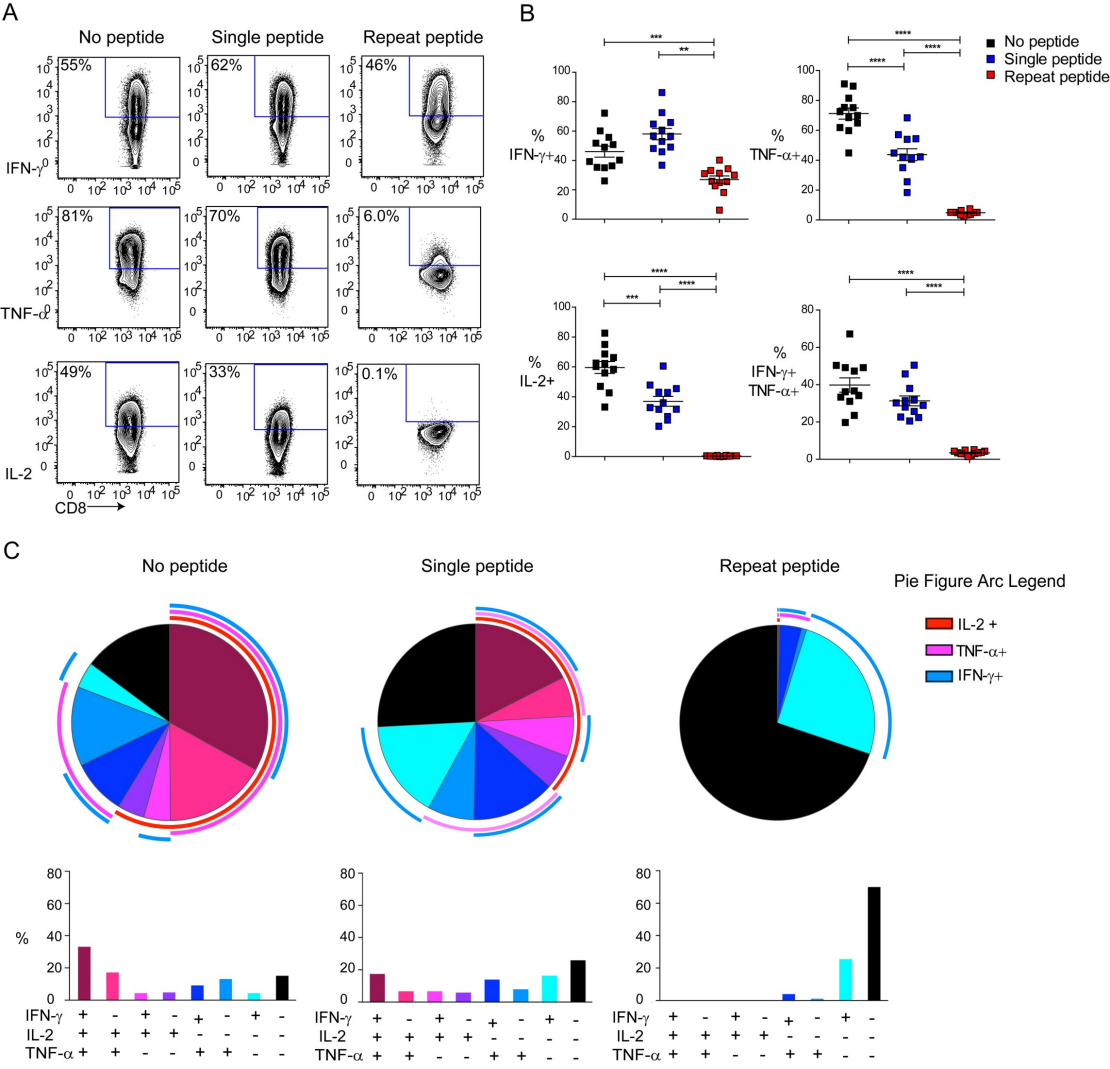
Repeat peptide stimulated CD8+ T cells show reduced cytokine production capacity and lose polyfunctionality. Purified OT-I CD8+ T cells were cultured either without peptide (no peptide), stimulated one time for 2 days with OVA peptide (single peptide) or stimulated with OVA peptide daily (repeat peptide). On day 5 cells were harvested and re-stimulated with OVA peptide. (A) Representative flow cytometry plots showing frequency of cytokine producing live CD8+ T cells after re-stimulation. (B) Pooled data showing the frequency of cytokine producing cells after re-stimulating with OVA peptide. (C) SPICE figures depicting the frequency of cells producing one, two or three cytokines in different combinations. Each symbol represents one animal (n = 11–12), 9 independent experiments performed. Line depicts mean ± SE. Between the groups, Student’s t test with Welch’s correction was performed except for % IFN-γ+ (ANOVA with Tukey’s post hoc test). *<0.05, **P<0.01, ***P<0.001, ****P<0.0001.

Since hierarchical loss in the capacity to produce cytokines is one of the most critical characteristics of exhausted CD8+ T cells [10], we first examined the capacity for cytokine release upon peptide re-stimulation. After five days of culture, the cells were harvested and stimulated with OVA_(257–264)_ peptide in the presence of Golgi plug for 6 hours. More than 40 percent of the unstimulated cells could produce IFN-γ upon re-stimulation (46±4%, mean ± standard error, Fig 1A and 1B). Single peptide stimulated cells were also able to produce IFN-γ (58±4%). In contrast, repeat peptide stimulated cells had impaired IFN-γ production with only 27±3% of cells able to produce IFN-γ after re-stimulation (Fig 2A and 2B). In addition, very few repeat peptide stimulated cells (4.8±0.4%) could produce TNF-α compared to single peptide stimulated cells (44±4%) and unstimulated cells (71±4%) (Fig 2A and 2B). IL-2-producing cells could barely be detected in the repeat peptide stimulated cells after re-stimulation, while IL-2 was readily produced by single peptide stimulated cells (37±3%) and unstimulated cells (60±4%) (Fig 2A and 2B). Ultimately, these results demonstrate that repeat peptide stimulation of CD8+ T cells *in vitro* leads to impaired cytokine production in a manner that has been previously reported in exhausted CD8+ T cells, first IL-2 production is lost followed by TNF-α and finally IFN-γ.

Exhausted CD8+ T cells, unlike memory cells, cannot produce multiple cytokines at the same time, and we therefore assessed the polyfunctionality of our *in vitro* generated cells. For this, the percentage of single, double and triple cytokine producing cells were determined and visualized using the SPICE software. In unstimulated cultured cells, 33% produce all three cytokines simultaneously upon peptide stimulation while significant percentages of these cells were double producer (Fig 2C). Only 15% of the cells did not produce any of these three cytokines. Similarly, in the single peptide stimulated cells, 18% were triple positive for all three cytokines, large numbers were double cytokine producers, while 26% were incapable of releasing any of these cytokines (Fig 2C). In contrast, 70% of the repeat peptide stimulated cells could not produce any cytokines after re-stimulation. IFN-γ single producers were 25% of the cells, while very few cells produced other cytokines (Fig 2C). To demonstrate the persistence of the cytokine profile of repeat peptide stimulated cells, we rested cells without peptide for another 3 days before examining their cytokine production. We found that the reduced cytokine production of repeat peptide stimulated cells was maintained after resting (S1A Fig).

**Fig 3:**
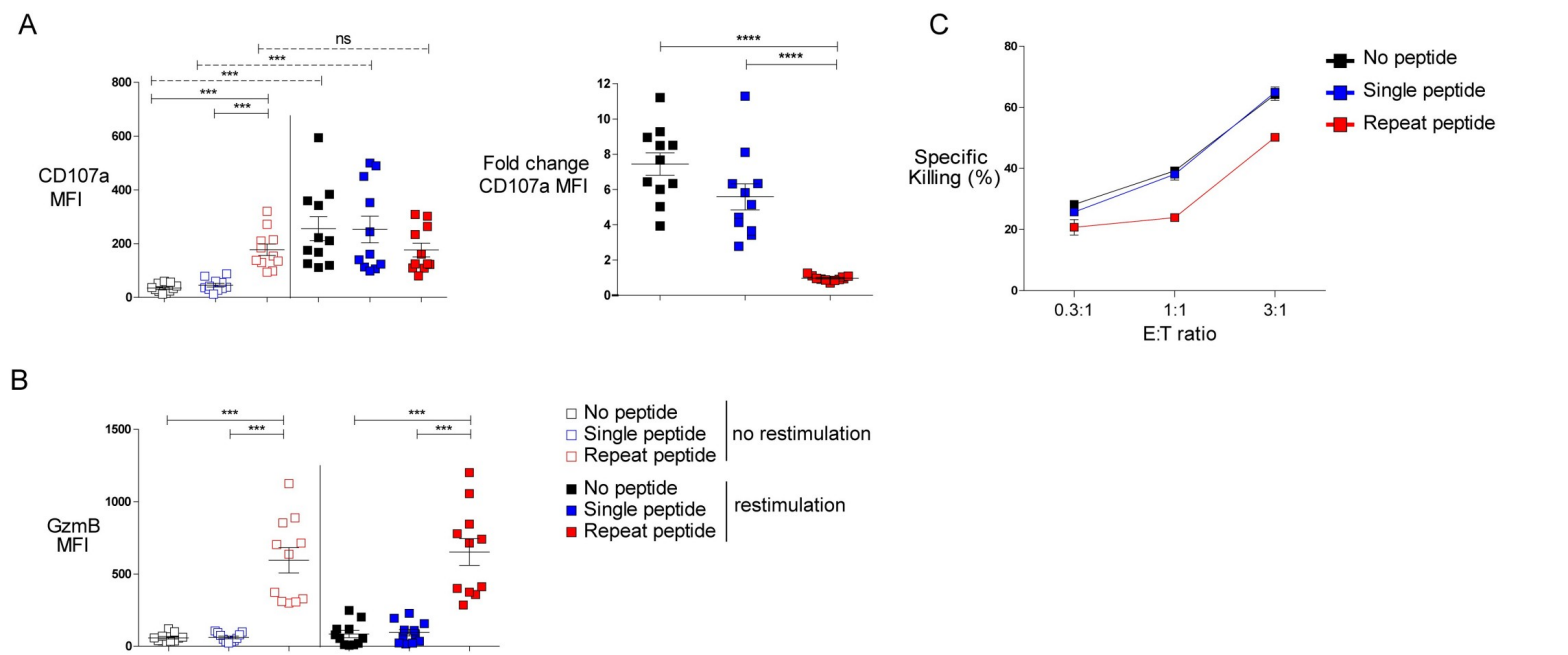
Repeat peptide stimulated CD8+ T cells have decreased cytotoxic function. On day 5, cells were harvested and re-stimulated with OVA peptide. (A) Median fluorescence intensity (MFI) of the degranulation marker, CD107a, shown (left panel). Fold change of CD107a MFI induced by peptide re-stimulation depicted on the right panel. (B) MFI of Granzyme B (GzmB) depicted for the different culture conditions. Each symbol represents one animal (n = 11–12), 9 independent experiments performed. (C) No peptide, single peptide and repeat peptide stimulated cells were co-culture with target cells (OVA-pulsed AE-17 cells) at different ratios. Percentage of specific killing is depicted. One of five independent experiments shown. Line depicts mean ± SE. Between the groups, Student’s t test with Welch’s correction was performed except for CD107a MFI (Wilcoxon signed rank test). *<0.05, **P<0.01, ***P<0.001, ****P<0.0001.

Degranulation is an important step for the CD8+ T cell cytotoxicity. We therefore analyzed the degranulation marker CD107a and Granzyme B (GzmB) expression in our cells. When cells were analyzed without peptide re-stimulation, cells either unstimulated or single peptide stimulated were negative for CD107a and GzmB (Fig 3A and 3B), while repeat peptide stimulated cells showed a significantly higher level of both markers (Fig 3A and 3B). When cells were re-stimulated with OVA_(257–264)_ peptide, cells cultivated in the presence of no peptide and single peptide stimulated cells upregulated CD107a (Fig 3A). In contrast, repeat peptide stimulated cells failed to increase CD107a expression after re-stimulation, indicating that these cells are not able to degranulate further upon re-stimulation. Granzyme B was increased in repeat peptide stimulated cells in agreement with the terminal differentiation of exhausted cells (Fig 3B). No increase of GzmB was detected after 6 hours of re-stimulation for any of the conditions analyzed (Fig 3B). Repeat peptide stimulated cells exhibited reduced cytotoxic capacity against OVA_(257–264)_ peptide-loaded tumor cells (Fig 3C). These findings clearly show that after repeat stimulation *in vitro*, CTL lose their ability to make cytokines, have reduced polyfunctionality, cannot degranulate further, have increased GzmB and reduced cytotoxicity. All of these features are characteristics of the dysfunctional state of *in vivo* exhausted CTL.

### Multiple inhibitory receptors were upregulated following repeat peptide stimulation

**Fig 4:**
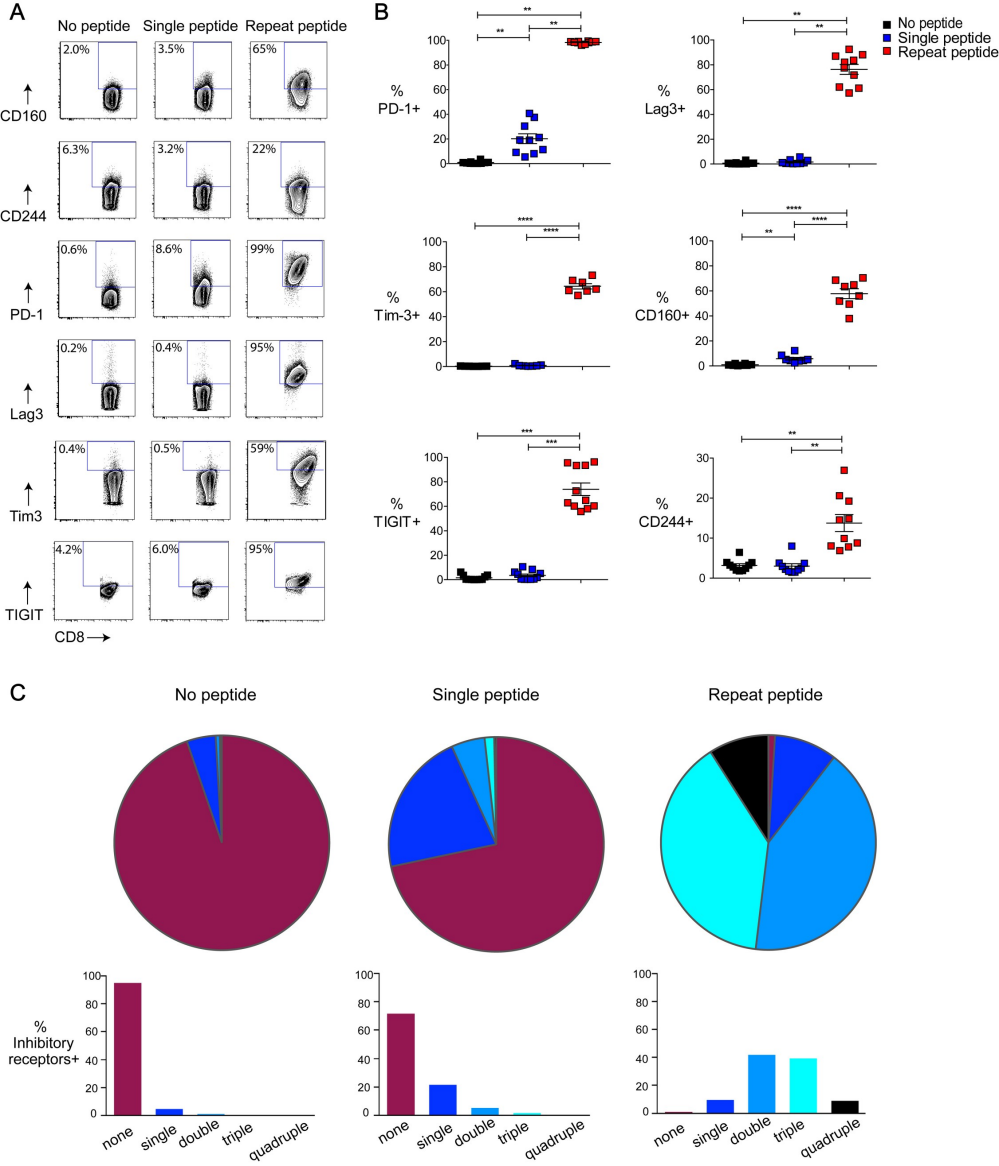
Repeat peptide stimulated cells express multiple inhibitory receptors. The expression of the inhibitor receptors PD-1, CD244, CD160, Lag3 were determined by flow cytometry on day 5 of *in vitro* culture of OT-I CD8+ T cells. Cells were cultured either without peptide (no peptide), one time OVA peptide stimulation (single) or daily peptide stimulations (repeat peptide). (A) Representative flow cytometry plots depicting the frequency of live CD8+ T cells expressing inhibitory receptors. (B) Pooled data of frequency of CTL expressing individual inhibitory receptors within the different culture conditions. (C) SPICE figures depicting the frequency of CD8+ T cells expressing one, two, three or four inhibitor receptors (PD-1, CD244, CD160, Lag3) simultaneously. Each symbol representative one animal (n = 7–11), with 7–9 independent experiments performed. Line depicts mean ± SE. Between the groups, Wilcoxon signed rank test was performed with exception of % Tim-3+ and % CD160+ for which a Student’s t test with Welch’s correction was used. *<0.05, **P<0.01, ***P<0.001, ****P<0.0001.

An increase in inhibitory receptor expression corresponds to a more exhausted state [17]. After harvesting on day five, the cells were stained immediately for the surface expression of the inhibitory receptors PD-1 (CD279), CD244, CD160, Lag3 (CD223), Tim-3 (CD366) and TIGIT. As expected, inhibitory receptors were barely expressed on the cells without peptide stimulation and single peptide stimulated cells (Fig 4A and 4B), except for PD-1, where 20±4% of the single peptide stimulated cells expressed this inhibitory receptor. In contrast, there was a significant upregulation of multiple inhibitory receptors on repeat peptide stimulated cells. Almost all of these cells became PD-1 positive (98±0.3%, Fig 4A and 4B). Furthermore, 74±5% and 76±4% of the cells, respectively, express TIGIT and Lag3. The expression of Tim3 (64±2%), CD160 (58±4%) and CD244 (14±2%) was also significantly increased on the repeat peptide stimulated cells.

We next examined the simultaneous co-expression of multiple inhibitory receptors (PD-1, Lag3, CD160 and CD244) using SPICE (Fig 4C). Repeat peptide stimulated cells were 39% double positive for the inhibitory receptors and another 39% of the cells co-expressed three of these inhibitory receptors simultaneously. Furthermore, 11% of the cells expressed all 4 inhibitory receptors (Fig 4C). In contrast, very few of the no peptide and single peptide stimulated cells expressed two or more inhibitory receptors (Fig 4C). The differences in inhibitory receptor expression remained even after resting repeat peptide stimulated cells for 3 days (S1B Fig).

To exclude that differences in inhibitory receptor expression were due to a different activation status, all the cells were re-stimulated for 6 hours after harvesting them on day 5. Although reactivation induces a slight upregulation of some inhibitory receptors on the no peptide and single peptide cultures, they still remain much lower than the repeat peptide stimulated cultures (S2A Fig). The exception was CD160 which was upregulated to similar levels in all cells. Overall, these findings confirm that multiple inhibitory receptors are expressed on the repeat peptide stimulated cells.

### Expression of transcription factors is altered in repeat peptide stimulated cells

**Fig 5:**
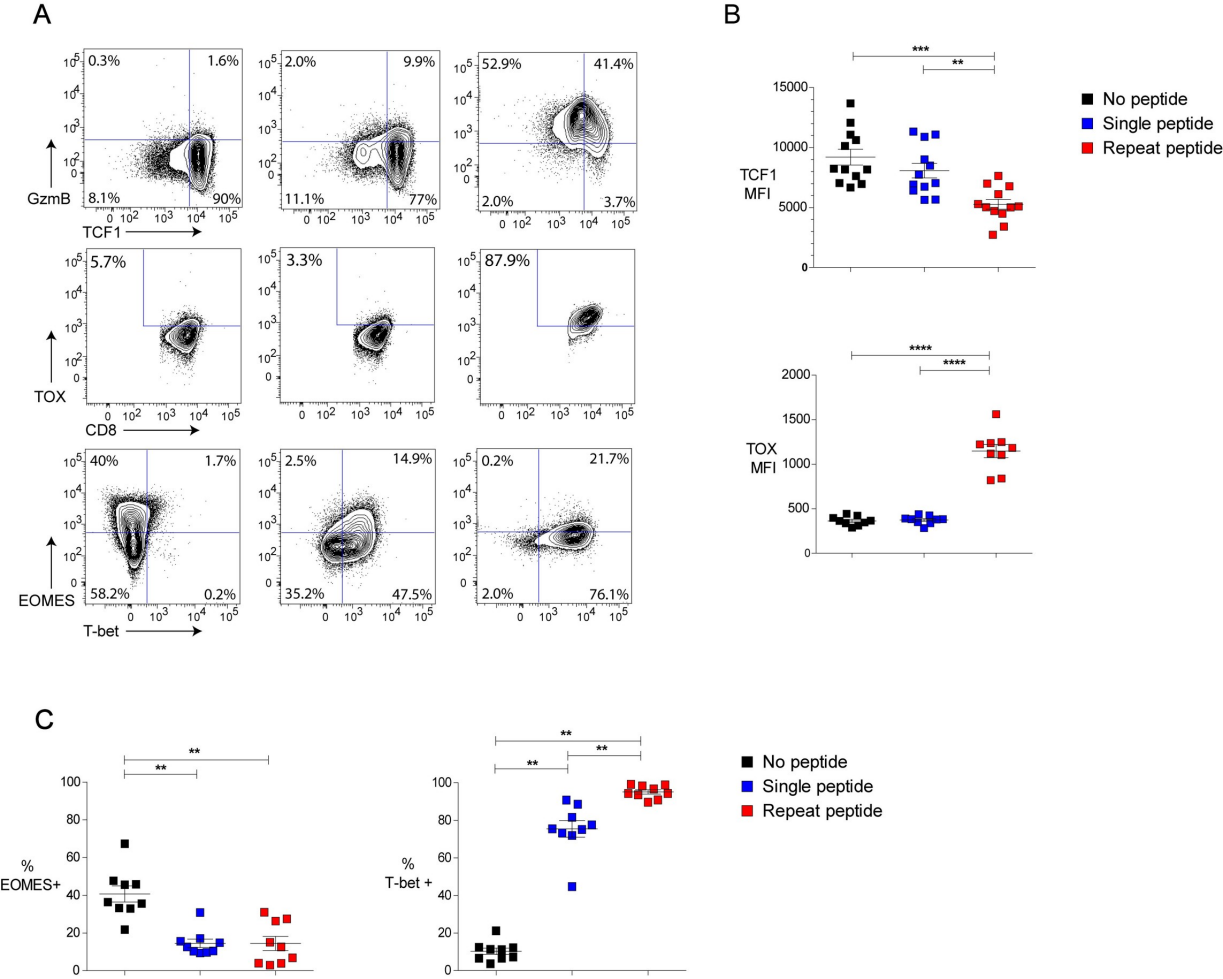
Repeat peptide stimulation induces a distinct pattern of transcription factor expression. Transcription factors (TF) were analyzed in OT-I CD8+ T cells on day 5 of culture. Cells either unstimulated (no peptide), stimulated one time (single peptide) or daily (repeat peptide) shown. (A) Representative flow cytometry plots of the frequency of TF-expressing live CD8+ T cells shown for the differently treated cultures. (B) Pooled data depicting the MFI of TCF1 and TOX in live CD8+ T cells. (C) Pooled data showing the frequency of EOMES and T-bet expressing live CD8+ T cells. Each symbol represents one animal (n = 9–12), with n = 7–9 independent experiments performed. Line depicts mean ± SE. ANOVA with Tukey’s post hoc test was performed on TCF1 MFI, Student’s t test with Welch’s correction was used for TOX MFI and Wilcoxon signed rank test for data in (C). *<0.05, **P<0.01, ***P<0.001, ****P<0.0001.

Previous studies have reported that exhausted CTL express and utilize transcription factors (TF) differently compared to effector and memory cell [11, 12, 40]. To characterize the expression pattern of TF in the *in vitro* exhausted cells, four of the core TFs (TCF1, TOX, T-bet, and EOMES) were measured. TCF1 has been reported to play a critical role in identifying subsets of exhausted T cells [22, 41]. Early exhausted or the progenitor exhausted cells maintained TCF1 expression, while terminally exhausted T cells downregulate its expression. In comparison to single peptide stimulation, repeat peptide stimulation induced down-regulation of TCF1 expression and upregulation of GzmB (Fig 5A and 5B). TOX expression was reported to be increased on exhausted cells [2, 23–25, 40], and indeed we also found TOX to be significantly upregulated in the repeat peptide stimulated cells in comparison to unstimulated and single peptide stimulated cells (Fig 5A and 5B). These differences in TOX and TCF1 expression persisted even after resting 5 day cultures for another 3 days (S1C and S1D Fig) but also after reactivation of cells (S2B and S2C Fig). T-bet and EOMES expression in exhausted CD8+ T cells were dependent on the stage of exhaustion [1, 42]. Although *in vitro* single peptide stimulation already increased the expression of T-bet, repeat peptide stimulation further significantly upregulated T-bet (Fig 5A and 5C). Around 90% of the repeat peptide stimulated cells were T-bet+/PD-1+ double positive whereas in single peptide simulated cells T-bet+ cells were mostly PD-1 negative (S2D and S2E Fig). *In vitro* peptide stimulation downregulated EOMES, with no detectable difference of EOMES expression comparing repeat with the single peptide stimulated cells (Fig 5A and 5C). The increased TOX and T-bet accompanied by decreased TCF-1 expression in repeat peptide stimulated CTL is in agreement with the TF profile of *in vivo* exhausted CTL [25, 41, 42].

### Repeat peptide stimulated cells have decreased *in vivo* expansion capacity

**Fig 6:**
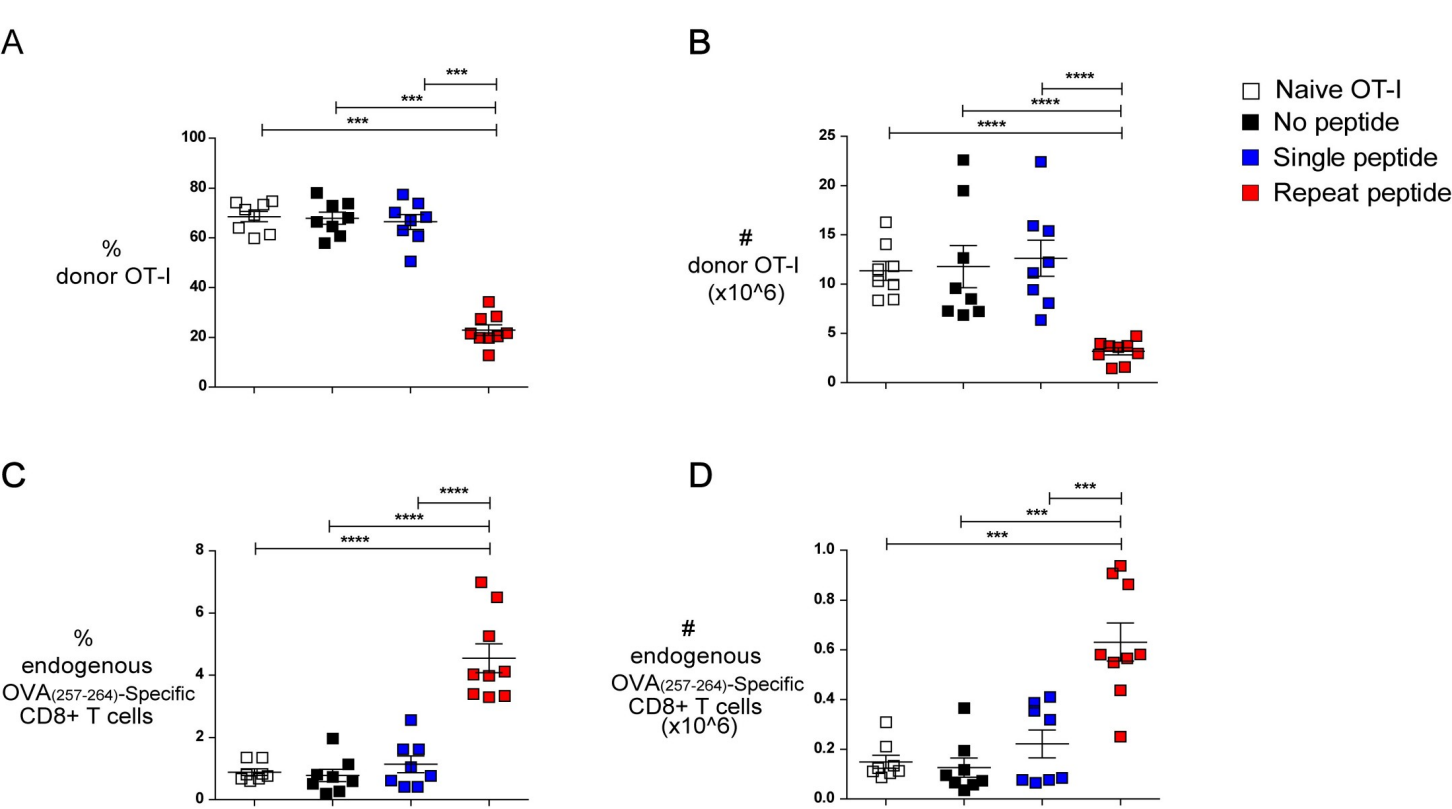
Repeat peptide stimulation decreases *in vivo* CTL expansion capacity. OT-I CD8+ T cells were cultured without peptide stimulation (no peptide), one-time stimulation (single peptide) or daily stimulation (repeat peptide) and sorted on day 5. Live CD8+ T cells were adoptively transferred into wild type mice which were then infected with the OVA_(257–264)-_expressing influenza virus WSN-OVA. Freshly isolated OT-I CD8+ T cells from a naïve mouse were also transferred (naïve OT-I). Lungs were harvested on day 10 post infection. (A) Frequency of donor CD45.1+ OT-I CD8+ T cells within total lung CD8+ T cells shown. (B) Absolute number of lung donor CD45.1+ OT-I CD8+ T cells depicted. (C) Frequency of endogenous CD45.2+ OVA-specific CD8+ T cells within the total lung CD8+ T cells shown. (D) Absolute number of endogenous CD45.2+ OVA-specific CD8+ T cells shown. Each symbol represents one animal (n = 8–9) from n = 3 independent experiments. Line depicts mean ± SE. To determine significant differences between the different animal groups, a Mann-Whitney U test was used except for data in (A) (ANOVA with Tukey’s post hoc test). ***P<0.001, ****P<0.0001.

To evaluate the *in vivo* expansion capacity of repeat peptide stimulated cells, we sorted live CD8+ T cells from *in vitro* cultures (no peptide, single peptide and repeat peptide stimulated cells) and adoptively transferred 10^4^ live CD45.1+ CD8+ T cells into wild type CD45.2+ mice that were infected 3h later with WSN-OVA influenza virus. Freshly isolated naïve CD8+ T cells from an OT-I spleen were also transferred as controls. At day 10 post infection, mice were harvested and the number of donor CD45.1+ cells in the lung were measured. We found a significant reduction in the frequency (23% versus 68% and 66%, for repeat peptide, single peptide and no peptide, respectively, Fig 6A) as well as absolute cell numbers (3.2x10^6^, 11.8x10^6^ and 12.6x10^6^, for repeat peptide, single peptide and no peptide respectively, Fig 6B) for the repeat peptide stimulated cells compared to all controls. Freshly isolated naïve cells did not differ from cells of single and no peptide cultures. The numbers of repeat peptide stimulated donor cells were also reduced in mediastinal lymph nodes (MLN) and spleens (S3 Fig). This indicated that repeat peptide stimulated cells are less capable of expanding after being exposed to their specific antigen. Meanwhile, the mice that received the repeat peptide stimulated cells possessed a larger frequency and number of endogenous CD45.2+ OVA_(257–264)_-specific CTL in their lungs than mice that received unstimulated or single peptide stimulated cells (frequency: 4.5% versus 0.8% and 1.1%, Fig 6C; cell numbers: 0.6x10^6^ versus 0.1x10^6^ and 0.2x10^6^, for repeat peptide, single peptide and no peptide respectively, Fig 6D). This indicates that the adoptively transferred repeat peptide stimulated cells were less efficient than the other donor cells to compete with the endogenous antigen-specific CTL response. These results indicated that *in vitro* repeat peptide stimulation resulted in a reduced capacity of CTL to expand when re-exposed to their cognate antigen. This further supports our notion that *in vitro* repeated antigen stimulation results in exhaustion of these cells.

### Repeat peptide stimulated cells have the transcriptional profile of exhausted CTLs

**Fig 7:**
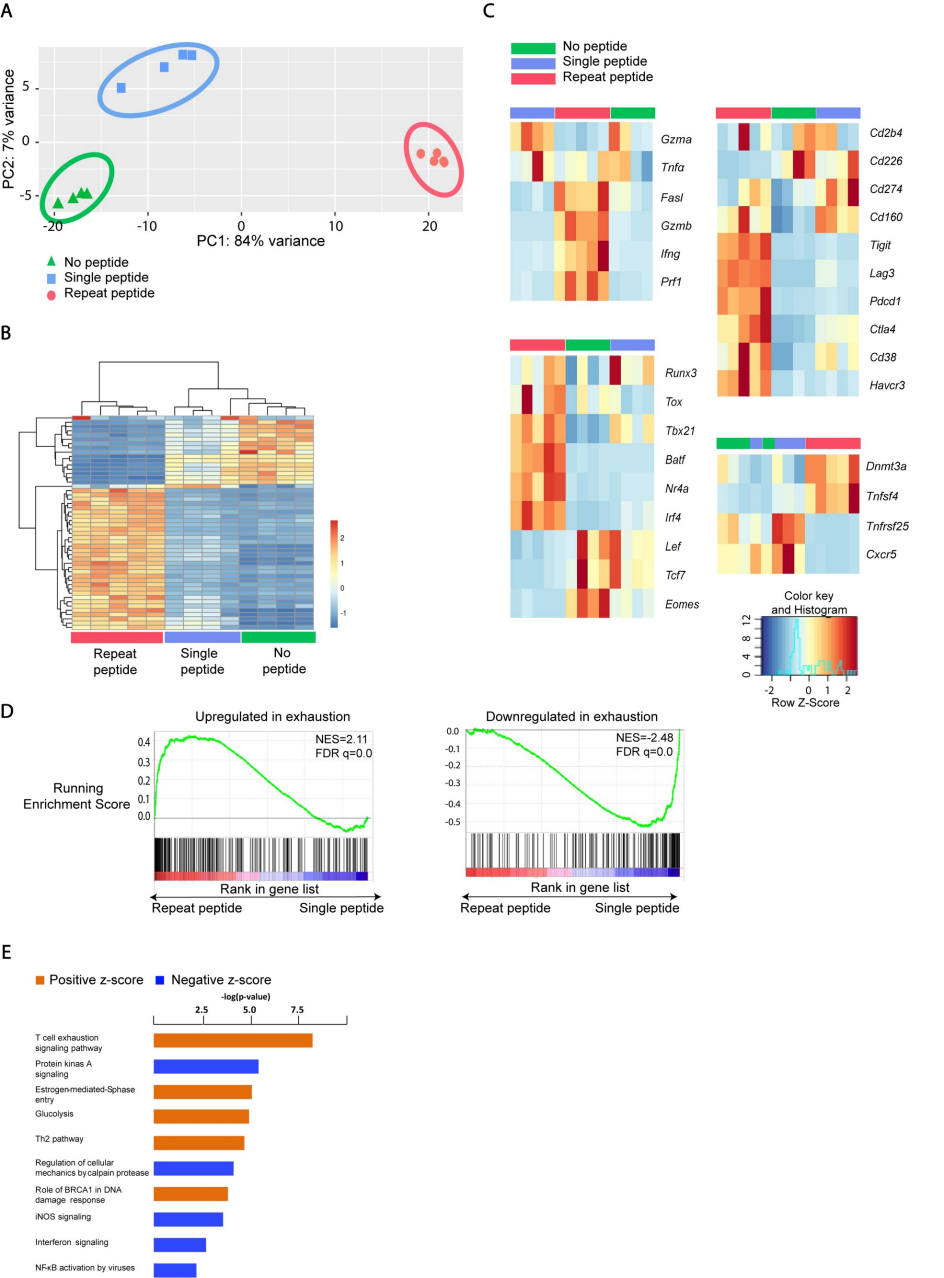
Repeat peptide stimulated cells have distinct transcriptional profile. RNAseq analysis was performed on live CD8+ T cells sorted from 5 day culture of OT-I CD8+ T cells. Cells were either without peptide (no peptide), one-time stimulation (single peptide) or daily peptide stimulations (repeat peptide). (A) PCA plot of RNA-seq data show that cells from identical culture conditions cluster together and away from each other. (B) Heatmap shown for the top 50 differentially expressed genes within CTL from the different culture conditions. (C) Heatmaps for the individual genes clustered based on function presented. (D) Repeat peptide stimulated CTL transcriptomes were enriched in gene signatures of LCMV-Cl13 exhausted CTL. Differentially expressed genes in repeat peptide stimulated CTL were analyzed by GSEA for their enrichment in gene sets found in exhausted CTL from LCMV-Cl13 infected mice. Enrichment in upregulated (left) and downregulated (right) genes shown. (E) Ingenuity pathway analysis was performed on the differentially expressed genes in repeat peptide stimulated cells (compared to single peptide). Significantly upregulated pathways (orange) and downregulated pathways (blue) in repeat peptide stimulated cells are depicted. P values (hypergeometric test) are presented as -log10.

To determine whether repeat peptide stimulated cells also have a distinct transcriptional profile, we performed RNAseq on sorted live CD8+ T cells from the different culture conditions. As shown in the principle component analysis (PCA) (Fig 7A), the samples clustered together and where distinctly separated depending upon their *in vitro* treatment. We identified 1196 genes with more than 2-fold increased expression and 1218 genes with more than 2-fold downregulation in the repeat peptide stimulated cells relative to that in single peptide stimulated cells. The transcriptional prolife of the repeat peptide stimulated cells is clearly more distinct than those of the single and non-stimulated cells (Fig 7B and S1 Table). Among the upregulated genes, multiple inhibitory receptor encoding genes, including PD-1 (*Pdcd1*), *Lag3*, Tim3 (*Havcr2*), *CD160*, *Tigit* and *CTLA4* were presented on the top of this list. As expected, the genes for the markers of the terminal differentiated effector cells, like *GzmB*, *GzmC* and *Prf1* were also significantly upregulated on the repeat peptide stimulated cells (Fig 7C). The transcription factors, *Eomes* and *Tcf7* were downregulated by 4.0 and 6.6 fold, respectively, while *Tox* was found to be 2.9 fold increased in the repeat peptide stimulated cells compared to single peptide stimulated cells (Fig 7C), confirming our flow cytometry findings of these TFs (Fig 5B and 5C). Furthermore, TFs, which are associated with CTL exhaustion such as *IRF4* [28], *NR4a* [29] and *Batf* [30] were also upregulated in the repeat peptide stimulated cells in comparison to single peptide and no peptide stimulated cells (Fig 7C).

To assess whether differentially expressed genes in repeat peptide stimulated cells were enriched for genes that characterize *in vivo* exhausted CTL from LCMV cl13 infections, we performed gene set enrichment analysis (GSEA). Differentially expressed genes between repeat peptide stimulated cell and single peptide stimulated cells were compared to public gene sets of upregulated or downregulated in *in vivo* exhausted CTLs (gene set GSE87646). Gene sets upregulated in exhaustion were found to be significantly more enriched in the repeat peptide stimulated upregulated genes compared to single peptide stimulated cells (Fig 7D). Conversely, the gene sets that were reported to be downregulated in exhausted cells were more enriched in the genes that were downregulated in repeat peptide stimulated cells versus the single peptide stimulated cells (Fig 7D). Thus, GSEA demonstrated significant transcriptional similarity between *in vitro* repeat peptide stimulated cells and *in vivo* exhausted cells. To further investigate signaling pathways that distinguish repeat peptide stimulated cells from single peptide stimulated cells, we performed IPA analysis. The most significant pathway with upregulated activity in repeat peptide stimulated cells was the T cell exhaustion signaling pathway (Fig 7E). The above findings clearly show that *in vitro* repeat peptide stimulation results in the transcriptional changes of *in vivo* exhausted CD8+ T cells from LCMV cl 13 infections which serve as the benchmark of CTL exhaustion.

### Repeat peptide stimulation results in hyper-methylation of the *Tcf7* transcriptional regulatory region

**Fig 8:**
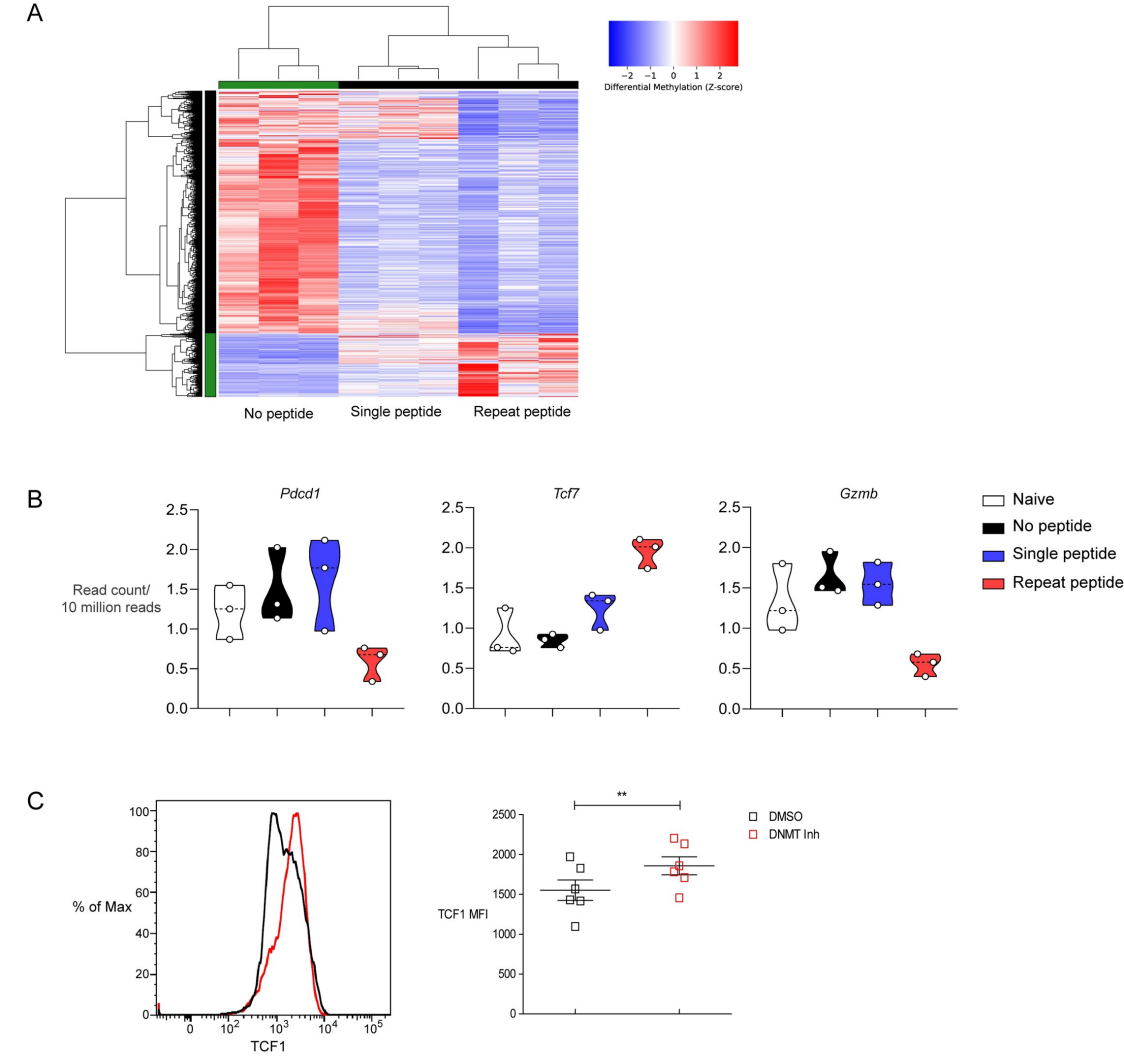
Genome wide DNA methylation changes during T cell stimulation reveal *Tcf7* promotor methylation. Sorted live CD8+ T cells were processed and whole genome methylated DNA sequencing (MeD-seq) was performed. (A) Hierarchical clustering on DMRs (differentially methylated regions) found between the three different peptide exposure conditions are shown. (B) Boxplot of DNA methylation read count data in a 2kb window surrounding the TSS of mentioned genes are shown. The samples were collected from three independent experiments. (C) Representative histogram of TCF1 expression (left) and pooled data showing TCF1 MFI on live CD8 cells (right) in the presence or absence of 20μM DNMT inhibitor during the last 3 days of repeat peptide stimulated cells shown. Data are from n = 6 performed in 3 independent experiments, paired Student’s t test performed. **P<0.01.

Changes in methylation of transcription regulatory region have been described in exhausted CTLs [18, 43]. In order to identify whether *in vitro* repeat peptide stimulated cells also possess similar epigenetic characteristics of exhausted CTLs, whole genome methylated DNA sequencing (MeD-seq) [44] was performed on the sorted live CD8+ T cells. Stimulated T cells undergo distinct genome wide DNA methylation changes depending on the type of treatment (Fig 8A and S2 Table). In comparison to unstimulated and single peptide stimulated cells, the transcriptional regulatory region of *Pdcd1* had significantly less DNA methylation in the repeat peptide stimulated cells (Fig 8B, left). When comparing the promotor methylation status (2kb region surrounding the TSS) of *Tcf7*, there was more DNA methylation detected in repeat peptide stimulated than in the single peptide stimulated cells or unstimulated cells (Fig 8B, center). Meanwhile, in the promotor region of the *GzmB*, significantly less DNA methylation was found in the repeat peptide stimulated cells than in the other cells (Fig 8B, right), which was in line with the higher expression of the protein upregulated in the repeat peptide stimulated cells (Fig 3B). Besides *Pdcd1*, none of the other inhibitory receptor genes’ regulatory or promotor regions were found to possess significant differences in methylation status, although some of them showed expected trends in DNA methylation changes at their TSS. Interestingly, the DNA methylation states of the cytokine genes *IL-2*, *IFN-γ* and *TNF-α* were not significantly different. This indicated that other gene expression control mechanisms, like histone modifications or transcription factor abundance, might regulate the differential expression of these cytokines. Overall, these findings indicate that the repeat peptide stimulated cells have distinct DNA methylation patterns and reveal that the downregulation of TCF1 expression is accompanied by increased promotor methylation of *Tcf7*.

To further confirm that DNA promotor methylation contributes to the downregulation of TCF1 expression in exhausted cells we treated cells with a DNMT inhibitor for the last 3 days of culture and examined whether TCF1 expression was modified in the repeat peptide stimulation cultures. Indeed, treatment of these cells with DNMT inhibitor resulted in an increase in TCF1 levels in exhausted CTL (Fig 8C). This further supports that DNA methylation plays a role in silencing TCF1 as exhausted cells progress from “progenitor exhausted” to the “terminally exhausted” subpopulation.

DNMT inhibitor also reduced the expression of PD-1 and Tim3 in repeat peptide stimulated cells (S4 Fig). Because PD-1 and Tim3 expression are controlled by promoter region DNA methylation [43, 45], our findings suggest that DNMT inhibitor prevented exhaustion rather than reverted the exhaustion of T cells. These experiments suggest that the *in vitro* exhaustion system we describe can be used to test reagents that modulate T cell exhaustion.

### Anergy is not a feature of in vitro and *in vivo* exhausted CTL

The high purity of T cells in our culture system raises the question whether the absence of CD28 costimulation induces anergy [46–48] and this explains some of the phenotypes we observe. However, this is unlikely as our cells are cultured in the presence of IL-7 that has been suggested to prevent anergy [49] and furthermore we still see exhaustion when cells are cultured with IL-2 (S5A and S5B Fig) another anergy preventing cytokine [50, 51]. Some CD28 costimulation, however, may be present in our culture system, as repeat peptide stimulated cells express CD80 mRNA levels 74-fold and 13-fold higher compared to unstimulated and single peptide stimulated cultures, respectively. Flow cytometry confirmed the high surface expression of CD80 on repeat peptide stimulated cells (S5C Fig).

To further exclude that our repeat peptide stimulated cells have features of anergy, we performed GSEA for sets of genes identified by *in vitro* anergy induction (gene set GSE5960) [46]. We find that both genes that are upregulated and downregulated in anergic T cells, are enriched in our repeat peptide stimulated cells (S6A and S6B Fig). Thus there is no evidence for the presence of anergy. We found a similar enrichment for genes both upregulated and downregulated in anergic T cells in *in vivo* exhausted gp33-specific CTL from LCMV clone 13 infected mice (gene set GSE41867) [12] (S6C and S6D Fig).

Finally, we performed pathway enrichment analysis by IPA on the genes differentially expressed between repeat peptide stimulated cells and the unstimulated cells. We found that anergy related signaling pathway was neither up- or down-regulated in the repeat peptide stimulated cells (S6E Fig). In this comparison, the T cell exhaustion signaling pathway was again the most significant upregulated pathway. Taken together, these findings argue that anergy is not a major feature of our *in vitro* exhausted CTL.

## Discussion

Persistent antigen stimulation is a key driver of T cell exhaustion [37, 38]. In this study, we show that repeat stimulation with peptide is sufficient to induce T cell exhaustion *in vitro*. Repeat peptide stimulation rapidly, within 5 days, induced all characteristics of CTL exhaustion including loss of cytokine production and polyfunctionality, the expression of multiple inhibitory receptors, reduced expansion capacity and competitive fitness, transcription factor and gene expression patterns all compatible with *in vivo* generated exhausted CTL [10, 11]. This phenotype was largely preserved when cells were rested for several days. Our *in vitro* repeat peptide stimulation culture conditions include cytokines IL-7 and IL-15 which are known to have anti-apoptotic potential and ensure T cells do not undergo activation induced cell death (AICD) [52]. In particular, IL-15 has been shown to promote the survival of exhausted HIV-specific CD8+ T cells by upregulating Bcl-2 and Bcl-xL [7, 53]. Its inclusion in the repeat peptide stimulations, we argue, promotes the survival of these cells as they become gradually more exhausted while avoiding the increased apoptosis mediated by IL-2, a known promotor of T cell AICD [54].

The *in vitro* exhausted CTL we generate have many of the molecular features of exhaustion. In particular transcription factors that associate and orchestrate T cell exhaustion are clearly modulated in the same manner as seen with *in vivo* induced CTL exhaustion of chronic infection models like LCMV clone 13 infections and cancer [11, 31]. The transcription factor TOX is known to be increased in *in vivo* exhausted CTL [23, 24, 26], while TCF-1 is downregulated in exhausted cells and its presence is associated with precursor exhausted CTL that retain some proliferative capacity [11, 40]. All *in vitro* exhausted CTL we generate have high protein expression for TOX and *Tox* mRNA is upregulated by 2.6-fold. Furthermore, *in vitro* exhausted CTL have downregulated TCF-1 and at the mRNA level its gene *Tcf7* is decreased by 6.6-fold. A recent report indicated that *Prdm1* and *c-maf* transcription factors control a co-inhibitory module in T cells [55]. Although we found that *Prdm1* expression was upregulated by 4.5-fold in our *in vitro* exhausted CTL, we did not find any changes in *c-maf* transcription factor. Activated protein C receptor (PROCR) and podoplanin (PDPN), recently reported to be co-inhibitory receptors [55], were not upregulated in our *in vitro* exhausted cells. This may not be surprising as these markers were only found in tumor infiltrating T cells and IL-27 may be mediating this profile [55]. Exhausted T cells from chronic infection however are not affected by IL-27 signaling [56] and thus PROCR and PDPN expression may depend on the tumor microenvironment and not characterize all exhausted CTL. Our *in vitro* exhaustion method generates exhausted T cells which may more closely reflect the core features of exhaustion. Our method is independent of environmental factors that may occur in the *in vivo* setting of chronic infection or tumor microenvironment. Such factors can obscure direct exhaustion related transcriptional changes from those induced by bystander effects such as high viral loads.

Our *in vitro* exhaustion method utilizes peptide-stimulated purified CD8+ T cells and therefore one could question whether TCR stimulation in the absence of costimulation induces anergy in our T cells. Although, T cell anergy and exhaustion may share some traits and potential signaling pathways of unresponsiveness to restimulation, the mechanisms of their induction is very different. Analyzing gene expression and signaling pathways we found no evidence for anergy in our *in vitro* exhausted CTL. Similar data were obtained with *in vivo* exhausted CTL. The reason we do not induce anergy in our cultures is because they are carried out in the presence of IL-7 and IL-15. It is well established that γc chain cytokines such as IL-2 and possibly IL-7 and IL-15 can override the induction of anergy when CD8+ T cells are stimulated in the absence of CD28 costimulation [47, 49–51]. Cells were exhausted even when cultured with IL-2, albeit IL-2 in these cultures increases cell death significantly. Interestingly, our *in vitro* exhausted CTL express high levels of mRNA and surface CD80 and this may also contribute to preventing anergy. This is not surprising as activated mouse T cells can express CD80 both *in vitro* but also *in vivo* in autoimmune mice [57, 58]. Thus *in vitro* exhausted CTL do not present key anergy traits although one cannot exclude that some downstream signaling or inhibitory pathways are shared between exhaustion and anergy.

Previous studies have reported that there are subsets of exhausted CTLs, one known as “progenitor exhausted” cells which express TCF1 and TOX, corresponding to cells that retain the capacity to expand with checkpoint blockade therapy and the other subpopulation known as the “terminally exhausted” cells which are TCF1 low and cannot respond to checkpoint blockade [26, 40, 41, 59]. In our *in vitro* exhausted cell cultures, we observed both subsets; a subset of cells that express low levels of TCF1 and high levels of TOX and GzmB, which is in line with the characteristics of “terminally exhausted” T cells and a subset that is TCF1+TOX+ corresponding to the “progenitor exhausted” cells. All cells are T-bet^hi^ PD1+, thus our TOX+ cells may retain some “progenitor exhausted” characteristics as T-bet^hi^ cells are reported to retain proliferative capacity in the exhaustion setting [42]. Thus, these two subsets exist in our exhausted cultures and this allows us to perform future in depth characterization of these cells and establish their relationship and the factors that control them. CXCR5+ T cells were described as “less exhausted” and responders to check-point blockade in chronic viral infection [21, 59]. Although “progenitor exhausted” TCF1+TOX+ are present in our repeat peptide stimulated T cells, we could not detect any CXCR5 expression. CXCR5+ exhausted CD8+ T cells are found in lymphoid organs but not in peripheral blood or other infected organs of LCMV_clone13_ infected animals [21], and therefore our CXCR5- *in vitro* exhausted cells may resemble more the later than those found in lymphoid organs.

TCF1 downregulation in exhausted cells was reported to be accompanied by reduced chromatin accessibility [24, 25]. Our findings suggest that DNA methylation also contributes to reduced TCF1 expression as both the *Tcf7* promotor was found to be hyper-methylated and the inhibition of DNA methyltransferases during the last 3 days of culture results in the retention of TCF1 expression in our exhausted CTL. Whether these TCF1 high cells generated by DNMT inhibitor treatment retain all the characteristics of the “progenitor exhausted” cell subset remains to be determined. If true it may indicate that such inhibitors can be combined with checkpoint inhibition therapy as such TCF1+ cells are the major responders to therapy.

By studying these *in vitro* exhausted CTLs, the accumulation of inhibitory receptor expression on exhausted cells can be better understood. PD-1, Tim-3 and TIGIT are rapidly expressed on the early exhausted cells, while the other receptors, like CD160 and CD244, were induced more slowly by repeat antigen stimulation. Because of the promising effects of the checkpoint blockade therapy, such as anti-PD1/PD-L1, on the treatment of human cancer [60], there is considerable interest to revive the function of exhausted T cells by co-blockade of multiple inhibitory receptors simultaneously. Our *in vitro* method, which induces multiple inhibitory receptors in just a few days, can serve as a feasible tool to test blocking approaches before performing *in vivo* animal experiments. For example, TIGIT blockade can be tested, because these cells not only upregulated TIGIT, but also downregulated its competitor of the same ligand, CD266, on their surface. Moreover, the underlying mechanisms of checkpoint blockade on reversal of T cell exhaustion need to be further investigated as do the pathways that restrain these cells from developing into terminally exhausted cells. Clearly the balance of these exhausted cells can be modulated with large effects on the numbers and potentially the function of these cells. As we recently have demonstrated, by overexpressing a single miRNA, namely miR-155, we can ameliorate the attrition of virus-specific CTL during chronic infection and *in vivo* increase their numbers by 2 logs [61]. Our *in vitro* system of T cell exhaustion can be used to screen the overexpression of genes with retroviruses or their inhibition or deletion using CRISPR-Cas9 to discover new targets which modulate T cell exhaustion.

The method we describe allows for the rapid, within 5 days, generation of large numbers of fully exhausted CTL and can therefore be used for medium to high throughput screening of compounds, reagents or gene modifications that can prevent, ameliorate, reverse or accelerate T cell exhaustion. In particular, the extreme loss of IL-2 and TNFα production in our repeat peptide stimulated cells allows for the first time a truly medium to high throughput approach for drug screening since changes in IL-2 or TNFα production can be easily determined in the supernatants.

In conclusion, we have established a rapid *in vitro* system of CD8+ T cell exhaustion. These exhausted CTL exhibit all the known molecular and functional characteristics of exhaustion yet can be induced in large numbers within 5 days as opposed to the small numbers generated after 30 days of chronic infection of *in vivo* mouse models of exhaustion. This *in vitro* method can not only be used as a screening system to prevent/revert CD8+ T cell exhaustion, thereby identifying new therapies, but also for research aiming at revealing mechanisms of CD8+ T cell exhaustion. Using this *in vitro* method we show that TCF1 silencing during exhaustion is in part controlled by DNA methylation. Overall, this *in vitro* method makes the future study of CTL exhaustion more feasible and reduces the need for *in vivo* studies.

## Materials and methods

### Mice

OT-I CD45.1+ mice on the C57BL6/J background were generated by backcrossing C57BL/6 Tg (TcraTcrb)1100Mjb/J (OT-I) with B6.SJL-Ptprca Pepcb/BoyJ (CD45.1+) mice (both from Charles River France, a registered vendor of The Jackson Laboratories C57BL/6 mice). C57BL/6J mice and OT-I mice were housed in a certified barrier facility at Erasmus University Medical Center.

### Ethics statement

Animal work was performed under Project Proposal (AVD101002015179) by the animal welfare body (AWB) of the Instantie voor Dierenwelzijn (IvD). All animal experiments were conducted in compliance with the Netherlands’ government laws of the Centrale Commissie Dierproeven (CCD).

### Repeated antigen stimulation *in vitro*

CD8+ T cells were purified from spleens of OT-I mice by negative selection with magnetic beads (EasySep, Stemcell Technologies). After purification, cells were 97.7±0.5% CD8+ T cell and contained 0.11±0.04% CD11b+ CD11c- monocytes and 0.09±0.05% CD11b+ CD11c+ dendritic cells. In each well of a 24-well plate, 5x10^5^ of the purified CD8+ T cells/ml were cultured in complete media (RPMI 1640, 10% FBS (Gibco), 1% 2mM L-glutamine (Life Technologies), 1% HEPES (Life Technologies), 1% 100nM Sodium Pyruvate (Life Technologies), 1% non-essential amino acides (Life Technologies), 100U/ml penicillin (Gibco) and 100μg/ml Streptomycin-sulfate (Gibco), 0.05mM Betamercaptoethanol (Sigma)) with IL-15 (5ng/ml, Peprotech, Cat 210–15) and IL-7 (5ng/ml, Peprotech, Cat 210–07) with or without 10ng/ml OVA_(257–264)_ peptide (Anaspec Cat AS-60193).

For single peptide stimulation, cells were cultured in the presence of OVA_(257–264)_ peptide for 48 hours. The peptide was then removed by washing the cells two times with complete media. For the remaining 3 days, the cells were cultured in the complete media with cytokines. For repeat peptide stimulation, 10ng/ml OVA_(257–264)_ peptide was added daily for five days. The cells were washed also on day 2 to allow for comparable culture conditions. Unstimulated control cells were cultured in media with cytokines but without adding peptide. Cells from all three conditions were checked daily, and when the cells were confluent, they were split and cultured with fresh complete media containing cytokines. After day 5, some of the cell were washed two times with complete media and maintained in the media only with cytokines for another three days. In some experiments cell cultures were treated on day 2 with 20μM DNA methyltransferase (DNMT) inhibitor SGI-1027 (Tocris, Bio-techne) that targets DNA methyltransferases DNMT3B, DNMT3A and DNMT1.

On day five, cells were harvested and counted using an automated counting system (Countess, Life Technologies). Cells were stained with DAPI Viability dye (Beckman Coulter, Cat B30437) and Acridine Orange (Biotium, Cat 40039) to distinguish live and dead cells.

### *In vitro* killing assay

AE17 cells were maintained in RPMI 1640 supplemented with 10% FBS (Gibco), 100 units/mL Penicillin/Streptomycin (Life Technologies), 2 mM L-glutamine (Life Technologies), 0.05 mM 2-mercaptoethanol (Sigma), and were cultured at 37°C in 5% CO2. AE17 cells were pulsed with 1 μg/ml OVA_(257–264)_ Peptide (Anaspec Cat AS-60193) for 1 hour and then the cells were washed thoroughly before they were labelled with the CellTrace Far Red fluorescent dye (ThermoFisher Scientific Cat C34564/15396613). Un-pulsed cells were not labeled. A 1:1 mix of peptide pulsed and un-pulsed AE17 cells (10^5^ each) were mixed and different amounts of T cells (Effector: Target ratios: 3:1, 1:1, 0.3:1) were added. The cells were harvested after 16 hours, the ratio of labeled and unlabeled tumor cell were detected by flow cytometry.

### Flow cytometry

To investigate phenotypic and functional changes, cells were stained with monoclonal antibodies and analyzed using flow cytometry. The following fluorochrome-conjugated monoclonal antibody combinations against surface and intracellular antigens were used; Inhibitory receptors and ligands: anti-CD8-eFluor 450 (53–6.7, eBioscience), anti-Lag3-APC (C9B7W, BD Biosciences), anti-PD-1-APC-Cy7 (19F.1A12, Biolegend), anti-CD244-PE (2B4, BD Biosciences; eBio244F4, eBioscience), anti-Tim3-PE-Cy7 (RMT3-23, Invitrogen), anti-CD160-PE-CF594 (CNX46-3, BD Biosciences), anti-TIGIT-FITC (GIGD7, eBioscience); Activation and differentiation: anti-CD44-BV785 (IM7, BD Biosciences), anti-CD25-APC-Cy7 (PC61, BD Biosciences); Cytokines and effector molecules: anti-IFN-γ-APC (XMG1.2, eBioscience), anti-TNF-α-AF488 (MP6-XT22, eBioscience), anti-IL-2-PE (JES6-5H4, eBioscience), anti-GranzymB-PE-Cy7 (NGZB, eBioscience); Intracellular expression of transcription factors anti-Tbet-PE-Cy7 (4B10, Biolegend), anti-TCF1-APC (C63D9, Cell Signaling), anti-EOMES-PE- eFluor F610 (Dan11mag, eBioscience), anti-Tox-PE (TXRX10, eBioscience). To exclude apoptotic and dead cells, Annexin V conjugated with APC, Cy5.5 or PerCP-Cy5.5 (BD Biosciences) was included in all the stains and 2.5 mM CaCl_2_ was added to all solutions.

On day 5, cells were harvested and immediately stained for surface and intracellular antigens. For surface staining, cells were washed with FACS wash (HBSS containing 3% FBS and 0.02% sodium azide) and incubated with 20μL mix of the pre-determined optimal concentrations of the fluorochrome-conjugated monoclonal antibodies at 4°C in the dark for 20 minutes. The cells were then washed once with FACS wash and fixed with 1% PFA. For the transcription factor staining, cells were first stained for surface antigens as described above. Following the washing step, cells were fixed with FoxP3 Fixation Buffer (005523, eBioscience) for 60 minutes in the dark at 4°C and then washed with Perm/Wash buffer (008333, eBioscience) and stained with a mix of antibodies against transcription factors for 1 hour at 4°C in the dark. Cells were then washed twice with Perm/Wash buffer and fixed with 1% PFA. Appropriate isotype controls were included for staining of transcription factors.

To analyze cytokine production, cells were re-stimulated with the 10μg/ml OVA_(257–264)_ SIINFEKL peptide for 6 hours at 37°C, 5% CO_2_ in the presence of GolgiPlug (BD Biosciences) and anti-CD107a-APC-Cy7 antibodies (ID4B, Biolegend). Cells were then stained with surface marker antibodies as described above. After washing with FACS wash, cells were fixed with IC Fixation Buffer (88–8824, eBioscience) at 4°C overnight, washed with Perm/Wash buffer and stained for intracellular cytokines for 45 min in the dark at 4°C. After staining, cells were washed twice with Perm/Wash buffer and fixed with 1% PFA.

Cells were measured on a LSRFortessa (BD Biosciences) using application settings and at least 200,000 events were collected. Data was then analyzed with FlowJo software (Version 9.9.4, Treestar, Ashland, OR, USA).

### *In vivo* influenza model

For influenza virus infection studies, live CD45.1+ OT-I cells were sorted from *in vitro* cultures on a FACSAria III (BD Biosciences) using fluorochrome-conjugated Annexin V and anti-CD8 antibodies and 10,000 cells were intravenously transferred into 8–12 weeks old CD45.2+ C57BL/6J wild-type recipient mice. Naïve CTL were also freshly isolated from spleen of an OT-I mouse and adoptively transferred. Three hours later, mice were anesthetized with 2.5% isoflurane gas and infected intranasally with influenza virus strain A/WSN/33-expressing OVA (WSN-OVA, a gift from Dr. D. Topham, University of Rochester Medical Center). Body weight was measured daily to track the influenza infection.

Ten days post infection, the lung, spleen and mediastinal lymph nodes were harvested and single cell suspensions were obtained after processing the tissues. As described previously [62], lungs were digested for 2 h at 37°C with 3.0 mg/ml collagenase A and 0.15 μg/ml DNase I (Roche) in RPMI 1640 containing 5% heat-inactivated FBS, 2 mM L-glutamine, 100 IU/ml penicillin, 100 μg/ml streptomycin. Digested lungs were then filtered through a 40-μm cell strainer (Falcon) and washed in the same media as above. PE-conjugated tetramers of H-2K^b^ major histocompatibility complex class I loaded with OVA_(257–264)_ were used to identified the antigen-specific CTLs in the lungs [63].

### RNA sequencing

To compare the gene expression between the different culture conditions, RNA sequencing was performed. On day 5, 1x10^6^ live CD8+ T cells were sorted from the three different culture conditions and immediately lysed with TRIzol LS reagent (Life Technologies) and stored at -80°C. RNA was extracted according to manufacturer’s instructions and a bioanalyzer (Agilent) was used to determine the integrity of the extracted RNA. Barcoded sequencing libraries were generated using the KAPA RNA HyperPrep kit (Roche Diagnostics) with RiboErase (HMR) rRNA depletion. Library quality was assessed with the bioanalyzer and KAPA qPCR was performed for quantification before cluster generation and 100-bp paired-end sequencing on a Hiseq2500 machine (Illumina). Data sets are deposited in the Gene Expression Omnibus (GEO) database repository under accession numbers GSE150120.

### Analysis of differential transcript abundance, normalization of read counts by gene size, and downstream analyses

Four independent biological replicates were analyzed for each condition. The quality of the sequencing was verified using the FastQC software (http://www.bioinformatics.babraham.ac.uk/projects/fastqc/). The demultiplexed fastq files were aligned using STAR software (v.2.5.3e) [64] with default settings and *mus musculus* GRCm38 as alignment reference. Then bam files, generated during alignment, were annotated using FeatureCount software (v1.6.1) [65] to obtain the annotated files (count files). The annotation reference was genecode.vM15.gtf.

Count data was preprocessed to remove low expressed genes. Then, rlog transformation was applied to the clean counts for visualization and comparison purposes which include correlation and clustering analysis generating the heatmaps and PCA plots during the process. For differential expression analysis, DESeq2 (DESeq2 R package, v1.22.2) [66] was used directly in the clean count data.

The list of differentially expressed genes was used to perform Ingenuity Pathway Analysis (IPA, Qiagen, USA version 01–12) to further discern which pathways are involved in the CD8+ T cell exhaustion process. Pathway enrichment P-values (Fisher’s exact test) and activation Z-scores were calculated by IPA and used to rank the significant pathways.

The same list of significantly differentiated genes was used for enrichment analysis (GSEA Desktop Application, v2.2.1). The CD8+ T cell exhaustion gene-sets were downloaded from GMO datasets (gene set GSE87646) based on the publication of Bengsch B, et al [2]). Enrichment analysis in CTL exhaustion genes was determined for the upregulated and downregulated genes separately. Normalized Enrichment Scores (NES) values were used to determine whether an expression gene-set was enriched or not in CTL exhaustion genes. For anergy GSEA analysis, gene set GSE5960 [46] and gene set GSE41867 [12] were used.

### DNA methylation profiling detection

DNA methylation profiling was done as previously described by the MeD-seq method [44]. For MeD-seq sample preparation LpnPI (New England Biolabs) digestions were carried out on DNA samples according to manufacturer’s protocol. Reactions contained 50 ng and digestion took place overnight in the absence of enzyme activators. Digests of genomic DNA with LpnPI resulted in snippets of 32 bp around the fully-methylated recognition site that contains CpG. The DNA concentration was determined by the Quant-iT High-Sensitivity assay (Life Technologies) and 50 ng dsDNA was prepared using the ThruPlex DNA-seq 96D kit (Takara). Twenty microliters of amplified end product were purified on a Pippin HT system with 3% agarose gel cassettes (Sage Science). Stem-loop adapters were blunt end ligated to repaired input DNA and amplified (4 +10 cycles) to include dual indexed barcodes using a high fidelity polymerase to yield an indexed Illumina NGS library. Multiplexed samples were sequenced on Illumina HiSeq2500 systems for single read of 50 base pairs according to manufacturer’s instructions. Dual indexed samples were demultiplexed using bcl2fastq software (Illumina).

MeD-seq data processing was carried out using specifically created scripts in Python version 2.7.5. Raw fastq files were subjected to Illumina adaptor trimming and reads were filtered based on LpnPI restriction site occurrence between 13–17 bp from either 5’ or 3’ end of the read. Reads that passed the filter were mapped to mm10 using bowtie2.1.0. Multiple and unique mapped reads were used to assign read count scores to each individual LpnPI site in the mm10 genome. BAM files were generated using SAMtools for visualization. Gene and CpG island annotations were downloaded from UCSC (MM10). Genome wide individual LpnPI site scores were used to generate read count scores for the following annotated regions: transcription start site (TSS) (1 kb before and 1 kb after), CpG islands and genebody (1 kb after TSS till TES).

MeD-seq data analysis was carried out in Python 2.7.5. DMR detection was performed between two datasets containing the regions of interest (TSS, genebody or CpG islands) using the Chi-Squared test on read counts. Significance was called by either Bonferroni or FDR using the Benjamini-Hochberg procedure. Differently methylated regions were used for unsupervised hierarchical clustering, the Z-score of the read counts was used for normalization and is also shown in the heatmaps. In addition, a genome wide sliding window was used to detect sequentially differentially methylated LpnPI sites. Statistical significance was called between LpnPI sites in predetermined groups using the Chi-squared test. Neighbouring significantly called LpnPI sites were binned and reported, DMR threshold was set at a minimum of ten LpnPI sites, a minimum size of 100 bp and either a twofold or fivefold change in read counts. Overlap of genome wide detected DMRs was reported for TSS, CpG island and gene body regions.

### Statistical analysis

Statistical analyses were performed using Prism software (GraphPad Prism5 for Windows, Version 5.04). The normality of data distribution was assessed using the Shapiro-Wilk normality test. Homogeneity of variance was tested with Bartlett’s test. When data were normally distributed and group variances were equal, an ANOVA with Tukey's Multiple Comparison Test was performed. When data were normally distributed but group variances were unequal, a Student’s t test with Welch’s correction was performed. If data were not normally distributed a Wilcoxon signed rank test or a Mann–Whitney U test was performed. P values equal or lower than 0.05 were considered statistically significant with the numbers of stars in the figures indicating the p value: * = P ≤ 0.05, ** = P ≤ 0.01, and *** = P ≤ 0.001.

## Supporting information

S1 Fig Exhaustion related features are retained after 3 days resting. Cells were harvested on day 5, peptide was washed away and the cells were rested for 3 days without peptide. On day 8, cytokine production after restimulation is shown (A). The expression of inhibitor receptors (B) and transcription factors (C and D) are also shown on day 8. Each symbol represents one animal. Line depicts mean ± SE.
(TIF)

S2 Fig Repeat-peptide stimulated cells maintain inhibitory receptor and transcription factor differences after 6 hours of restimulation. After extra 6 hours of stimulation with OVA peptide (10μg/ml) on day 5, the percentage and MFI of inhibitory receptors (A) and the MFI of TCF1 (B) and TOX (C) are depicted. Representative FACS plots (D) and pooled data (E) of the frequency of PD-1 and T-bet co-expression on day 5 shown. Line depicts mean ± SE. Each symbol represents one animal. Data from 5–6 experiments.
(TIF)

S3 Fig Repeat peptide stimulated cells expanded less in mediastinal lymph nodes and spleens *in vivo*. OT-I CD8+ T cells were cultured without peptide stimulation (no peptide), one-time stimulation (single peptide) or daily stimulation (repeat peptide) and sorted on day 5. Live CD8+ T cells were adoptively transferred into wild type mice which were then infected with the Ova_(257–264)_-expressing influenza virus WSN-OVA. Freshly isolated OT-I CD8+ T cells from a naïve mouse were also transferred (naïve OT-I). Mediastinal lymph nodes (MLN) and spleens were harvested on day 10 post infection. Frequency of donor OT-I CD8+ T cells within spleen (A) and MLN (C) in total CD8+ T cells and the absolute number of donor cells in spleen (B) and MLN (D) are presented. Each symbol represents one animal (n = 8–9) from n = 3 independent experiments. Line depicts mean ± SE. To determine significant differences between the different animal groups, Mann-Whitney U test was used except for data in (A) (ANOVA with Tukey’s post hoc test). *P<0.05, ** P<0.01, ***P<0.001, ****P<0.0001.
(TIF)

S4 Fig DNMT inhibitor decreases expression of inhibitor receptors on the repeat peptide stimulated cells. Pooled data showing day 5 inhibitor receptor MFI on repeat stimulated cells in the presence or absence of 20μM DNMT inhibitor. Inhibitor was added during the last 3 days of culture. Data are from n = 6 animals, performed in 3 independent experiments. Line depicts mean ± SE. To determine significant differences between the different treatment, paired t-test was used *P<0.05, ** P<0.01, ***P<0.001.
(TIF)

S5 Fig Exposure to IL-2 does not alter the exhaustion phenotypes in repeat peptide stimulated cells and CD80 is upregulated on repeat stimulated cells. CD8+ T cell exhaustion was induced in the presence of IL-2 (20U/ml) and IL-7/IL-15 (5ng/ml each). Cytokine production after OVA peptide restimulation for 6 hours (A) and inhibitory receptor expression on day 5 (B) are shown. n = 8 animals from 5–6 independent experiments depicted. Representative histogram of CD80 expression on the cells is shown in (C). One of two independent experiments shown (n = 3).
(TIF)

S6 Fig Anergy gene sets and pathways do not characterize repeat peptide exhausted T cells. Both upregulated and downregulated genes in anergic T cells are enriched in exhausted T cells. (A) GSEA of genes upregulated and (B) downregulated in anergic T cells (gene set GSE 5960) are both enriched in differentially expressed repeat peptide stimulated cells (repeat peptide versus no peptide cell). (C) GSEA of genes upregulated and (D) downregulated in anergic T cells (gene set GSE 5960) are both enriched in differentially expressed Day 30 gp33-specific CTL from LCMV clone 13 infected animals (Day 30 versus gp33-specific CTL naïve CD8+ T cells; data from GSE41867). (E) In repeat peptide stimulated cells the anergy related pathway in IPA analysis was neither upregulated nor downregulated. IPA analysis was performed on the differentially expressed genes in repeat stimulated cells (compared to no peptide). The “Regulation of IL-2 expression in activated and anergic T lymphocytes” pathway is showed enlarged and the overall positioning in the global IPA pathway analysis is indicated by the arrow and red circle. Significantly upregulated pathways (orange) and downregulated pathways (blue), no activity pattern available (grey) are depicted. Bar graphs depict P values (hypergeometric test) presented as–Log10.
(TIFF)

S1 Table Differentially expressed genes in repeat peptide stimulated T cells determined by RNAseq. Log2 fold change is show for repeat peptide OT-I versus single peptide stimulated OT-I. After 5 day cultures, cells were sorted and RNAseq was performed. Genes with >2 fold change and padj value < 0.05 shown.
(PDF)

S2 Table Differentially methylation of TSS regions was determined by MeD-seq. Fold change is show for repeat peptide OT-I versus single peptide stimulated OT-I. After 5 day cultures, cells were sorted and MeD-seq was performed. Greater than 2-fold changes shown.
(PDF)
