# Supplementary figures and images for: Rapid in vitro generation of bona fide exhausted CD8+ T cells is accompanied by Tcf7 promotor methylation
Source: PLoS Pathog. 2020 Jun 24;16(6):e1008555. doi: 10.1371/journal.ppat.1008555 (PMC7340326; doi:10.1371/journal.ppat.1008555)

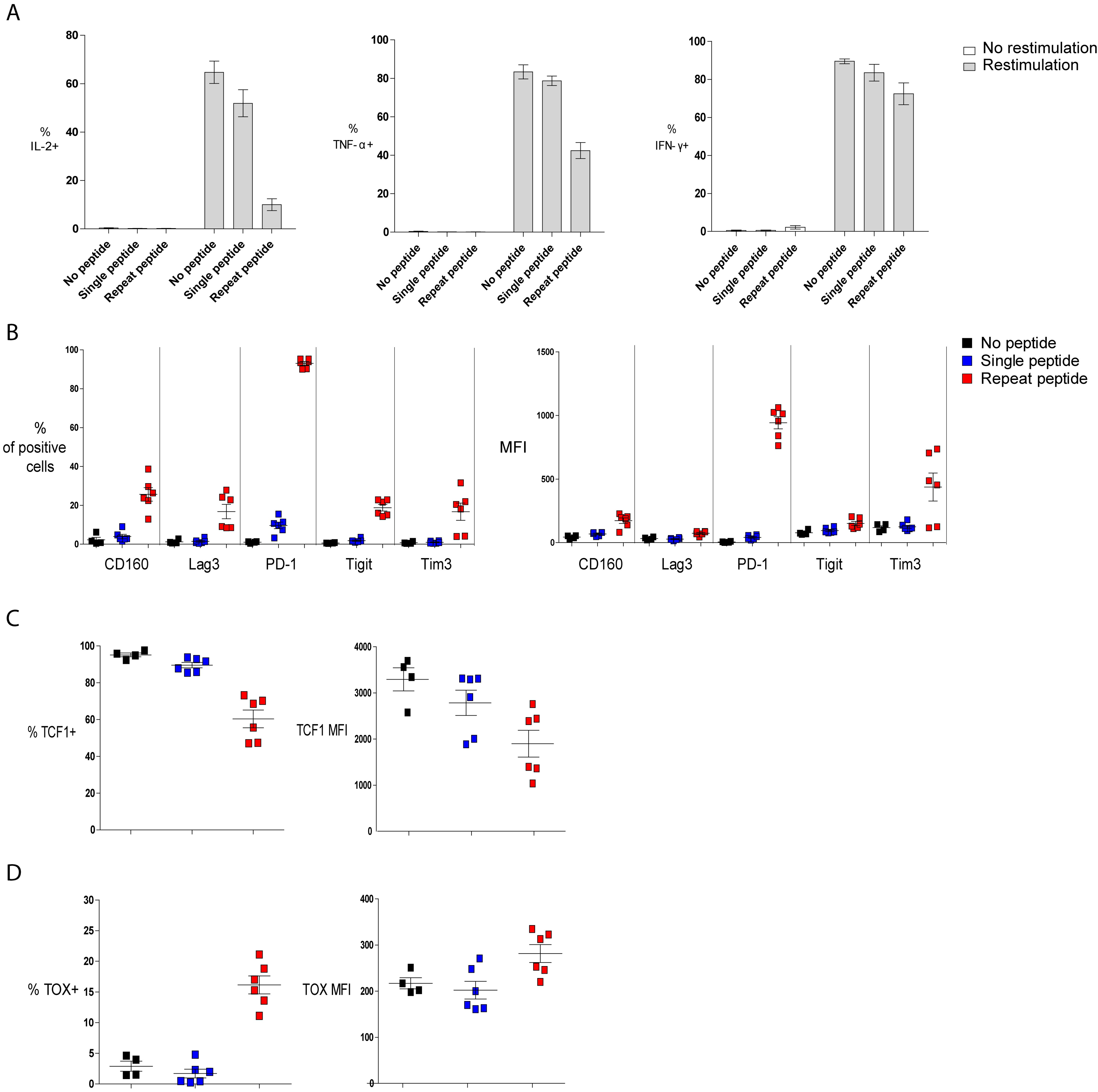

Supplement: S1 Fig — Cells were harvested on day 5, peptide was washed away and the cells were rested for 3 days without peptide. On day 8, cytokine production after restimulation is shown (A). The expression of inhibitor receptors (B) and transcription factors (C and D) are also shown on day 8. Each symbol represents one animal. Line depicts mean ± SE. (TIF) [file ppat.1008555.s001.tif]

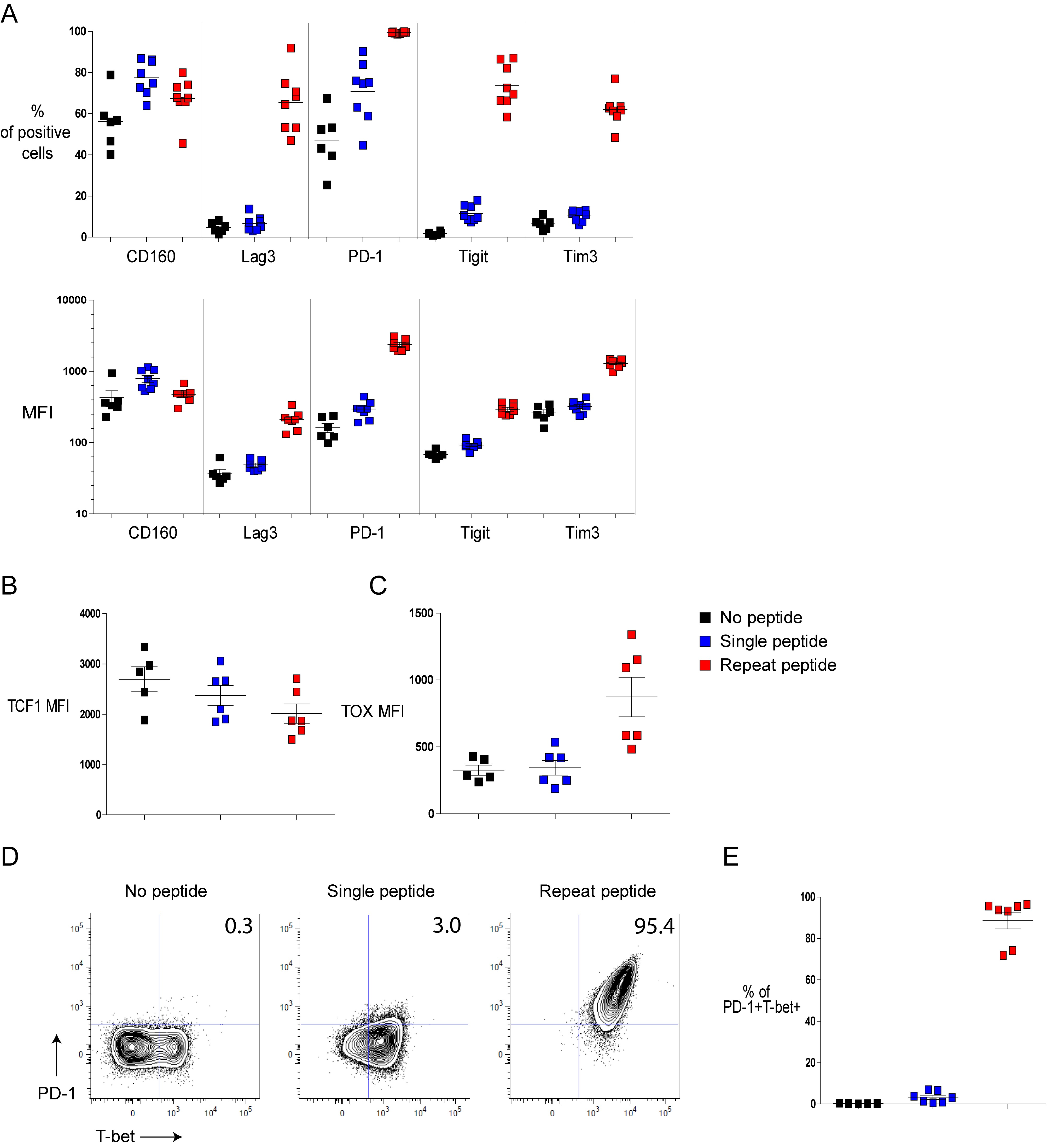

Supplement: S2 Fig — After extra 6 hours of stimulation with OVA peptide (10μg/ml) on day 5, the percentage and MFI of inhibitory receptors (A) and the MFI of TCF1 (B) and TOX (C) are depicted. Representative FACS plots (D) and pooled data (E) of the frequency of PD-1 and T-bet co-expression on day 5 shown. Line depicts mean ± SE. Each symbol represents one animal. Data from 5–6 experiments. (TIF) [file ppat.1008555.s002.tif]

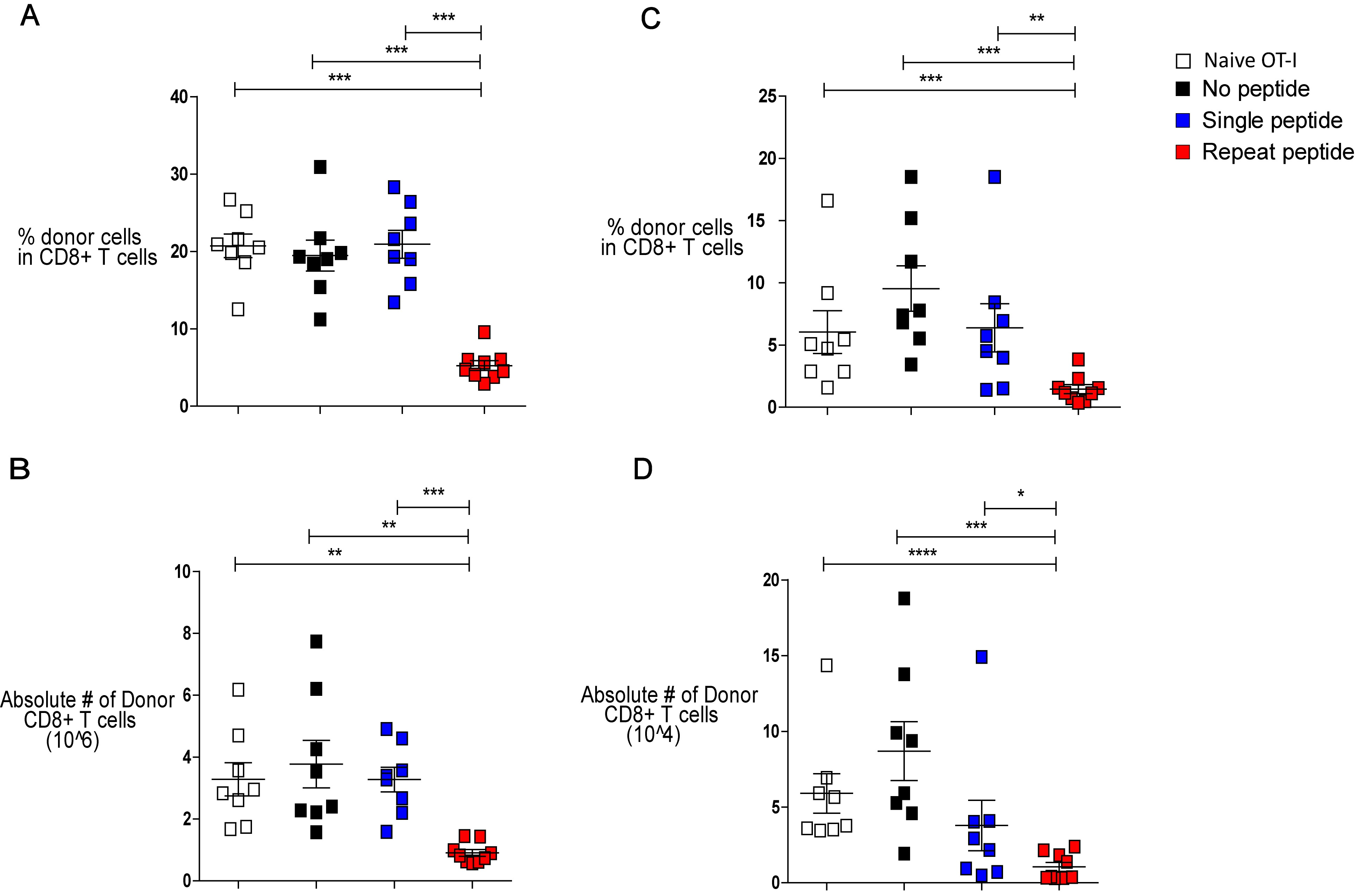

Supplement: S3 Fig — OT-I CD8+ T cells were cultured without peptide stimulation (no peptide), one-time stimulation (single peptide) or daily stimulation (repeat peptide) and sorted on day 5. Live CD8+ T cells were adoptively transferred into wild type mice which were then infected with the Ova(257–264)-expressing influenza virus WSN-OVA. Freshly isolated OT-I CD8+ T cells from a naïve mouse were also transferred (naïve OT-I). Mediastinal lymph nodes (MLN) and spleens were harvested on day 10 post infection. Frequency of donor OT-I CD8+ T cells within spleen (A) and MLN (C) in total CD8+ T cells and the absolute number of donor cells in spleen (B) and MLN (D) are presented. Each symbol represents one animal (n = 8–9) from n = 3 independent experiments. Line depicts mean ± SE. To determine significant differences between the different animal groups, Mann-Whitney U test was used except for data in (A) (ANOVA with Tukey’s post hoc test). *P<0.05, ** P<0.01, ***P<0.001, ****P<0.0001. (TIF) [file ppat.1008555.s003.tif]

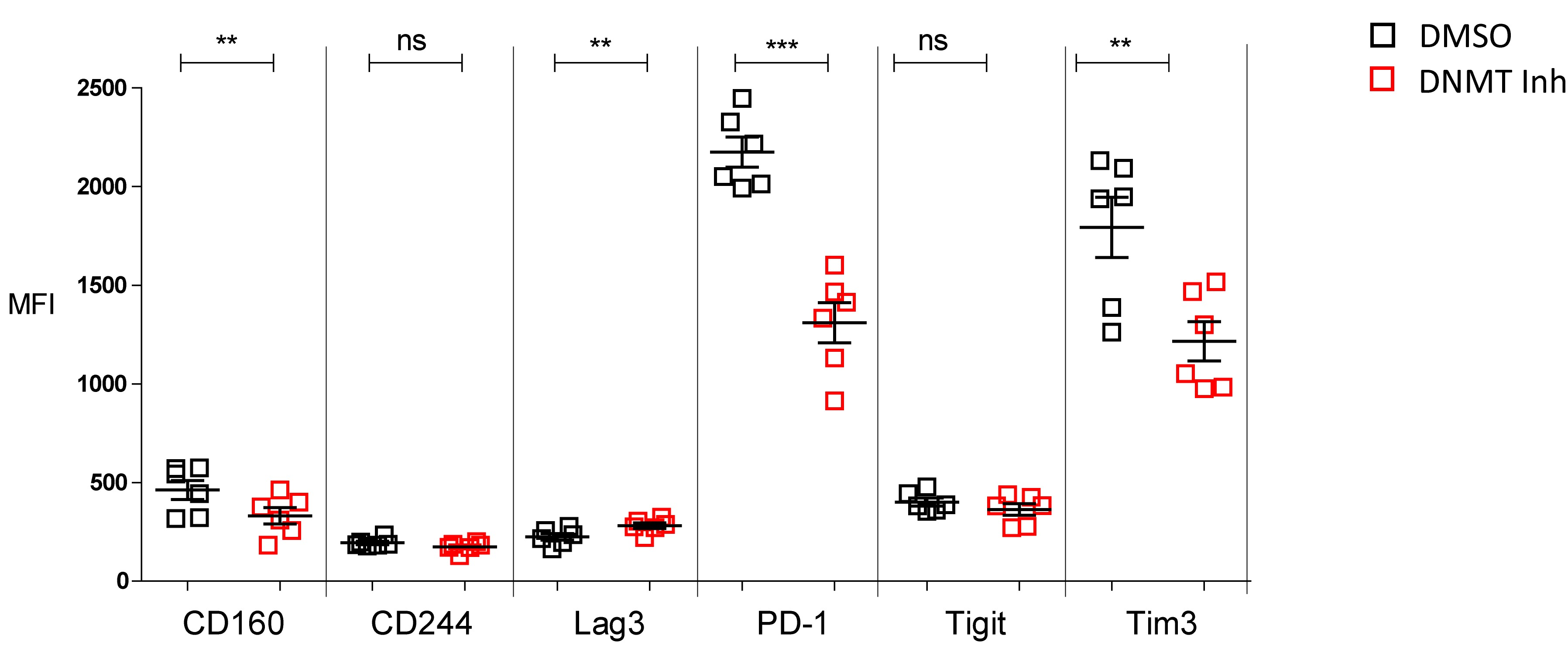

Supplement: S4 Fig — Pooled data showing day 5 inhibitor receptor MFI on repeat stimulated cells in the presence or absence of 20μM DNMT inhibitor. Inhibitor was added during the last 3 days of culture. Data are from n = 6 animals, performed in 3 independent experiments. Line depicts mean ± SE. To determine significant differences between the different treatment, paired t-test was used *P<0.05, ** P<0.01, ***P<0.001. (TIF) [file ppat.1008555.s004.tif]

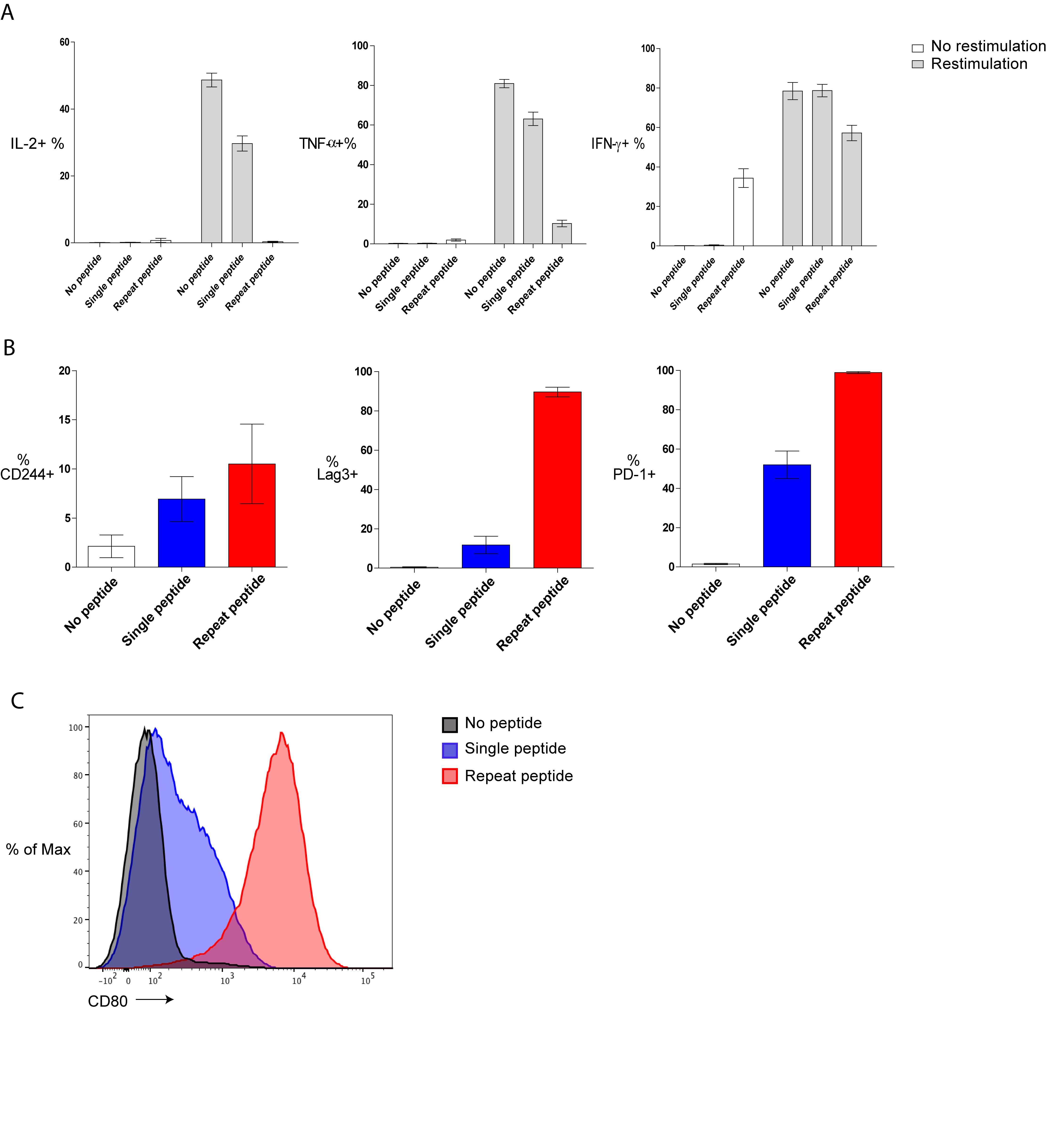

Supplement: S5 Fig — CD8+ T cell exhaustion was induced in the presence of IL-2 (20U/ml) and IL-7/IL-15 (5ng/ml each). Cytokine production after OVA peptide restimulation for 6 hours (A) and inhibitory receptor expression on day 5 (B) are shown. n = 8 animals from 5–6 independent experiments depicted. Representative histogram of CD80 expression on the cells is shown in (C). One of two independent experiments shown (n = 3). (TIF) [file ppat.1008555.s005.tif]
