# Supplementary material for: Rapid in vitro generation of bona fide exhausted CD8+ T cells is accompanied by Tcf7 promotor methylation
Source: PLoS Pathog. 2020 Jun 24;16(6):e1008555. doi: 10.1371/journal.ppat.1008555 (PMC7340326; doi:10.1371/journal.ppat.1008555)
Supplement: S1 Table — Log2 fold change is show for repeat peptide OT-I versus single peptide stimulated OT-I. After 5 day cultures, cells were sorted and RNAseq was performed. Genes with >2 fold change and padj value < 0.05 shown. (PDF) [file ppat.1008555.s007.pdf]

S1 Table

Differentially expressed genes in repeat peptide stimulated T cells determined by RNAseq. Log2 fold change is show for repeat peptide OT-I versus single peptide stimulated OT-I. After 5 day cultures, cells were sorted and RNAseq was performed. Genes with >2 fold change and padj value < 0.05 shown.

Repeat peptide stimulated cells versus single peptide stimulated cells

GeneName	baseMean	log2FoldChange	padj
0610009L18Rik	201.755764	-1.277960455	5.78E-10
0610040F04Rik	157.463514	1.005966933	1.97E-05
0610043K17Rik	246.423422	-1.890880273	1.78E-09
1110004E09Rik	713.97038	1.132302939	9.18E-08
1110032F04Rik	497.463112	1.334317684	1.71E-09
1110034G24Rik	1260.61844	-1.212866741	5.04E-06
1110038B12Rik	1041.92657	1.140144129	3.50E-25
1500015A07Rik	173.053305	-1.025085142	2.97E-08
1600014C10Rik	141.232962	-1.266496621	1.97E-17
1700012B07Rik	148.845126	1.273366292	0.00048402
1700016L21Rik	688.01262	-2.909133086	1.79E-13
1700019D03Rik	127.658139	1.207694085	2.28E-11
1700025G04Rik	5549.57115	-1.992945691	1.19E-16
1700029H14Rik	213.319125	-2.077799804	1.09E-12
1700061G19Rik	298.832636	-1.338453548	4.87E-15
1700093J21Rik	144.711729	-1.394182318	3.47E-10
1700097N02Rik	260.085145	-1.185653388	6.79E-07
1700113A16Rik	336.907184	-1.107496337	4.11E-09
1810006J02Rik	107.815508	-1.024822621	0.0017972
1810055G02Rik	362.254473	1.051476986	2.80E-06
1810062O18Rik	144.742803	-1.222906118	5.40E-07
2010016I18Rik	246.576404	-1.416294018	0.00014416
2310002F09Rik	281.517829	-1.696794234	1.43E-06
2310015A10Rik	790.314245	-1.494037491	4.21E-05
2410004P03Rik	102.689452	1.046458183	3.26E-05
2500002B13Rik	215.217327	1.043199156	1.19E-05
2610027K06Rik	106.043246	-1.09139961	1.93E-09
2610035D17Rik	5450.18857	-1.553342416	2.02E-18
2610037D02Rik	1134.25854	-1.084916316	0.02077512
2810001G20Rik	474.674802	-2.164396653	2.49E-23
2810025M15Rik	113.95226	1.505864525	1.22E-07
2810429I04Rik	142.110463	1.661204133	2.27E-10
2810474O19Rik	7936.50803	-1.887798969	2.60E-31
2900026A02Rik	458.233558	2.443320541	2.38E-26
3110009E18Rik	843.4786	-1.392313753	0.00014612
3110056K07Rik	1235.08351	-1.038234307	1.19E-06
3632454L22Rik	261.47648	-1.763420197	1.79E-10
4632404H12Rik	456.360162	-1.356728656	0.00039228
4632427E13Rik	647.671545	-1.087590612	0.00690033
4732487G21Rik	448.448723	-1.194466783	1.22E-14
4831440E17Rik	104.813391	-1.056140851	1.29E-06
4833422C13Rik	495.782719	-2.243542281	1.06E-13
4930417H01Rik	140.901783	-1.535943467	4.21E-10
4930431P19Rik	108.956427	-1.044264457	4.31E-06
4930452B06Rik	180.134784	2.03889756	9.40E-21
4930481A15Rik	563.544076	-1.76909401	1.50E-07
4930503L19Rik	1140.69694	1.017402433	9.21E-07
4930519F09Rik	571.567524	-1.079212494	0.01873979

4930523C07Rik	7111.78017	-1.266950845	1.75E-16
4930539E08Rik	181.975224	1.013466282	0.000217
4930562C15Rik	289.857266	-1.210972181	1.64E-16
4930581F22Rik	546.072963	-1.00856743	0.00013811
4930595D18Rik	185.268467	-1.227033709	8.17E-07
4930599N23Rik	242.191857	-1.242924152	4.56E-10
4931403E22Rik	210.427455	-1.362198561	1.96E-08
4931406G06Rik	120.565936	1.491473677	1.04E-06
4933406I18Rik	4224.68286	-1.4319444	1.68E-05
4933440M02Rik	1121.16328	-5.019846897	2.65E-180
5031434O11Rik	249.00109	-1.309540211	1.95E-17
5430427O19Rik	415.306351	-1.132245741	0.0037465
5430431A17Rik	96.1828446	-1.280930112	4.45E-07
5730508B09Rik	3328.92212	-2.549236371	1.89E-26
5830411N06Rik	1390.92189	-3.530640426	8.52E-21
5930435M05Rik	78.1306874	-1.088296906	9.07E-05
6430571L13Rik	144.319576	1.401224378	3.00E-08
9330104G04Rik	155.48361	-1.247822332	1.95E-09
9330136K24Rik	301.267476	1.073266597	1.60E-11
9330175E14Rik	728.519759	-2.01437599	8.93E-12
9330198N18Rik	239.59375	-1.079897164	0.00018411
9430015G10Rik	1007.01695	1.082508547	7.32E-11
A230072E10Rik	304.54998	-1.366868633	2.97E-05
A330040F15Rik	359.522083	-1.263278846	0.00026245
A430046D13Rik	371.471542	-1.26546001	2.49E-09
A430078G23Rik	5454.74589	-2.438013784	8.90E-15
A530021J07Rik	105.601109	-1.552433655	3.43E-08
A530072M11Rik	744.690283	-1.344114564	1.17E-05
A630014C17Rik	300.15033	-1.825075226	8.68E-13
A630023P12Rik	123.070368	-1.227348929	0.00020506
A730081D07Rik	526.670774	-1.762478289	3.66E-06
A930002I21Rik	381.601297	-3.390673598	2.34E-41
A930005H10Rik	463.216403	-2.213257447	6.26E-17
A930007I19Rik	283.115374	-1.026655303	0.0001179
AA467197	1635.75245	-3.826983179	4.10E-28
Aaas	2122.58274	1.141188114	1.77E-13
Aaed1	1176.27732	-1.078815803	7.34E-12
Aars	4166.52487	1.781077376	3.42E-20
AB124611	1936.77005	-1.793949354	1.31E-36
Abca1	637.596717	-1.350922444	4.33E-05
Abcb11	81.0543881	-1.05737461	0.00023583
Abcb1a	819.99539	-1.66475352	7.77E-08
Abcb4	173.239607	1.227029937	1.01E-09
Abcc10	582.293293	-1.044699494	5.49E-06
Abcd3	2096.09023	1.978137615	5.48E-26
Abcf2	3080.86598	1.20306454	3.09E-10
Abcg1	3555.09412	-2.364192707	1.69E-98
Abcg3	729.600987	-1.017699875	6.14E-05
Abhd15	585.059232	-1.693971821	3.13E-23
Abhd4	531.931195	2.059130891	3.04E-14

Abi2	1781.5438	2.01123681	2.29E-31
Abi3	147.445927	-1.49264521	1.18E-15
Ablim2	254.587863	-1.509432353	1.77E-09
Abr	4267.51624	-1.064799925	6.39E-21
AC108401.3	251.298497	-1.341232258	2.95E-11
AC124680.1	668.634527	-2.496536894	0.00206902
AC133498.1	567.020266	3.818688979	3.93E-29
AC133650.3	104.906227	-1.201123133	2.79E-06
AC153495.1	1124.18256	-4.619726223	1.66E-35
AC153862.2	1819.05338	-1.788457653	2.21E-16
AC153899.1	191.511171	-1.092005578	4.12E-11
AC154682.1	316.141552	-1.134812766	2.50E-06
AC156795.1	340.470128	1.125941811	0.0001512
AC157822.3	682.505874	-1.791452905	2.87E-06
AC158388.3	355.992927	-2.32517399	0.00040448
AC160405.2	220.769412	-1.0626912	1.89E-11
AC163032.1	448.311304	-1.421147506	6.07E-16
AC163354.1	218.521833	1.620938898	3.79E-05
AC163623.3	85.9125347	-1.00797423	5.56E-05
AC164426.2	727.476841	-1.112476949	4.13E-07
AC167978.1	118.390689	-1.192513239	2.31E-05
Acad10	504.000352	-1.298254147	2.92E-05
Acot2	429.671182	-1.061043245	3.36E-09
Acot7	3380.92754	1.721215533	2.33E-20
Acpp	564.886907	-3.199041339	2.60E-20
Acsbg1	1031.01781	-1.259837369	6.72E-08
Acsf2	1804.82861	-1.356456467	4.63E-17
Acsl3	620.226146	1.695092751	7.55E-22
Acsl6	296.549161	3.032225858	1.81E-29
Acss2	938.536656	-1.725505634	2.16E-24
Actg1	29511.8181	1.016647686	0.00249174
Actn1	6808.44206	-2.330475557	1.81E-36
Actn2	383.023003	-1.1475751	7.35E-05
Acvr1	359.871775	2.481499639	5.21E-33
Acvr1b	1183.42091	-1.037791711	1.19E-07
Acvrl1	311.684069	-2.731477346	3.10E-18
Adam19	5915.68221	-1.166504949	1.75E-12
Adam8	247.379592	1.055781326	5.90E-05
Adamts14	732.355192	3.559494119	4.37E-44
Adamts6	7759.45561	2.007284444	2.28E-08
Adamtsl4	263.12519	-1.243968481	0.00025845
Adap1	1447.48992	1.443769302	7.29E-08
Adarb1	678.758628	2.768935442	4.08E-51
Adarb2	168.397407	1.532075752	2.98E-06
Add3	12553.5344	-1.275378179	5.33E-12
Adgrb2	142.785251	-1.352388997	1.08E-05
Adgrd1	137.77894	-1.936858969	7.25E-22
Adgrg3	110.873364	-1.396308382	1.45E-05
Adgrg5	2222.61055	-2.055069518	2.45E-31
Adh4	129.541352	1.407011257	1.07E-08

Adora2b	232.032376	2.186515051	9.46E-31
Adrb2	362.215285	-2.261390764	5.35E-20
Adssl1	305.77701	1.261829392	1.25E-05
Aebp1	215.592036	1.34984828	1.01E-09
Afp	106.567809	-1.152709793	6.42E-09
Agtrap	1014.54963	-1.009373808	8.54E-08
Ahr	179.129958	-1.200676269	4.79E-07
Ahrr	191.506962	-1.747912969	1.14E-09
AI506816	8027.93668	1.164599428	1.20E-10
AI847159	220.874664	-1.279893861	1.18E-12
Aim2	5118.79966	-1.272376878	0.00229188
Ajuba	119.071312	1.414915672	5.22E-08
Ak2	3128.62175	1.235400374	3.90E-08
Ak4	971.069788	2.358254125	1.73E-07
Ak6	588.881724	1.24779094	6.87E-17
Ak8	115.293273	-1.254521341	9.14E-12
Akap1	895.467557	1.042747944	2.59E-10
Akap5	258.280046	-1.023142266	1.70E-05
Akr1c13	282.879243	-1.933070654	3.99E-09
Akr1e1	476.230487	1.403923218	2.87E-15
AL513022.1	97.3965376	1.388232502	2.59E-13
AL589871.2	111.280631	1.649725259	7.73E-06
Alad	616.026169	1.411769877	1.40E-13
Alcam	2863.85921	2.599058319	7.73E-18
Aldh18a1	4157.63953	1.236105524	4.80E-13
Aldh1l2	120.992697	1.622363172	2.26E-08
Aldh3b1	232.075721	-1.607973083	3.07E-09
Aldh7a1	612.063412	1.211050698	7.14E-07
Aldoc	733.676852	-1.731442684	7.12E-05
Alg8	1365.12731	1.025526479	1.72E-08
Alpk2	736.810713	1.154178953	0.00022896
Alyref	4726.83089	1.309919911	2.77E-06
Amdhd1	129.084992	-1.385708246	9.57E-11
Amigo2	303.299136	-1.716326988	4.56E-08
Ampd1	289.101641	-1.495624795	1.82E-10
Ampd3	157.083628	-1.091569779	1.62E-06
Amy1	567.596112	-1.446080145	1.06E-05
Angptl4	421.472225	1.163019912	3.07E-06
Ank	3136.59586	-1.503704558	9.50E-17
Ank3	96.4389354	1.274556721	5.57E-08
Ankmy1	110.197651	-1.216209193	3.76E-12
Ankrd13b	889.439776	2.038893709	5.67E-21
Ankrd33b	235.150847	3.063050352	1.17E-26
Ankrd52	3196.00001	1.267459379	4.11E-11
Anxa2	6945.13191	1.351010286	0.0006341
Ap3m2	1006.21162	-1.70288873	9.72E-19
Apbb1ip	9806.51678	-1.332385047	4.53E-08
Apc2	193.479228	-1.086750585	4.59E-09
Apol7e	2005.24972	-2.894183834	6.41E-51
Aqp10-ps	97.6838487	-1.037218389	0.00067728

Aqp3	381.626413	-2.08721889	2.50E-13
Aqp9	639.351341	-3.831214902	3.84E-30
Arc	221.26135	1.004772252	0.00080866
Arfgef3	408.468939	2.705074724	7.21E-13
Arhgap15	73352.4505	-1.335057376	0.00013741
Arhgap21	2252.51046	1.61277153	1.76E-13
Arhgap23	146.302521	1.241457553	5.09E-12
Arhgap24	267.017843	2.515266873	4.86E-17
Arhgap25	5362.15474	-1.238642922	5.48E-09
Arhgap4	4050.23913	-1.115585119	1.83E-14
Arhgap45	17994.4464	-1.242426697	9.29E-11
Arhgap6	97.7425403	-1.192436002	0.00163306
Arhgdig	284.425104	1.753135435	5.36E-11
Arhgef11	791.715658	-1.297403251	5.17E-12
Arhgef18	9968.83645	-2.428792683	1.13E-26
Arhgef25	198.702717	2.281147809	5.08E-20
Arhgef3	15125.2807	-1.003921233	1.35E-06
Arhgef40	184.677077	1.307778182	8.91E-13
Arhgef5	141.839634	1.292784616	4.93E-07
Arhgef9	993.153353	3.096787204	4.36E-18
Arl14ep	2200.91236	1.820945471	1.73E-44
Arl4c	11138.3176	-3.886515032	2.20E-64
Arl5c	2434.19655	-2.697440738	1.90E-56
Arl6	254.319739	1.667495508	4.06E-21
Armc3	176.105426	-1.728074272	3.24E-20
Armc7	1931.61985	-1.271729993	3.84E-24
Arntl2	125.887408	1.667172867	8.88E-10
Arrdc4	109.749829	-1.131815026	0.0001307
Art2a-ps	117.701603	-1.172761186	2.29E-05
Art2b	1166.79715	-4.503040704	3.99E-42
Art4	257.960029	-1.443499872	1.09E-14
Arv1	892.065727	-1.056161551	2.69E-05
As3mt	170.67135	1.534162857	1.61E-10
Asap1	16716.1526	-1.987900636	6.89E-15
Asb13	1022.11699	-1.06667682	1.25E-05
Asns	1434.50271	3.601494006	2.71E-42
Asph	701.185047	2.876210713	1.45E-28
Atad5	3514.40908	1.037123864	1.73E-07
Atcay	218.148339	1.333421675	2.24E-10
Atf3	929.957202	1.627412573	0.00760761
Atf4	4560.49137	1.583283697	7.01E-10
Atg10	3212.4646	-1.133941615	0.00102798
Atg9b	392.646276	1.091021091	0.00012626
Atp10d	3009.8506	-1.943097269	3.91E-12
Atp13a2	674.610172	1.192487489	5.41E-07
Atp1b1	1154.38132	-2.171769141	1.31E-32
Atp1b3	17285.7267	-1.334821874	6.36E-29
Atp2a2	7754.79201	1.115960945	4.70E-15
Atp5b	23324.9475	1.314193225	5.64E-07
Atp5g3	4003.56624	1.033097994	8.65E-06

Atp6v0a1	156.075065	1.857207298	3.83E-23
Atp7b	200.021286	1.344524537	0.00082071
Atp8a1	6816.93803	-1.429048558	2.51E-07
Atp8a2	261.087595	-1.128504967	8.56E-06
AU019990	179.704721	-2.009098471	1.70E-13
AU021092	79.5445862	-1.151257389	1.35E-05
Aunip	628.662196	1.60740893	1.73E-16
Aurka	1444.67827	1.30193908	1.16E-06
Aurkb	2359.47941	1.451135213	7.17E-07
Auts2	1876.92857	-2.621848058	1.85E-13
Axdnd1	164.28224	-1.306540453	8.81E-11
Axin2	1061.43613	-1.886131764	1.38E-24
Axl	613.113821	2.335407608	2.60E-16
B230217O12Rik	590.579298	-1.099140499	0.00026772
B3galt4	285.045327	-1.354911152	4.36E-09
B430306N03Rik	189.713821	-1.325933916	1.21E-13
B4galt1	10341.9175	-1.184531084	1.73E-20
B4galt7	1156.40456	-1.174383353	4.65E-14
Bace2	95.7993722	1.371784829	4.61E-08
Bach1	1339.38644	-1.758775373	2.80E-64
Bag2	408.700788	1.039447625	3.99E-10
Bag3	357.378599	1.013259914	0.00020074
Baiap3	952.176222	-3.480545176	2.51E-62
Bambi-ps1	344.760748	-1.812004273	1.94E-08
Bard1	4458.78859	1.18425848	1.19E-05
Basp1	542.020168	2.644653739	5.15E-13
Batf3	738.668081	4.309664604	3.78E-65
BB557941	118.667207	1.191233509	9.39E-06
Bbof1	270.456017	-1.712033047	8.99E-12
Bbs5	116.203468	1.430002072	1.72E-13
BC030867	885.1412	1.79410761	7.39E-13
BC035044	764.586401	1.884891951	7.56E-07
BC043934	371.697314	-2.310141487	1.44E-34
BC055324	1220.78799	1.21967799	2.50E-07
BC147527	614.876449	-2.155628918	7.88E-28
Bcam	94.0617281	1.078489578	0.00026006
Bcar1	114.197052	1.241310033	0.00134849
Bcas3	7086.43151	-1.048895366	0.00026899
Bcat1	2888.37262	3.361287654	6.77E-90
Bcl11b	13953.6961	-1.351996477	1.22E-08
Bcl3	580.903939	-1.323685602	4.92E-06
Bcl6	423.377001	-1.32897167	1.82E-14
Bcl7c	772.401971	1.092998337	2.44E-05
Bcl9l	3892.90686	-1.958818815	7.02E-37
Bcr	2673.42525	1.024794154	1.56E-08
BE692007	700.426465	-1.251336355	0.00035883
Bhlha15	182.439489	-1.272123566	3.66E-12
Bhlhe40	5994.87963	2.392200128	1.55E-05
Bicd1	195.25731	2.245717343	5.17E-22
Bin1	2925.75155	-1.047781539	4.66E-10

Bin2	8902.6004	-1.320582726	8.42E-14
Blm	3607.45364	1.390531446	1.18E-12
Blvrb	207.556854	-1.023451317	6.54E-07
Bmpr2	894.154906	1.183168629	1.24E-16
Bnip3	733.304101	1.33339368	3.52E-05
Bnipl	148.695603	-1.175553656	1.10E-06
Boc	93.884334	1.432634229	6.73E-08
Boll	818.333648	-1.359350637	2.25E-05
Borcs8	1617.43847	-1.184950406	7.32E-11
Brca1	3778.604	1.19378118	8.36E-07
Brca2	2326.54882	1.241275286	2.22E-09
Bsg	6764.88929	1.27551364	4.94E-06
Bsn	248.720927	-1.394819055	5.17E-16
Bspry	507.644484	2.666788303	7.94E-20
Btbd11	4872.5367	-1.24971795	1.59E-05
Btbd8	630.864147	1.099035634	8.32E-07
Btg1	12895.4306	-1.510187898	1.43E-13
Btnl7-ps	148.357704	-1.15210006	2.90E-08
Bub1	2500.63584	1.419750355	5.90E-07
Bub1b	4179.26491	1.241494303	4.31E-06
C030029H02Rik	1011.13805	1.084086504	0.00047606
C030034L19Rik	146.278602	-1.932494476	1.39E-09
C130036L24Rik	443.908097	-1.592212129	9.84E-07
C1qbp	3433.78555	1.319622055	1.21E-09
C1qtnf12	298.891717	1.044868703	1.45E-05
C1qtnf6	315.803858	1.126863441	0.00087367
C230024C17Rik	98.9783709	1.034136905	2.23E-09
C230037L18Rik	156.469452	-1.034845432	0.00308778
C530008M17Rik	215.015453	-1.228549533	2.06E-06
C77080	197.745129	1.224532545	7.68E-09
Cacna1c	152.23957	1.4220268	3.94E-09
Cacna1s	244.879686	-2.445638762	0.00322676
Cacna2d4	617.178838	-3.003942559	1.92E-77
Cacnb1	432.333103	-1.717326138	2.91E-13
Cacnb3	222.554802	-1.689017592	6.65E-11
Cad	6696.52929	1.47573189	7.53E-09
Calcoco1	972.038274	-1.66499896	4.86E-15
Calml4	199.062644	-1.107102998	2.30E-11
Calr	13447.2712	1.035607449	0.00090161
Camk1d	16103.2617	-1.14087951	4.58E-05
Camkk1	210.894373	-1.021584905	7.24E-08
Canx	12814.5777	1.059355472	4.88E-08
Capg	2577.47926	2.275609454	6.73E-16
Capn3	325.442792	-1.296728613	1.17E-20
Capsl	198.748165	1.300025231	1.87E-07
Card10	107.810381	1.085678398	8.95E-07
Card11	8150.12354	-1.271371377	5.48E-08
Card14	335.71114	-2.581319985	7.37E-30
Card6	2045.18306	-1.751129061	6.02E-16
Carns1	625.01004	-1.942216837	5.95E-08

Cars	2331.68436	2.295351072	3.93E-29
Casp3	4852.21071	1.854007432	1.96E-13
Casz1	617.184283	-1.783417973	1.31E-22
Cavin3	96.9556822	1.502372779	2.40E-06
Cbarp	506.354604	1.067043427	0.00025734
Cblb	43637.3303	1.262285179	1.35E-06
Cbx5	10885.3121	1.196897601	2.61E-08
Cbx7	3074.46469	-1.38705351	1.25E-08
Cby1	324.232079	1.21757647	7.95E-09
Ccdc112	154.561017	1.425856622	1.38E-14
Ccdc124	1014.55235	1.047136369	0.00013732
Ccdc14	635.007481	1.227795812	9.41E-08
Ccdc141	726.422216	1.653206481	5.87E-11
Ccdc152	206.903946	-1.294336547	7.69E-12
Ccdc162	420.3682	-1.107495704	5.77E-07
Ccdc171	2011.50698	-1.294302658	8.65E-05
Ccdc18	713.163056	1.017100721	0.00016197
Ccdc184	133.48658	1.953179357	5.81E-11
Ccdc30	355.932956	-1.17562072	1.41E-07
Ccdc50	6797.35823	1.349864485	2.65E-20
Ccdc57	485.063841	2.062867471	4.37E-18
Ccdc58	1679.16496	1.018033804	7.49E-07
Ccdc88a	312.211798	1.567333688	1.25E-20
Ccl3	783.77295	2.783265922	4.07E-07
Ccl4	473.044957	1.661063202	0.00011354
Ccl5	783.962418	-2.085578964	2.61E-11
Ccm2	6243.98465	-1.191265885	1.77E-06
Ccna2	4028.49398	1.163722167	0.00039823
Ccnb1	2465.09806	1.84893288	2.13E-08
Ccnd1	133.420293	-1.050629396	1.20E-11
Ccnd3	14168.5133	-1.310273386	9.60E-08
Ccp110	1294.49113	1.49672603	1.81E-17
Ccr2	323.872772	-1.665451804	0.00430345
Ccr4	419.483029	2.165146649	7.82E-12
Ccr5	870.661505	-1.899436835	0.00150186
Ccr6	189.850477	2.749570845	7.38E-27
Ccr9	161.699348	-1.02836866	0.00027022
Ccser1	174.183037	1.670078787	8.82E-13
Cct3	7121.8962	1.203428698	1.47E-12
Cct6a	8555.7231	1.371055106	5.46E-11
Cct7	8242.52566	1.217466332	1.40E-09
Cct8	9087.63959	1.064726523	5.91E-08
Cd163l1	796.906964	-2.789119773	5.35E-20
Cd1d1	377.267099	-1.79370775	1.55E-18
Cd200	1089.41773	2.355366601	1.57E-29
Cd200r4	231.166888	-2.105673643	1.18E-16
Cd226	9496.21641	-3.259855945	3.83E-14
Cd247	17302.5968	-1.244270679	3.48E-09
Cd24a	196.112541	1.244301826	7.98E-06
Cd37	2409.9843	-1.086981797	1.08E-11

Cd44	7824.54986	2.493437571	1.85E-16
Cd46	595.192621	-1.183043221	0.00064529
Cd48	7318.06628	-1.345135974	1.32E-36
Cd52	7316.65663	-1.112779647	0.00016174
Cd55	1591.1387	-3.161958067	1.95E-34
Cd72	345.693651	-1.290447215	5.36E-16
Cd79a	145.856436	-1.29241578	3.14E-07
Cd79b	163.715979	-1.440614408	3.98E-06
Cd80	2590.50394	3.691775214	1.58E-65
Cd81	499.49844	3.689005351	4.73E-85
Cd83	344.333933	2.81546128	8.42E-26
Cd84	3936.23734	-1.078773061	7.27E-09
Cd8b1	19630.6589	-1.485155095	1.58E-20
Cd96	8800.87229	-1.085630519	3.06E-07
Cdc20	2397.71782	1.319469753	0.00014941
Cdc25a	2130.06191	1.440146816	2.75E-19
Cdc25c	679.309868	1.247071131	3.07E-06
Cdc34	2344.07825	1.116301987	4.04E-05
Cdc42bpa	256.80737	1.850397693	8.26E-15
Cdc42bpb	104.351282	1.17619662	1.60E-07
Cdc42ep3	629.717485	-1.136473622	2.61E-10
Cdc42se2	6601.15379	-1.193616699	4.21E-17
Cdc45	2639.14223	1.508656213	2.97E-10
Cdc6	2332.06412	1.774669736	2.48E-10
Cdca2	2671.55791	1.201838875	5.60E-07
Cdca3	1998.285	1.011820721	0.00121887
Cdca5	1336.63989	1.337826583	1.59E-05
Cdca7	2363.23505	1.349263236	8.76E-09
Cdca8	2274.01765	1.385778744	4.04E-06
Cdh1	104.57761	-1.871055394	3.20E-06
Cdh23	1807.45974	-2.146694662	5.59E-44
Cdk1	3682.23403	1.101094138	0.00030431
Cdk2	1437.28217	1.09641365	1.07E-09
Cdk2ap1	1380.30442	1.52844625	3.28E-18
Cdk4	5500.68584	1.077508532	2.08E-05
Cdkn1a	1071.03207	3.646493903	1.21E-21
Cdkn1b	2652.17894	-1.159440419	5.51E-10
Cdr2	486.849992	1.083509594	4.24E-16
Cdr2l	145.978083	-1.212784052	2.08E-07
Cdv3	6964.50746	1.211075478	2.97E-10
Ceacam15	287.634118	-1.697279137	7.37E-06
Ceacam-ps1	408.788281	-1.394143379	1.66E-13
Cenpa	4318.20237	1.729662738	3.57E-13
Cenpe	5469.67695	1.27919781	7.01E-06
Cenph	874.789935	1.472566797	2.41E-11
Cenpi	1631.22347	1.363257819	4.15E-06
Cenpk	891.265698	1.243123373	0.00037919
Cenpp	2767.86415	1.620916358	1.41E-07
Cenpt	763.993925	1.004491487	5.77E-08
Cenpu	626.917093	1.07007501	1.42E-07

Cenpv	436.636264	1.310900145	3.24E-17
Cep290	460.30194	1.827059278	1.32E-15
Cep55	1717.76677	1.382801993	2.34E-08
Cep85l	908.190822	1.677073539	2.17E-12
Cep97	4169.88445	-1.217896753	2.74E-08
Cercam	955.494175	-1.133228023	1.09E-08
Cers6	5033.37753	1.404119905	7.16E-10
Cfap100	245.767172	-1.234760946	1.51E-10
Cfap54	801.597975	2.600289048	7.05E-37
Cfap74	223.639298	-1.32725912	2.24E-05
Chac1	162.921237	2.388693507	2.80E-18
Chaf1a	2910.51993	1.676263277	6.15E-13
Chchd10	573.069835	2.546543476	3.41E-22
Chchd4	639.732462	1.083027807	1.13E-21
Chd3	10093.6848	-2.122720424	2.99E-23
Chd5	185.376773	-1.241095996	1.06E-11
Chek1	1204.49902	1.460965	3.71E-08
Chil5	490.988668	-1.527768136	0.00020977
Chl1	1074.25315	5.274163274	2.32E-81
Chn2	476.29021	2.268935081	6.19E-07
Chrm4	128.865615	1.088760354	0.00011869
Chrna2	241.066324	-1.001195524	0.00010795
Chrna9	228.850664	-1.154028377	7.15E-06
Chrnb1	335.039232	-1.562414732	1.09E-10
Chst10	1081.63371	-1.359237642	4.66E-48
Chst2	626.729778	2.182571593	4.05E-16
Chst3	105.703385	1.562146405	1.04E-07
Chtf18	883.521037	1.368912293	4.72E-07
Cinp	1337.13224	1.442288295	1.99E-29
Cit	5325.64471	1.172458196	3.96E-06
Ckap4	130.87389	1.06013081	0.00064118
Ckb	167.54996	1.385780407	3.69E-06
Cks1b	1346.9777	1.34626614	5.92E-05
Clcf1	1842.56086	-1.442162163	6.13E-06
Clec2d	6158.33604	-1.391656537	6.53E-11
Clec2i	735.733398	-1.202228959	0.00016474
Clic4	4692.53581	1.356274072	1.50E-10
Clip2	399.295554	1.430526496	1.78E-14
Clip3	113.813242	1.25542849	4.83E-05
Clspn	3974.62115	1.136358133	4.88E-06
Cluh	3820.6117	1.256203082	4.31E-11
Cmah	13140.6816	-2.157079294	5.44E-21
Cnga1	269.896599	-1.867790871	3.98E-16
Cnih3	147.921243	2.087792948	9.64E-19
Cnp	4837.30651	-1.271202382	9.47E-07
Cnr2	873.728852	-2.369246008	6.38E-32
Cobll1	2498.09647	1.88007347	4.98E-12
Col20a1	340.795724	-1.397176085	1.06E-08
Col23a1	367.08163	-1.522788482	4.67E-23
Comt	1251.8547	1.057698069	1.98E-14

Coq7	336.709325	1.05602284	5.54E-08
Coq8a	507.485541	-1.132917746	4.65E-12
Corin	112.929842	-1.185595772	2.31E-06
Cpd	1122.16966	2.076385496	3.73E-25
Cpq	1570.48694	-1.494051009	0.0230701
Cracr2a	6397.84069	-1.794951344	1.01E-12
Creb3l2	1446.9975	1.450582307	1.31E-20
Crebl2	916.239018	-1.359556631	2.57E-09
Crebrf	3826.10399	-1.920166432	6.89E-23
Creld2	793.343853	1.352252892	1.75E-06
Crip1	5822.31589	-1.051995674	0.00520029
Crlf3	8193.12518	-1.170863921	6.33E-09
Crybg3	1127.54319	1.147445216	1.45E-07
Crygn	156.602734	-1.214414123	3.86E-10
Csf1	502.900786	3.151215218	6.35E-19
Csf2	102.298928	1.349665021	3.84E-05
Cst3	1442.95179	1.614196733	9.61E-07
Cstad	369.013187	-1.35815401	7.45E-05
CT030173.1	399.878018	1.07522207	4.03E-06
Ctc1	1645.65841	1.346441681	1.22E-13
Cth	292.028216	3.416416877	2.63E-27
Ctnna1	2745.73875	1.052539011	8.16E-05
Ctnnd2	892.164444	-2.290276391	3.52E-13
Ctps	2630.57414	1.032266049	1.18E-17
Ctso	1334.68081	-1.336145465	9.22E-09
Ctss	2281.21398	-1.099103924	2.14E-11
Ctsw	4631.52383	-1.480878343	3.13E-13
Ctsz	1631.76403	1.085834702	4.32E-08
Ctxn1	137.014129	-1.148863052	3.01E-09
Cxcr3	933.967977	-3.492581282	1.80E-33
Cxcr5	334.465202	-1.946148939	4.48E-16
Cxxc5	523.232313	1.106675457	9.40E-21
Cyb5a	3615.15176	-1.149510352	8.40E-34
Cyb5r1	502.642873	1.233946721	1.38E-07
Cyc1	2244.79732	1.342449255	1.19E-07
Cycs	2072.59972	1.465535404	5.89E-07
Cyp20a1	937.465992	1.174215496	2.94E-19
Cyp2t4	156.168888	-1.226920316	2.26E-18
Cyp3a13	96.5114614	-1.356646224	1.31E-05
Cyp4f13	911.126971	-1.126786147	0.00649877
Cyp4f17	886.28629	-1.213643874	8.91E-05
Cyp51	5129.50377	1.300081813	1.46E-11
Cysltr2	459.479566	-1.280697615	3.33E-06
Cyth1	17858.0385	-1.447514835	7.66E-09
D630045J12Rik	415.057538	3.244829987	7.52E-51
D830025C05Rik	139.696385	-1.220305654	2.46E-06
Daam2	160.417062	-1.032960996	4.47E-09
Dab2ip	143.41985	1.651985356	3.79E-11
Dach2	229.287819	1.05129225	0.00120883
Dand5	159.840455	-1.440336877	2.89E-12

Dapk1	286.418946	-1.366185355	1.51E-11
Dapk2	1055.66911	-3.375234677	3.08E-44
Dbn1	156.219521	-1.70765483	2.82E-07
Dbp	434.311407	-1.950150621	2.72E-25
Dclk1	501.994156	4.242477349	2.84E-85
Dclk2	1273.52358	1.086751123	2.63E-06
Dclre1a	446.418551	1.260088461	1.82E-21
Dcp1b	970.118738	-1.012835244	4.32E-05
Dctpp1	898.036787	1.316348017	0.00016258
Ddb1	12149.0898	1.066974598	2.04E-07
Ddias	635.191365	1.805628731	1.45E-12
Ddit4	2051.66343	1.051823284	0.00214191
Ddn	127.629423	1.457463715	2.02E-11
Ddx1	4459.86773	1.35605616	6.88E-24
Ddx20	1257.69457	1.070000283	1.17E-10
Ddx39	3827.36667	1.206061685	1.80E-10
Ddx58	2927.0352	-1.621891176	2.05E-31
Ddx60	1285.99215	-2.932583695	4.23E-18
Degs2	229.142104	-1.148292407	4.58E-10
Dennd1c	3159.14035	-1.279023064	3.06E-06
Depdc1b	3487.3412	-1.145048002	5.21E-07
Dgka	14565.3734	-1.473880079	5.55E-20
Dgkg	327.464913	2.450456486	1.47E-15
Dgkh	2980.8002	1.909647712	1.29E-13
Dguok	1287.26449	-1.035479061	3.24E-05
Dhcr24	2520.919	1.085917928	8.86E-05
Dhfr	2174.21132	1.459602589	3.72E-09
Dhrs13	310.110236	1.34115411	2.28E-11
Dhx29	1509.22328	1.210494279	9.42E-24
Diaph3	10954.2705	1.049417576	0.00140323
Dip2a	392.168795	1.284817735	9.35E-16
Dirc2	2937.84383	-2.04162885	1.80E-15
Disc1	917.804574	-1.124194307	3.15E-06
Dlg2	2903.84932	4.759003026	6.00E-82
Dlgap5	3415.53969	1.244482209	8.48E-08
Dmwd	209.244495	2.674657672	3.37E-20
Dmxl2	149.005506	2.12834685	7.76E-15
Dna2	833.02917	1.375403455	4.41E-10
Dnaaf1	138.868786	-1.266379626	6.27E-11
Dnah17	897.080266	-1.742287372	6.26E-09
Dnah8	6649.66539	-2.094438164	1.98E-11
Dnah9	316.683693	-1.083725071	7.40E-09
Dnajb13	601.825764	-1.028701797	7.73E-14
Dnajb5	620.474803	1.173690315	8.13E-11
Dnajb9	759.651172	-1.29952834	1.39E-05
Dnajc12	154.442672	1.287328206	5.62E-08
Dnajc27	706.116142	1.00871787	1.62E-06
Dnal1	208.459513	1.010893782	7.24E-06
Dnm1	157.125095	-1.64845865	0.00012749
Dnm3	193.442533	1.45349257	3.06E-08

Dnmt1	14786.7115	1.120362608	9.19E-07
Dntt	1003.51745	-3.12541911	6.62E-59
Dock2	54792.0722	-1.030425844	3.03E-08
Dock3	119.19422	1.335326146	3.93E-09
Dock8	19702.6262	-1.066530811	5.93E-14
Dpagt1	512.357644	1.035958874	2.67E-10
Dph5	3291.96343	-1.06697846	2.06E-05
Dpp4	5212.70032	-1.772839903	2.10E-20
Drc1	2666.43192	-3.901187495	1.29E-50
Dscc1	617.335572	1.035879415	1.95E-06
Dsn1	900.204967	1.118069713	2.47E-11
Dtl	4471.65508	1.529051282	2.10E-08
Dtx1	777.920057	-4.455203884	1.50E-59
Dtx3l	5886.01106	-1.11102901	1.03E-15
Dus2	981.49594	1.176550385	8.51E-10
Dusp1	1284.39039	-1.252372662	0.03201907
Dusp10	2362.83237	-1.082350457	2.43E-12
Dusp14	373.575151	2.269978555	7.52E-19
Dusp3	203.92816	1.017911985	0.00018701
Dusp4	2281.79618	3.273827825	1.23E-23
Dut	4443.68312	1.482576673	2.43E-10
Dyrk2	3692.83278	-1.69660029	1.26E-20
Dyrk3	312.637492	1.639521611	4.38E-08
Dyrk4	332.457554	-1.691846602	9.40E-21
Dzip1	1142.52138	-2.198191025	2.92E-13
E030030I06Rik	148.716936	-1.436431688	1.94E-07
E130102H24Rik	261.648524	-1.544413517	1.19E-06
E130307A14Rik	2567.32393	-1.023805839	0.00452909
E130308A19Rik	1668.24359	1.568537222	1.09E-12
E230001N04Rik	162.40683	-1.096565931	1.46E-07
E230032D23Rik	166.565656	-1.236018778	2.96E-05
E2f1	1237.07673	1.137712285	2.58E-07
E2f3	5366.08764	1.161637835	1.19E-06
Ebf1	346.564237	3.52785602	7.07E-35
Ebna1bp2	2732.41196	1.203010307	5.82E-10
Echdc2	198.772415	-1.247470668	2.89E-10
Ect2	3316.76141	1.01884965	0.00066339
Eea1	4247.8851	1.900247934	1.16E-14
Eef1g	23167.9281	1.034987592	4.98E-06
Eepd1	139.771199	-1.021429984	4.44E-08
Efcab5	177.320546	1.052807735	5.21E-07
Eftud2	4326.92386	1.139302606	3.30E-08
Egln3	1818.40373	1.548536487	6.21E-05
Egr1	1915.03548	1.70420106	1.01E-09
Egr2	467.059029	1.336693776	9.25E-06
Egr3	1252.07719	1.936473022	4.06E-08
Ehbp1	502.568837	1.384755614	9.77E-08
Ehd2	198.473057	1.941984558	3.07E-10
Ehd3	2112.63639	-1.050237481	1.04E-09
Ehd4	1780.79471	1.817831557	2.01E-12

Eif1ax	2483.14209	1.25670145	1.58E-09
Eif2d	2206.87914	1.029120533	1.12E-17
Eif3b	10170.3388	1.249146377	1.90E-08
Eif3c	12777.3728	1.121079392	5.52E-10
Eif4e	3671.12276	1.016333465	1.93E-11
Eif4ebp1	833.161772	1.922956393	7.08E-14
Eif4g1	16190.6389	1.041076196	2.27E-08
Eif5a	21216.1845	1.221085588	1.87E-05
Eif6	2572.90452	1.098649533	2.96E-05
Elk3	2746.81602	1.132381433	4.70E-05
Ell2	717.506455	2.12803776	1.12E-12
Elovl7	804.25286	-2.971659377	2.48E-30
Emb	12354.526	-1.070668121	7.53E-12
Emc9	345.319568	-1.2709493	1.02E-09
Eme1	738.136608	1.107866554	3.72E-09
Eml1	171.811258	2.171623077	4.51E-21
Emp1	772.549033	1.101889522	0.01515517
Emp3	2382.77603	-1.072418548	3.00E-06
Endod1	1658.67584	1.850722013	1.20E-15
Enkd1	407.424135	1.651409818	4.94E-12
Eno1	36285.0513	1.3406243	0.00035351
Eno2	122.154621	1.464526983	3.20E-07
Eno3	1918.30202	2.763774572	1.38E-51
Enox1	125.867997	1.203596304	1.17E-08
Enpp4	818.766633	-1.346175831	2.26E-09
Enpp5	147.241725	-1.321074161	8.45E-13
Entpd5	3260.12192	-1.424142808	1.25E-28
Eomes	848.191548	-2.009484184	6.12E-10
Epb41l5	182.45683	1.113208869	1.21E-06
Epcam	148.310817	1.134146491	4.64E-06
Epha1	382.382019	-1.718408231	1.06E-19
Ephb2	113.31943	-1.07454524	7.34E-05
Eprs	10446.5689	1.190932061	3.09E-20
Epsti1	16956.8294	-1.533338473	3.57E-13
Erc1	1671.13492	1.43638313	1.45E-12
Ercc6l	1076.0687	1.019436309	0.00066015
Erg	967.644318	1.074704584	1.57E-05
Ergic1	2401.93921	1.236155468	1.79E-16
Ero1l	3382.04627	1.121712091	5.75E-10
Erp27	439.482632	-1.489614073	1.12E-17
Espl1	3009.53671	1.417308364	7.95E-07
Espn	314.860862	-1.034079696	3.93E-06
Esr1	1592.36008	-2.058472891	6.88E-10
Esrp2	307.581537	-1.165529101	8.10E-14
Etfbkmt	1070.68163	-1.786733685	1.97E-16
Etv4	309.737547	2.147459932	3.16E-20
Etv5	284.246857	2.674117726	4.12E-56
Evi5	541.975234	1.12366824	1.54E-06
Exo1	2043.80475	1.702143375	5.02E-12
Exph5	686.936508	4.680736905	3.51E-75

Eya2	1584.02552	-1.874960491	3.78E-08
Ezh2	7904.69017	1.077829888	2.47E-08
F2r	2512.28115	-1.825528659	8.07E-15
F2rl1	775.917681	-1.608113948	7.06E-26
F730043M19Rik	221.369576	1.390317347	1.87E-07
F730311O21Rik	127.746185	-1.733254662	3.00E-09
F830016B08Rik	232.463162	-3.235129461	2.35E-22
Faap24	467.20401	1.142896652	9.95E-09
Fabp5	1068.05813	1.797497724	4.14E-12
Fads2	1329.43147	2.515658544	2.17E-10
Fads3	143.263538	1.03180342	9.96E-05
Fah	265.523893	1.19634261	2.35E-12
Fam102a	9349.60845	-1.250815299	1.22E-12
Fam105a	3302.36881	-1.124065303	7.64E-08
Fam117a	3383.76933	-2.427587612	1.41E-11
Fam129b	316.493707	1.423987296	1.58E-06
Fam129c	150.171583	-1.11125465	2.63E-09
Fam135b	159.364323	-1.106547142	7.70E-07
Fam136a	868.568804	1.067266308	2.51E-08
Fam162a	2858.69302	1.242202813	7.33E-16
Fam169b	13622.6249	-1.208280039	9.25E-07
Fam178b	90.3161747	1.068604229	4.36E-06
Fam186b	96.1507495	-1.200125204	9.39E-06
Fam189b	2178.16849	-1.834186532	4.06E-21
Fam19a3	147.042013	-1.440566556	2.03E-11
Fam20a	2306.61986	2.550565526	4.31E-30
Fam210b	390.275452	-1.242182718	7.54E-11
Fam213a	440.177664	2.615557753	1.34E-56
Fam214a	2941.96505	-1.55117013	4.23E-14
Fam222a	211.124467	-1.350092923	5.10E-11
Fam228a	231.394045	1.439333321	3.05E-12
Fam26f	287.285264	-1.693003195	4.00E-10
Fam49a	2372.71367	-2.013004192	9.28E-14
Fam69a	2695.28748	-1.244152117	4.08E-05
Fam78a	7713.47106	-1.953202735	4.10E-22
Fam78b	285.155831	-1.020367904	0.00011255
Fam81a	153.001374	1.450167804	3.30E-09
Fam83f	443.949497	-2.553474002	1.85E-28
Fam89a	523.255944	-2.135994123	1.49E-13
Fancd2	2307.23796	1.711529386	1.43E-14
Fancf	270.598575	1.065820561	1.67E-07
Fancm	1824.81217	1.187091722	5.03E-12
Farp1	5507.18021	2.270169979	6.20E-31
Fas	830.429418	-1.990689054	7.84E-31
Fasl	764.422303	1.492325685	0.0001212
Faxc	86.8161289	1.026552141	5.62E-08
Fbxl12	859.010108	-1.17753175	4.98E-08
Fbxl22	880.895302	-1.673938695	4.98E-19
Fbxo32	1092.53048	-1.808095605	3.00E-38
Fbxw4	2409.62071	-1.160758324	0.00121237

Fbxw9	205.11405	1.137344019	1.24E-07
Fcgrt	393.590626	-3.316366565	5.83E-59
Fen1	339.275671	1.187767465	3.86E-06
Fgd2	110.635456	-1.009043086	8.14E-05
Fgd3	3741.94236	-1.047192978	2.03E-10
Fgf13	13997.4803	-2.180882961	8.81E-05
Fgfr1	169.253427	-1.761792501	1.02E-19
Fhl3	1209.02273	1.034181828	7.17E-07
Fignl1	2359.50634	1.594854328	1.43E-09
Filip1	366.535082	2.03304867	1.13E-08
Fkbp1a	7326.87775	1.044588958	6.40E-15
Fkbp4	4752.56187	1.242865175	5.33E-06
Flnb	7850.77388	1.051744236	1.45E-09
Fmnl3	1637.72348	1.804299903	1.04E-22
Fmo5	256.147089	-1.177417082	9.88E-12
Fn3k	117.183302	-2.271321915	2.21E-14
Fndc3b	568.180728	1.37028765	7.12E-13
Fnip2	658.691226	2.061877083	6.16E-19
Fntb	3435.41825	-1.307209425	2.47E-07
Fos	1993.11415	-2.058575776	1.94E-06
Fosb	4279.98593	-2.217275892	3.73E-07
Foxp1	54784.4855	-1.126041822	0.0001124
Frat1	564.198303	-1.469048842	1.11E-08
Frat2	690.255562	-2.160565104	3.82E-18
Frmd4b	466.219685	1.063337614	0.00298653
Frmd5	266.568225	1.353370723	1.23E-13
Fry	5738.69941	-2.681253181	1.67E-26
Fsd1l	509.28572	-1.167072675	7.70E-05
Fsd2	232.722243	-1.169249007	9.23E-20
Ftsj3	3551.24154	1.108995719	1.15E-11
Fut4	291.106441	1.994734811	8.89E-11
Fxyd7	151.712329	-1.473305489	2.49E-15
Fyb	5527.74212	-1.326482948	1.37E-08
G530012D18Rik	99.3757681	-1.06090688	2.03E-07
Gab2	702.401525	1.684708199	4.90E-09
Gab3	4727.53214	-2.184718559	1.19E-10
Gabbr1	1877.88877	-1.322624942	9.91E-05
Gabrr2	143.344002	-1.371156354	1.83E-05
Galc	575.732533	1.514918784	1.87E-14
Gale	518.287356	1.260736512	8.25E-08
Galm	259.501775	1.173610054	1.23E-13
Galnt10	3329.58615	-2.169096656	2.44E-34
Galnt3	2011.30174	3.001825973	1.86E-19
Gapdh	44424.3551	1.27399437	0.00080976
Gar1	1364.66873	1.177581334	3.00E-18
Gars	7482.63782	1.078363784	3.90E-15
Gas2	1733.36146	1.976668223	4.65E-14
Gas2l1	105.469564	1.141947837	0.00028216
Gas7	196.030955	-1.631562145	7.12E-09
Gata1	285.45028	-2.853830298	4.65E-24

Gatm	142.962426	1.666442962	5.89E-11
Gatsl3	181.851551	-1.424106688	2.88E-11
Gbp11	546.825255	-2.428872265	3.17E-26
Gbp2	3991.50055	-2.189513276	1.05E-24
Gbp8	2570.4947	-1.834898318	6.18E-12
Gcat	671.630602	1.885416565	2.67E-09
Gcnt1	332.827014	2.807107399	9.41E-26
Gcnt2	105.76327	-1.174702716	3.65E-08
Gcnt4	186.482892	1.515664694	1.20E-07
Gdpd1	155.531336	1.227846498	6.74E-13
Gdpd5	414.589966	1.312844019	3.34E-11
Gemin6	334.70011	1.35986011	6.23E-06
Gen1	1258.59564	1.144373439	1.82E-07
Gfra1	122.244197	1.788150057	5.64E-16
Ggact	1807.6013	-1.028658576	1.07E-05
Ggt1	886.878537	-3.696667408	5.26E-57
Ggt5	204.092081	-1.571558871	1.43E-09
Gimap3	12363.7779	-1.231468025	3.86E-15
Gimap6	9926.40664	-1.148391981	8.53E-14
Gins1	876.705183	1.344780057	1.17E-07
Gipc2	100.976305	1.401310314	9.34E-10
Gja1	99.4049431	1.495749147	3.29E-08
Gldc	303.781304	1.534210083	4.90E-06
Glipr2	7240.74466	-1.491352791	1.61E-13
Glis3	166.613222	-1.482615637	4.55E-10
Gls2	271.561521	1.627858227	1.32E-16
Gm10388	90.6637363	-1.007554097	0.00020074
Gm1043	946.716506	-3.191750126	3.88E-39
Gm10521	241.635153	-1.19280495	5.99E-06
Gm10548	225.550377	-1.028378598	2.64E-06
Gm10640	362.438911	-1.969720105	1.65E-34
Gm10676	129.86328	-1.151327361	1.99E-10
Gm11077	122.703266	-1.622507437	2.04E-06
Gm11131	299.907593	-1.355658049	4.99E-14
Gm11210	477.971917	-1.304697577	4.44E-08
Gm11427	1686.49559	1.084839247	0.00322676
Gm11476	879.616923	-1.196211225	0.00115917
Gm11541	83.7252513	-1.154805482	5.04E-06
Gm11579	227.564234	1.107629433	5.94E-07
Gm11613	680.472954	-2.043608655	2.19E-06
Gm11696	338.263514	-1.397610871	3.76E-16
Gm11725	138.667453	-1.996665368	1.96E-12
Gm12185	2043.51658	-1.844124932	6.13E-27
Gm12216	2576.87573	-2.551785094	9.55E-17
Gm12250	1421.4886	-1.263951656	5.33E-10
Gm12319	285.482229	-1.162111302	7.22E-10
Gm12474	651.712572	-1.76000012	9.47E-09
Gm12703	192.193038	-1.490258036	0.01487712
Gm12764	115.153491	-1.387247485	3.54E-10
Gm128	406.039121	-1.745758765	5.43E-29

Gm13067	105.094121	1.174517628	1.89E-09
Gm13710	323.501938	-1.979250156	1.99E-07
Gm13807	864.974897	-1.261083169	4.31E-07
Gm14025	124.520148	-1.078035552	8.25E-06
Gm14027	135.695551	-1.251923687	0.00015513
Gm14029	334.994185	-1.323552209	1.91E-10
Gm14085	10210.9835	-4.809128428	6.71E-50
Gm14168	174.102804	-1.064283215	7.33E-10
Gm14965	142.746187	-1.933328701	2.12E-18
Gm14966	591.0745	-1.343620302	3.15E-06
Gm15283	221.716176	1.941622574	7.74E-22
Gm15337	213.963167	-1.167582572	4.59E-08
Gm15440	1117.15289	-1.436186834	5.97E-16
Gm15441	167.153208	-1.042775238	0.00052449
Gm15473	182.47453	1.181400139	8.69E-06
Gm156	852.845436	5.144581	1.57E-87
Gm15601	279.698153	-1.005309534	2.12E-08
Gm15674	146.568652	-1.707028179	9.73E-09
Gm15943	148.228183	-1.174347848	1.09E-08
Gm16083	305.678969	-2.182924544	7.05E-15
Gm16086	107.000576	-1.40097641	5.89E-08
Gm16124	324.217499	-1.560867161	3.02E-06
Gm16157	169.788028	-1.086334754	5.95E-06
Gm16201	350.428655	-1.579339614	1.36E-12
Gm16242	671.73353	4.58800195	3.73E-80
Gm16341	173.592806	-1.668869056	3.88E-06
Gm1647	91.6159652	-1.097609356	4.18E-06
Gm16576	582.293519	-1.219129948	5.95E-06
Gm17021	192.975888	-1.010153242	1.88E-07
Gm1720	109.360051	-1.186895724	7.44E-08
Gm17745	187.971768	1.741587345	4.81E-10
Gm18752	122.321483	-1.640083107	3.16E-08
Gm1966	9528.06172	-2.075467701	5.45E-34
Gm19705	383.73796	-2.312865846	1.27E-41
Gm19931	88.3714777	-1.079934737	1.06E-05
Gm20219	129.038082	-1.426965503	8.06E-09
Gm20458	102.747646	-1.164357465	8.51E-07
Gm20489	156.764065	-1.384318235	3.97E-17
Gm20532	163.215782	-1.629800044	4.99E-14
Gm20559	2148.01897	-2.851257244	1.16E-39
Gm20560	83.7280206	-1.299382447	1.27E-05
Gm20661	109.755011	-1.19943841	4.40E-09
Gm21987	229.035272	1.519243232	1.82E-08
Gm22513	3674.15558	-1.622781511	0.00640809
Gm23973	192.6385	-1.114131778	0.00097968
Gm24146	657.610949	-1.448380357	0.00020279
Gm24265	232.461357	-1.124459318	0.01663735
Gm24447	4763.32704	-1.542607303	7.50E-05
Gm25360	253.48753	1.11745154	0.00446312
Gm26035	1263.37814	-1.565491654	5.43E-05

Gm26583	266.396696	-2.746884751	8.52E-37
Gm26759	549.729306	-1.113095762	0.00032081
Gm26771	1175.35152	-1.558740455	1.61E-05
Gm26779	324.350626	-1.083842673	0.00018163
Gm26799	419.905008	-1.686175936	1.64E-22
Gm26809	344.911037	-1.24363695	9.40E-12
Gm26839	307.84654	1.106147715	7.92E-06
Gm26848	741.379413	-1.094474531	7.83E-07
Gm26885	192.800247	-1.262538325	0.00017622
Gm26887	264.378416	-1.009375337	0.02496413
Gm26908	624.331467	-1.626487476	1.38E-15
Gm26981	197.431216	-1.158869199	0.00174004
Gm27008	2259.42591	-1.332826158	3.47E-09
Gm27184	106.394215	-1.208958502	1.90E-07
Gm28035	169.317181	-1.294482578	1.38E-14
Gm28052	190.734137	-1.041027932	1.51E-10
Gm28068	115.971251	-1.10414268	2.64E-05
Gm28802	477.846758	-1.036202694	0.00043918
Gm28935	162.519684	-1.435726234	5.88E-13
Gm28981	113.179189	1.623896107	5.16E-09
Gm29243	182.058848	-1.423896803	4.47E-13
Gm29477	92.2002776	-1.079393092	3.05E-07
Gm29642	204.621304	-1.087147194	7.53E-05
Gm29811	173.94178	-1.176812117	2.02E-07
Gm30214	111.710825	1.751292486	1.02E-12
Gm30275	1279.97777	-1.541142499	3.09E-10
Gm30948	1377.8489	-4.818947023	5.63E-18
Gm32633	654.987124	-2.432784766	2.51E-12
Gm33994	160.272207	-1.411644015	1.16E-05
Gm34086	213.263539	-1.07449535	0.00049797
Gm35037	175.796799	-1.863258277	3.74E-14
Gm35572	102.32096	-1.089273687	3.70E-05
Gm35585	205.441125	-2.02596599	1.25E-16
Gm36551	113.499771	-1.356835183	2.71E-05
Gm36999	145.931318	-1.837089131	3.17E-11
Gm37004	435.919292	1.475712861	5.88E-05
Gm37068	336.044338	-2.875041197	4.21E-11
Gm37169	468.182633	-1.341811369	8.04E-08
Gm37387	607.059668	-2.06497801	7.95E-18
Gm37584	85.9307112	-1.072928827	0.00014647
Gm37699	289.076945	-1.082793407	1.65E-05
Gm38155	137.479802	-1.560134592	1.55E-14
Gm38244	776.008364	-1.961216193	1.59E-05
Gm38392	101.657781	-1.08352894	0.00196859
Gm42141	373.801295	-1.452741623	2.66E-10
Gm42372	211.458042	-1.062992985	2.73E-08
Gm42646	111.569421	-1.040714573	7.12E-07
Gm42809	114.987885	-1.059246581	8.05E-08
Gm43004	105.994855	-1.296375481	2.83E-08
Gm43196	131.162672	-1.593019829	4.05E-09

Gm43302	188.021531	-1.188811471	4.92E-08
Gm43434	2015.29892	-1.347722283	1.29E-05
Gm43551	93.0755301	-1.231798083	2.16E-09
Gm43647	894.465114	-2.662523741	6.93E-25
Gm43698	228.064717	-1.660389623	2.09E-15
Gm43769	273.460803	-1.375276135	5.69E-09
Gm44110	114.1505	-1.014177207	1.52E-08
Gm44141	106.789033	-1.192661314	7.48E-07
Gm44148	138.877493	-1.321650629	9.70E-06
Gm44174	9107.43307	-1.894546387	8.95E-08
Gm44710	631.412913	-1.608298569	0.00022444
Gm44751	172.568868	-1.034759744	8.61E-08
Gm44899	286.408531	-1.171188456	0.00125034
Gm45418	82.0341131	-1.100114641	2.39E-06
Gm45552	328.504595	-2.450906546	8.83E-40
Gm45606	187.227707	-1.196710741	1.42E-06
Gm45837	216.268828	-1.202399687	4.49E-11
Gm4759	4839.59648	-2.258210333	5.25E-31
Gm4841	107.99869	-1.813993902	3.33E-21
Gm4881	272.616744	-1.562948824	1.02E-12
Gm4951	657.649251	-3.817482915	3.31E-26
Gm5432	244.532414	-1.114817619	0.00206354
Gm5511	192.564139	-1.466799757	1.15E-10
Gm5538	128.243619	-2.440343549	3.04E-23
Gm5547	236.612287	-1.426404561	8.73E-15
Gm6034	209.694962	-1.798631514	9.24E-15
Gm6657	98.0874649	-1.151080387	4.78E-07
Gm6904	296.474275	-1.300849428	7.45E-06
Gm7457	122.234368	-1.368602071	1.42E-06
Gm7967	183.826546	-1.33346929	3.00E-06
Gm8013	111.079697	1.016847566	0.00019259
Gm8189	1341.3669	-1.194140638	1.52E-05
Gm9574	98.9308869	-1.292977347	1.72E-09
Gm9725	194.33007	-1.068124198	8.41E-08
Gm9800	555.056356	1.016215998	0.00034773
Gm9889	94.3503569	-1.321735855	4.45E-07
Gmds	7466.76144	1.004579839	0.00305756
Gmnn	1734.62637	1.193174528	3.01E-06
Gna15	436.77569	-1.275009711	5.26E-08
Gnaq	4002.91859	1.292126248	1.19E-06
Gngt2	195.711084	-1.759842439	3.94E-13
Gnl1	1743.63042	1.063331736	1.66E-06
Gnptab	8035.26282	1.601017239	4.83E-31
Golm1	2594.30056	-1.950982328	5.10E-35
Got2	4356.72221	1.064278129	4.76E-07
Gp1ba	200.770357	-1.298404601	8.81E-09
Gpaa1	931.7881	1.24898722	9.72E-07
Gpc5	160.304635	-1.392420057	2.89E-05
Gpd2	5152.95043	2.801395169	7.00E-61
Gpm6b	733.686775	1.835373228	5.88E-10

Gpr137b-ps	453.709864	-1.694735083	5.81E-37
Gpr141	177.081145	1.803817434	5.69E-12
Gpr15	324.276394	-3.479049322	3.70E-27
Gpr162	129.364371	1.822800083	3.23E-17
Gpr55	526.663645	-3.734767415	1.37E-52
Gprc5b	119.747883	-1.601684021	2.09E-09
Gps1	1473.89312	1.100433683	2.72E-09
Gpt2	397.232179	2.848228113	4.99E-14
Gpx3	324.815333	-1.289855061	4.21E-09
Gramd3	14921.0238	-2.894295741	8.25E-49
Gramd4	8990.47809	-1.824250375	2.85E-13
Grap2	14051.6531	-1.513508906	1.22E-18
Grasp	394.815936	-1.048543334	2.04E-08
Grb10	230.578575	3.009891979	5.63E-28
Gria3	631.80228	-1.94845097	3.05E-07
Grin2d	107.074976	-1.007682632	2.75E-07
Grtp1	211.698031	-1.268267707	1.59E-15
Grwd1	1151.89745	1.097762656	1.34E-06
Gsap	2023.04807	-1.202402284	7.23E-06
Gsn	1206.92009	-1.925199522	5.42E-08
Gspt1	4591.43619	1.051472188	1.72E-21
Gstt1	259.151646	2.493279255	1.30E-45
Gtf2f1	2491.57988	1.007154365	1.14E-06
Gtf2h4	638.189989	1.329184835	4.95E-10
Gtf2ird1	1220.11186	1.706428621	3.37E-11
Gtf2ird2	1322.61224	-1.166097171	7.70E-11
Gtse1	1526.78164	1.85546532	1.35E-11
Gucy1a3	4725.14567	2.430746415	1.18E-13
Gucy1b3	597.389086	1.441772767	7.53E-06
Gusb	2248.51038	1.113464216	1.67E-13
Gvin1	1944.72432	-1.141789362	4.23E-05
Gzma	169.313963	-1.639794613	5.86E-05
Gzmb	34911.9027	3.636288092	4.47E-16
Gzmc	896.817857	3.596046039	2.80E-08
H1f0	1079.49748	1.67237144	9.21E-05
H2-Ab1	471.349084	2.132532841	0.00012517
H2-DMa	1112.61805	-1.655698994	1.90E-15
H2-Ob	237.295308	-1.685270151	1.32E-12
H2-Q7	5382.51809	-1.147026259	0.00011252
H3f3aos	162.68111	-1.045630909	0.00062118
Haao	1680.31923	-1.773760742	2.16E-10
Hacd1	335.167572	1.025735335	8.44E-10
Hacd4	313.082966	-1.146372209	0.00012185
Hars	2647.78974	1.167810001	1.84E-10
Haus4	1259.65221	1.026568147	7.38E-06
Havcr2	148.43654	1.955146283	9.17E-17
Hax1	1027.19904	1.517018505	1.35E-12
Hbegf	158.77469	1.354683862	1.92E-05
Hbp1	1142.3151	-1.146752522	7.22E-10
Hck	89.1746981	-1.805785678	2.39E-20

Hcst	1245.99053	-2.602168897	1.74E-37
Hdac9	301.78941	-1.290732896	0.00065639
Hdgf	8215.63276	1.190715007	7.09E-07
Hdhd5	2716.35586	-1.070136657	2.38E-07
Hdlbp	10961.779	1.119283124	7.17E-15
Hells	4898.64878	1.435488899	3.19E-08
Hemk1	816.528395	1.594370177	1.80E-11
Herc3	6530.68999	-1.225689347	3.01E-08
Hhat	750.697804	1.922281289	4.41E-22
Hid1	1386.13307	-3.674552999	8.36E-87
Higd1a	2028.39223	1.020986917	0.00020713
Hip1	1934.04049	2.798668961	2.19E-11
Hipk2	5183.61908	-1.762270703	1.32E-12
Hirip3	1038.36921	1.595215189	1.29E-11
Hist1h1a	7387.31266	1.262615029	0.00760273
Hist1h1t	144.410267	1.908131645	1.16E-10
Hist1h2ah	1028.79413	1.517138127	0.0007381
Hist1h2bc	5590.87618	1.741484756	3.56E-05
Hist1h2bh	5514.26928	1.073331896	0.01045003
Hist1h2bj	4287.19091	1.903850236	5.04E-06
Hist1h2bk	3528.31875	1.316933342	0.00155439
Hist1h2bl	3173.45331	1.040095959	0.01368342
Hist1h2bm	6476.30387	1.089085612	0.00746345
Hist1h2bp	1034.77244	1.050150682	0.01367865
Hist1h3a	4909.22746	1.23019769	0.00595776
Hist1h3c	12510.639	1.074499899	0.02612949
Hist1h3e	10344.9022	1.091620069	0.01287906
Hist1h3g	2557.86521	1.086944895	0.02440511
Hist1h4i	1124.73805	1.007919521	0.02486412
Hist2h2ac	3093.32229	1.162418594	0.00583434
Hivep3	6600.21144	1.836570572	1.85E-28
Hk2	3921.05	1.961926052	8.16E-10
Hmbs	1065.75839	1.154600329	3.86E-09
Hmga1	2283.48251	1.087252143	2.08E-08
Hmgb3	810.156995	1.625166703	3.23E-11
Hmgcr	8121.50914	1.356744711	5.50E-10
Hmgcs1	3598.44532	1.341206993	1.01E-07
Hmgn1	2964.34024	1.365934611	4.86E-11
Hmgn3	146.664589	1.87130634	6.24E-21
Hmgn5	887.837233	1.017218425	0.0002897
Hmmr	2872.54174	1.315253178	2.73E-05
Hmox1	137.428856	1.197552749	2.32E-05
Hn1l	1471.6015	1.05485641	4.34E-12
Hnrnpa1	17072.4031	1.314607221	1.82E-14
Hnrnpab	8386.5265	1.602956534	3.32E-10
Hnrnpll	1823.97617	1.766342862	2.68E-42
Hopx	916.380263	-1.092469431	6.24E-08
Hpcal1	7659.41033	-1.448988158	1.98E-08
Hpgds	453.574409	-1.329400992	6.80E-09
Hras	845.371416	1.198112572	1.11E-05

Hsd17b11	798.818012	-1.000589724	1.04E-12
Hsdl1	1504.21685	-1.036287898	2.21E-09
Hsp90aa1	18617.063	1.454597073	1.21E-05
Hsp90ab1	58251.7437	1.208646258	5.21E-07
Hsp90b1	7801.98477	1.038349391	0.00030606
Hspa13	551.153931	1.145313208	7.01E-05
Hspa4l	2061.38899	1.248957852	5.77E-16
Hspa9	10623.5225	1.477652295	3.71E-27
Hspd1	10665.7566	1.981676529	1.01E-24
Hspd1-ps3	160.591	1.23217881	4.96E-07
Hsph1	2937.31543	1.017719783	0.00106255
Htra2	468.130555	1.209693287	1.85E-12
Hunk	128.154915	1.530263847	1.64E-09
Hyal2	226.126328	1.201623869	4.57E-06
Hyou1	4553.05729	1.500732071	1.47E-08
Iars	5802.01462	1.700977798	3.44E-40
Icos	3287.90269	1.506772138	5.33E-51
Idnk	1381.43423	-1.131164431	1.94E-09
Ier5l	161.104934	1.140258745	0.00071345
Ifi206	3378.22077	-2.722066099	1.53E-35
Ifi208	2469.79701	-3.425119265	4.66E-57
Ifi209	3965.6784	-2.993903878	2.22E-44
Ifi213	2143.96902	-3.58835605	2.86E-28
Ifi214	1587.01952	-2.693999187	2.30E-30
Ifi30	587.949475	1.317424924	8.26E-06
Ifit1	183.154463	-1.364362854	7.05E-06
Ifit1bl1	519.627819	-3.442500222	3.48E-35
Ifit1bl2	124.903973	-1.212430403	4.04E-06
Ifit3	197.781959	-1.486864419	1.19E-12
Ifitm1	1930.87479	-2.50591708	0.00137777
Ifitm10	326.992945	-2.279378886	5.28E-16
Ifnar2	1706.9671	-1.242424395	1.29E-09
Ifng	1218.72912	3.826975051	6.92E-12
Ifngr1	12643.5946	-1.024366817	4.24E-09
Ifngr2	1311.28513	-2.042855855	8.82E-25
Ifrd2	1130.9832	1.192807175	2.38E-13
Ift43	160.124052	1.370387349	4.18E-11
Igf2bp2	351.545432	-2.555894921	8.16E-15
Igf2bp3	1320.83122	1.006712824	3.21E-05
Igfbp4	3108.27514	-3.078706832	1.88E-17
Igfbp7	1728.99254	4.428327916	4.60E-96
Ighj4	129.477779	-1.006000162	7.33E-10
Igsf3	384.320021	-1.320476733	1.19E-07
Igsf8	652.585425	1.624595619	1.47E-12
Iigp1	2281.44971	-3.139943643	4.62E-22
Ikbke	3283.67245	-1.28129336	8.66E-08
Il10rb	1638.69479	-1.10577361	2.27E-27
Il11ra1	298.415642	-1.033565643	1.87E-08
Il12rb2	11602.7669	1.207012749	1.03E-09
Il16	568.986047	-1.305827735	4.48E-20

Il17ra	7216.99104	-1.28140011	7.79E-14
Il1rl1	95.5896715	1.308380903	6.81E-07
Il27ra	4278.78382	-1.568320507	1.50E-18
Il2ra	23588.2245	1.827737965	3.29E-11
Il4ra	11740.0029	-1.350309017	5.66E-16
Il6ra	7075.76139	-3.205178923	2.62E-19
Il6st	9655.86854	-1.979626211	3.04E-09
Il7r	6069.90292	1.018854919	4.40E-06
Impg2	198.437837	-1.187979686	9.13E-07
Inpp5b	6344.75932	1.141629955	1.48E-16
Inppl1	511.928584	1.896996162	3.41E-16
Insl6	238.704446	-1.044192132	1.31E-05
Intu	230.224279	1.708960162	6.95E-34
Ipo4	1536.17044	1.290562111	1.58E-09
Ipo5	9249.76824	1.207656772	2.79E-12
Irak1bp1	187.017775	1.355512305	1.25E-10
Irak4	1220.50424	-1.044694031	2.60E-16
Irf1	6084.24023	-1.988042873	1.03E-19
Irf4	5635.88149	1.974632893	1.80E-07
Irf5	448.565483	1.186077634	5.64E-08
Irgc1	149.572205	-1.378701558	2.49E-11
Irgm1	3251.2194	-1.3751818	9.39E-14
Irgm2	2478.74894	-1.824206906	2.16E-29
Itga2	390.446736	-2.554109055	4.59E-23
Itga3	340.539312	2.271313	2.17E-32
Itga4	7199.02581	-1.767207925	1.01E-14
Itga7	236.089348	-2.381597411	0.0001546
Itgae	4483.4634	-3.948670256	3.96E-42
Itgav	3544.66632	1.134194967	3.41E-14
Itgax	96.8103919	-1.56348821	2.24E-06
Itgb3	4529.41622	-1.573409665	9.08E-16
Itgb7	10963.8075	-2.087545038	1.68E-16
Itih5	2572.40964	2.012405298	1.22E-15
Itm2a	845.531519	1.497643293	7.59E-07
Itm2b	7886.30029	-1.169688154	2.38E-26
Itm2c	1724.96864	-1.256855931	3.93E-07
Itpr2	10807.9356	-1.856306347	1.43E-17
Itpr3	4998.50689	-1.146914767	1.11E-07
Itpripl1	1423.10709	1.258144173	2.89E-07
Izumo1r	221.541154	2.195454963	5.54E-24
Izumo4	548.780408	-1.783164455	7.99E-13
Jak1	32653.5647	-1.021784685	1.03E-17
Jakmip1	6583.60576	-1.729440425	7.49E-15
Jazf1	2462.44101	1.92469515	1.17E-10
Jcad	152.535058	1.274896191	2.03E-10
Jun	2886.52302	-2.0242816	4.56E-07
Junos	476.489088	-1.435948999	7.23E-13
Kat2b	6610.15855	-1.034081548	8.44E-05
Kbtbd11	6770.16403	-1.588671657	1.14E-29
Kbtbd3	359.716036	-1.036456303	1.04E-05

Kcna2	487.024353	-2.538672208	1.65E-28
Kcnc1	337.551733	-2.444998546	5.32E-15
Kcnh2	245.251396	-1.720671687	4.03E-12
Kcnh4	328.032974	-1.227857134	8.34E-06
Kcnh6	1596.32019	-1.296155294	0.00099771
Kcnj15	129.359262	-1.109554245	1.45E-05
Kcnk10	557.392452	4.226694962	3.49E-46
Kdelc1	354.258988	1.528436851	4.38E-19
Kdelc2	504.168688	1.276159278	3.73E-12
Kdm3a	7293.92848	-1.00483562	6.12E-16
Kdm6b	1834.31545	-1.00759355	0.00014579
Kdm7a	2435.10447	-1.483017039	5.95E-10
Kif15	5952.51445	1.335642502	1.25E-07
Kif20a	1910.09182	1.177887609	9.43E-05
Kif20b	3284.71885	1.024455932	5.91E-05
Kif21b	14449.6092	-1.186990192	2.06E-06
Kif24	2341.82298	1.049667115	3.65E-05
Kif2c	1660.37926	1.491862362	9.84E-08
Kif4	3525.42482	1.125204914	5.06E-05
Kif5a	125.918968	1.074839512	1.47E-07
Kifap3	576.836567	1.481035124	5.49E-18
Kifc2	176.847731	-1.469063474	6.65E-10
Kirrel	1309.81411	-4.418138481	2.04E-38
Kirrel3	391.266254	-1.120267099	1.75E-08
Klc1	4769.41814	1.14617815	2.34E-05
Klf11	844.18161	-1.419787036	0.00041352
Klf13	17988.8193	-1.111235717	1.66E-11
Klf2	5222.4889	-3.163892284	1.98E-25
Klf3	5220.38629	-4.090776312	4.30E-55
Klhdc1	1634.75712	-2.163676746	6.54E-09
Klhl23	126.099446	1.412524943	1.27E-09
Klhl24	2536.50541	-1.19634872	3.56E-15
Klhl3	986.971671	-1.093828647	0.0002281
Klhl30	98.2650812	-1.108090135	4.99E-06
Klhl42	1637.74439	-1.012193247	0.00043338
Klhl6	6474.64204	-1.311362698	1.22E-15
Klk8	1330.44691	-1.566257998	9.62E-09
Klra5	285.972236	2.987763384	2.43E-19
Klrc1	1856.13167	3.932439411	9.61E-41
Klrc2	410.852346	2.838991075	5.31E-19
Klrc3	107.025013	1.272139414	5.05E-06
Klre1	214.565099	1.806807477	5.84E-13
Klri2	202.307501	1.967504571	1.27E-19
Klrk1	5915.04456	2.167615661	9.55E-11
Kmt2c	12827.7197	-1.022473688	1.17E-10
Kntc1	3880.81387	1.516031647	7.39E-11
Kpna2	4598.87578	1.332488567	2.43E-06
Krba1	659.419795	-1.908999707	2.71E-12
Kremen2	139.938633	-1.579738193	5.24E-10
Ksr2	531.372817	-2.761832356	3.14E-13

Kyat3	356.423342	1.141633716	2.87E-11
L1cam	564.730601	-1.668417871	5.60E-20
L2hgdh	499.834476	1.231199998	6.88E-18
Lacc1	104.030339	1.158450449	4.44E-10
Lad1	278.856597	3.408781719	2.04E-05
Lag3	2550.40185	2.060747606	2.46E-12
Lair1	956.178572	-2.225655718	1.07E-08
Lama5	411.736164	1.675841844	5.88E-07
Lap3	1843.54599	1.074423064	2.11E-07
Lars	4814.09867	1.286775233	8.99E-21
Lats2	407.597589	-1.686466005	8.66E-15
Lca5	815.899278	-1.165833049	7.31E-07
Lcn4	328.624238	-2.125019313	5.12E-15
Ldha	40325.6603	1.765312543	8.36E-07
Ldhb	217.524347	1.570739618	1.25E-15
Ldlrad1	107.501527	-1.081449931	7.14E-07
Ldlrad4	3339.02395	-1.931959938	2.23E-11
Ldlrap1	1718.06162	-1.757834346	7.99E-13
Lef1	22969.8144	-1.831554094	2.56E-14
Leng9	265.462708	-1.245507776	9.32E-07
Lfng	7791.512	-1.366328397	1.49E-09
Lgals8	2059.85196	-1.378329939	7.22E-15
Lhfpl2	162.198972	1.598988717	3.41E-12
Lhfpl3	212.630084	-1.719394596	1.34E-10
Lif	597.647527	1.08694008	0.02197496
Lig1	5627.21513	1.297215526	6.16E-06
Limd2	10800.3894	-1.087795982	2.48E-06
Lipg	186.26325	2.136443414	8.95E-14
Litaf	1090.80485	1.602957864	1.09E-14
Lman1	1879.77449	1.268136796	3.27E-25
Lmbr1l	1816.75493	-1.002874296	1.76E-06
Lmf1	2343.1752	-1.018272675	4.45E-05
Lmnb2	1236.08474	1.006212413	5.76E-08
Lmtk3	466.130978	1.036295108	0.00056867
Lncpint	13041.5587	-1.342063784	1.61E-06
Lonp1	3985.32145	1.668301049	3.78E-12
Lonp2	7871.01641	-1.075512181	8.45E-08
Lonrf3	193.491459	1.075069982	3.46E-07
Lpar3	120.751903	1.825595592	5.05E-18
Lpgat1	2477.67032	1.056771552	1.70E-08
Lrmp	7299.20991	1.025393493	1.73E-13
Lrp8	1277.81227	1.753587001	5.72E-17
Lrr1	330.868058	1.247740672	1.34E-08
Lrrc3b	128.246394	-1.076621589	5.91E-05
Lrrc49	193.264122	1.95342683	6.07E-31
Lrrc59	3904.70881	1.125735346	3.37E-08
Lrrc61	512.497648	-1.191994332	1.37E-08
Lrrc75b	312.686566	-2.21274227	9.18E-15
Lrrc8a	173.086375	-1.193386727	1.77E-07
Lrrc8d	7854.48924	1.020017095	0.00075766

Lrrk1	1614.14883	1.782811052	1.78E-11
Lrrk2	126.359391	1.488948289	1.41E-09
Lsm2	960.239237	1.143624091	2.87E-07
Lsm3	964.952749	1.058930171	8.36E-06
Lsp1	7781.73098	-1.067584927	1.11E-12
Lss	841.704245	1.037592089	6.10E-06
Ltb	5681.39537	-2.291841247	3.14E-10
Ltbp3	165.433657	1.974153454	7.44E-15
Ly6a	2832.31293	-1.693918399	9.43E-06
Ly6c2	3140.80193	-2.125859577	5.99E-10
Ly6g5b	126.253772	-1.601484887	6.94E-08
Lyar	1922.70832	1.577796234	6.25E-18
Lyn	305.055039	1.002084367	0.00079452
Lynx1	109.811693	-1.410298972	6.56E-13
Lypd6b	1504.83267	-3.299782819	3.59E-28
Lysmd1	390.446631	-1.012658339	8.97E-05
Maats1	515.81656	4.419636497	2.80E-119
Macf1	51087.4465	-1.017235123	1.47E-14
Madd	8582.06223	-1.02934828	3.99E-16
Maff	640.770022	-1.239690763	0.0036069
Mafg	932.748779	1.415524055	5.55E-12
Maged2	203.069742	1.372114265	1.87E-09
Malat1	63054.595	-1.162704398	0.00055033
Mamdc2	192.002329	2.264633064	1.67E-09
Maml2	32362.5724	-1.183717325	0.00021197
Maml3	10282.0519	-1.128343191	2.48E-05
Maneal	208.63906	-1.175931704	3.50E-11
Manf	2092.6358	1.094844536	0.00014175
Map3k20	360.289058	3.377877109	1.74E-42
Map3k8	4629.55279	-1.448228357	4.04E-07
Map4k3	762.4649	-1.027443716	5.07E-08
Map9	434.184131	1.923572802	1.10E-23
Mapk1ip1	1009.19663	-1.482737751	8.33E-08
March3	1904.49372	-2.274882177	8.42E-14
March8	304.575967	2.366939304	4.14E-37
Marcks	116.295154	1.082681847	2.88E-07
Marcksl1	628.797687	2.411164136	7.33E-13
Mars	3452.46167	1.788840554	1.04E-19
Marveld2	401.652107	1.351836607	1.27E-15
Mast2	3313.15719	1.306001334	2.28E-19
Mastl	1916.34723	1.052329247	0.0001145
Matk	814.104739	-1.343294358	4.04E-09
Mbp	9518.87791	-1.158435018	1.82E-11
Mcm10	2544.52016	1.860793234	5.88E-13
Mcm2	5874.94855	1.23566167	1.19E-05
Mcm3	8687.95646	1.346828483	1.84E-06
Mcm4	7644.59954	1.085355389	1.85E-05
Mcm5	8887.73617	1.33763104	1.23E-05
Mcm6	12305.3399	1.251034976	1.72E-07
Mcm7	7424.34228	1.250367692	8.36E-06

Mcm8	825.93972	1.427297934	7.47E-12
Mcoln2	2274.54466	-1.214752986	3.22E-06
Mcoln3	171.221362	-1.498662886	7.82E-13
Mctp1	398.925802	2.332991908	1.87E-07
Mctp2	7873.48627	-1.356656256	3.98E-07
Mcu	2447.80919	1.486598902	9.21E-09
Mdh2	5631.09012	1.071573888	8.84E-07
Megf11	1657.8718	-1.195774751	5.86E-06
Megf8	114.412818	1.665732164	9.33E-09
Melk	1559.8724	1.536242265	1.03E-08
Mettl27	741.891311	-2.796592082	7.86E-37
Mex3b	301.566279	-1.03039447	4.29E-05
Mfhas1	3106.86557	1.176539559	2.47E-05
Mfsd13a	597.542921	1.553552787	1.36E-13
Mfsd2a	622.232293	1.173887513	0.00012029
Mgat4a	4856.69162	-1.230829146	7.03E-07
Mgme1	474.518255	1.13460876	3.09E-15
Mgst2	7055.33618	-1.988612479	6.86E-09
Mical3	928.450575	1.334498333	4.40E-09
Mif	6499.77393	1.399530437	3.56E-05
Mir155hg	1906.74568	1.649478445	1.35E-10
Mki67	36660.7574	1.280349027	9.98E-06
Mlf1	155.668506	1.029052477	0.00341558
Mlf2	2286.97298	1.036067262	0.00030258
Mlkl	695.007632	1.149391487	1.43E-07
Mmaa	437.575442	-1.221556835	0.00017368
Mmd	1126.90187	1.05774752	3.81E-16
Mmp15	127.412365	-1.551258885	1.76E-09
Mnd1	631.464345	2.187418799	1.41E-17
Mndal	5696.5809	-1.84155485	8.46E-12
Mob3c	756.323581	-1.138674184	1.17E-13
Mogs	2723.23498	1.351229236	3.45E-07
Morc1	120.018992	-1.890821897	8.40E-15
Morf4l2	2924.70615	1.213738878	1.42E-07
Morn4	121.686407	1.283326242	2.57E-15
Mospd1	637.769053	-1.076314963	6.20E-17
Mpp6	2522.61966	1.169513927	3.94E-10
Mppe1	1967.54227	-1.083097119	4.79E-11
Mpv17l	549.54625	-1.118879731	0.00203998
Mpzl2	127.913127	1.788560404	1.78E-08
Mpzl3	856.076809	2.243386779	2.42E-23
Mrgpra6	120.351718	1.937207504	1.96E-10
Mrgpra9	91.8556849	1.361063757	9.85E-07
Mroh3	115.61829	-1.264975187	8.19E-05
Mroh5	210.935986	-1.465496461	6.15E-16
Mrpl11	805.907387	1.029885639	5.17E-06
Mrpl12	1032.60247	1.081323424	0.00036212
Mrpl28	1015.6892	1.230002866	9.32E-09
Mrpl45	1399.82768	1.286416984	1.19E-10
Mrpl51	1260.41529	1.023541781	3.21E-05

Mrps6	1217.7885	1.558720794	1.62E-16
Mrps7	1232.77548	1.118314582	8.08E-08
Mrto4	1464.48122	1.317142681	9.61E-11
Mrvi1	159.229917	-1.218637085	2.58E-10
Ms4a4b	20649.9889	-2.339642389	2.91E-59
Ms4a4c	1238.95371	-1.675386034	0.0001895
Ms4a4d	119.82684	-1.087581069	4.79E-11
Ms4a6b	17552.1342	-1.8339251	1.04E-42
Mst1	127.308807	-1.022803781	3.80E-08
Mt1	550.962795	2.927040817	4.36E-12
Mt2	145.941008	2.059783106	1.66E-10
Mtbp	2682.34484	1.28822736	2.67E-13
Mtf2	3506.61256	1.096898517	2.93E-06
Mtfp1	121.88191	1.051755377	4.33E-05
Mtfr2	644.868993	1.388780344	1.70E-08
Mthfd1	2872.78131	1.147721163	5.19E-18
Mthfd2	2650.43928	1.718973355	7.23E-27
Mturn	1668.24303	-3.361998374	5.26E-43
Mx2	231.971584	1.769434132	5.56E-07
Mxd4	1455.02323	-1.754766964	7.32E-20
Mxi1	2390.41516	1.710504637	1.98E-10
Myb	2303.05157	1.194006337	0.00098509
Mybbp1a	9517.85905	1.236359361	9.25E-09
Mybl2	2062.20642	1.82105315	8.06E-15
Mybpc3	199.67689	-1.594202864	8.59E-24
Myh10	1583.71457	1.831166964	1.36E-20
Myl10	682.591741	-2.823910673	1.07E-20
Mylip	1496.19871	-3.200911173	8.76E-97
Myo10	3878.51188	3.967205566	6.40E-38
Myo16	170.594695	1.352599115	3.22E-06
Myo18b	124.446528	-1.523353664	1.67E-07
Myo19	639.397629	1.300249435	8.13E-26
Myo1e	3576.81101	2.087908809	4.32E-14
Myo1f	4739.34201	-1.457166277	1.97E-13
Myo3b	2100.91731	-4.027802915	4.56E-27
N4bp2l1	1586.88697	-1.344934997	3.62E-06
Nab2	977.694159	1.861537478	3.77E-12
Nacad	362.79626	-1.707256301	1.19E-20
Nacc1	4432.75878	1.040577999	1.48E-06
Nacc2	489.19298	-2.5094563	1.14E-19
Nars	4933.9902	1.413985648	3.22E-18
Nasp	5825.56813	1.395724946	3.54E-09
Nav1	309.38892	1.322044828	0.00024145
Nav2	511.719521	-1.300534756	0.00042597
Ncald	697.973359	-1.614305461	1.79E-08
Ncapd2	8980.86492	1.028094886	0.00026476
Ncapg	2917.39408	1.140351381	0.0003461
Ncapg2	6608.73656	1.203000263	5.81E-06
Ncaph	2934.90067	1.279611031	2.50E-07
Ncf1	1364.30883	-1.180424684	9.86E-08

Ncf4	1573.32947	-1.023840064	4.31E-15
Nck2	5124.49375	-1.41511155	1.19E-05
Nckap5l	1014.17887	-1.729272843	4.35E-13
Ncl	33857.2861	1.028167757	1.03E-07
Ncs1	460.697057	3.294875515	2.48E-48
Ndc1	2073.24253	1.884698399	1.84E-35
Ndc80	2309.0271	1.056067039	8.32E-07
Ndufa4	1662.54396	1.014248652	3.26E-05
Neat1	5424.40716	-1.205582873	0.00141342
Neb	4468.73402	1.947569839	9.46E-23
Nedd4l	5871.84214	-1.984998745	1.86E-16
Nefh	1436.38551	1.477448973	3.64E-08
Nek2	1505.60035	1.231654011	2.83E-05
Nek4	356.660684	1.528945715	5.51E-31
Nek6	2091.00486	3.496831381	1.70E-53
Nemp1	1727.5495	1.045051439	2.09E-12
Nespas	212.929148	1.094132017	0.00049964
Neurl1b	699.78736	-1.183313389	7.63E-05
Nf1	2495.13373	1.689999236	2.26E-19
Nfat5	6797.68009	1.767291984	3.85E-22
Nfe2l3	599.915644	-1.39123543	8.12E-05
Nfia	1803.35084	-1.544393316	5.70E-06
Nfil3	1047.13631	1.406639005	0.0005191
Nfkbia	4126.51645	-1.110391809	0.01182099
Nfkbid	1731.04214	-1.042919118	0.00117541
Nfkbie	1363.05209	-1.153679378	7.11E-07
Nfkbiz	4175.60207	-1.216950864	2.49E-08
Ngly1	6886.31256	-1.018544119	1.29E-05
Nhp2	2497.26439	1.133846268	5.36E-05
Nhsl2	198.317252	-1.041808001	1.37E-05
Nid1	140.20072	2.222568903	4.40E-15
Nin	9757.85797	-1.413440076	5.37E-56
Nipa1	165.768913	-1.290738747	4.45E-05
Nipal1	840.416483	-2.334808804	2.61E-39
Nipal3	2833.73699	-1.396243668	4.03E-18
Nkain1	184.400684	2.516876399	2.19E-29
Nkd2	266.375182	-1.080869126	2.62E-07
Nlgn2	785.965417	1.58010638	1.18E-09
Nlrp1b	88.8656313	-1.173771607	1.10E-05
Nmi	1671.09638	-1.127610796	6.29E-15
Nmnat2	295.480033	-1.353413146	4.78E-10
Nmral1	458.057041	1.059008706	9.62E-09
Noc2l	4556.05928	1.028001672	1.78E-09
Noc4l	1746.15232	1.243340582	8.21E-08
Nod1	2845.56198	-4.576110785	1.32E-76
Nod2	116.669509	-1.582286669	4.24E-17
Nol4l	555.174698	2.550231923	9.24E-31
Nolc1	4808.12239	1.443413622	1.08E-13
Nop16	1160.67566	1.267238072	3.37E-12
Nop2	3132.4633	1.179167606	4.00E-13

Nop56	6630.85843	1.288686379	1.20E-16
Npas4	194.298562	1.93771565	9.46E-27
Npc2	6908.32299	-1.405870988	3.10E-18
Nphp1	215.659324	2.289761993	7.97E-36
Nphp3	287.601715	1.063466488	2.69E-08
Nr1d1	177.208351	-1.268335027	1.12E-09
Nr1d2	1649.22696	-1.845197141	1.81E-18
Nr4a2	2109.02875	1.316009554	0.00170503
Nr4a3	4423.78988	2.725083292	2.97E-10
Nrg2	328.172862	-2.333343443	3.49E-10
Nrgn	1280.23044	4.34912353	3.06E-41
Nrip3	163.429863	-1.225345944	7.43E-06
Nrn1	855.25792	1.544690673	2.60E-08
Nrp1	1656.84254	3.622080633	3.81E-22
Nrp2	200.35268	-1.579825219	1.26E-08
Nsd2	10718.0279	1.016163151	1.11E-09
Nsd3	24520.2335	-1.445650244	1.36E-12
Nsdhl	1032.95291	1.046644434	1.86E-11
Nsg2	6745.11001	-5.24536507	3.40E-101
Nsl1	1343.38043	1.669453669	2.35E-11
Nt5dc2	397.289465	1.711242914	1.85E-10
Nudc	3240.72586	1.199327684	1.87E-10
Nudcd2	1220.51081	1.538383405	1.01E-16
Nudt14	845.487829	-2.332623881	1.09E-26
Nudt2	305.671044	1.072505025	1.18E-10
Nudt5	2366.14196	1.120010122	4.50E-21
Nudt6	522.630488	-1.121363418	3.31E-05
Nuf2	2063.98216	1.003313578	0.0011886
Numbl	230.40875	-1.007298309	3.25E-09
Nup107	3893.11347	1.024477414	2.62E-24
Nup155	4546.34831	1.141455186	3.34E-19
Nup160	4386.29793	1.074344666	3.74E-21
Nup205	7117.89725	1.17512222	1.95E-15
Nup37	1094.14112	1.028175756	9.21E-11
Nup85	3411.06007	1.299653028	1.14E-19
Nxnl1	133.499498	-1.637842258	2.58E-10
Nxpe3	3354.16684	-1.05430801	1.48E-05
Oas1b	875.211713	-1.189370691	0.00102901
Obscn	132.826835	-1.392169216	4.30E-09
Obsl1	143.150611	1.194287007	1.57E-05
Oc90	163.707259	-1.1548407	5.42E-07
Ocel1	356.914931	-1.158621767	7.26E-09
Ocln	154.695594	1.976031769	2.98E-13
Odc1	6784.3945	1.573495553	6.62E-10
Olfml3	425.387415	-1.830337276	5.24E-14
Olfr172	120.250703	-1.860489017	5.87E-11
Olfr524	182.600306	-1.740428072	1.67E-08
Olfr56	1640.54607	-1.570659597	1.97E-13
Olfr755-ps1	101.287323	-1.054546891	1.86E-07
Olfr99	158.856371	-1.249760403	1.27E-05

Oprm1	426.694788	-1.620968543	2.28E-06
Orai2	4999.68156	-2.039069972	9.82E-22
Orc1	522.066162	1.448159036	1.25E-10
Orc6	1528.97258	1.337705921	5.55E-11
Osbpl1a	265.174248	1.2854906	3.38E-07
Osbpl5	1700.45747	-1.09488937	4.64E-16
Osgin2	980.919591	1.201456068	1.05E-13
Osm	1333.07867	-1.538058796	6.33E-09
Otud1	272.137256	-1.108914469	1.85E-09
P4hb	9187.11622	1.123002957	0.00020445
Pa2g4	8275.02641	1.308595239	8.51E-10
Pabpc4	4973.553	1.667352781	1.05E-20
Pacs1	5617.17314	-1.200276067	1.68E-13
Padi2	889.677616	-1.13549106	7.79E-10
Pag1	7662.63137	-1.637980082	1.20E-15
Palb2	836.461193	1.275682782	9.21E-11
Palm3	834.622448	-2.752170638	2.42E-14
Pank1	551.508779	1.078007927	7.15E-07
Panx1	3049.14332	-1.162047777	6.38E-32
Paqr7	882.828567	-1.100974987	9.51E-09
Pard3b	214.521672	2.302270011	6.91E-18
Parm1	182.996731	-2.225112554	1.11E-06
Parp12	494.333799	-1.610755532	2.60E-16
Parpbp	1525.62224	1.208499225	6.05E-06
Parvb	92.7355155	-1.548383039	2.28E-11
Pask	968.621333	1.45530546	3.74E-11
Patj	3985.59205	-1.822223302	3.66E-09
Patl2	752.27633	-1.262784488	5.15E-13
Pbk	921.193336	1.062101342	6.80E-05
Pbx3	764.819291	1.605104983	3.36E-05
Pced1b	9611.70775	-1.460781028	6.99E-06
Pck2	974.749306	2.52698098	1.84E-13
Pcmtd1	3963.2042	-1.139985939	1.24E-05
Pcnx2	94.2513931	-1.231102492	1.56E-06
Pcx	927.894557	2.163460446	2.47E-38
Pcyox1l	358.471865	1.25492174	2.81E-12
Pcyt1a	2184.2779	1.296728306	9.71E-09
Pdap1	1854.07278	1.0191951	2.68E-10
Pdcd1	2789.41828	3.375651186	4.22E-20
Pdcd1lg2	472.111964	2.69370302	8.68E-24
Pdcd4	9452.33353	-1.76440182	1.14E-30
Pde10a	111.613031	1.388111052	0.00011054
Pde1a	166.395675	2.522723086	2.52E-29
Pde3b	23989.3699	-1.202764023	8.03E-05
Pde4a	268.581578	1.658659925	4.21E-17
Pde5a	368.810357	-1.433424865	1.11E-13
Pde6a	152.68815	-1.249684168	2.83E-07
Pde6b	238.455731	-1.186902518	1.31E-09
Pde8b	227.280017	-1.284229846	2.71E-07
Pde9a	169.28837	-2.354602569	1.35E-13

Pdia6	3763.28622	1.492081266	3.00E-09
Pdk2	204.651703	-1.40465985	2.54E-12
Pdlim4	1119.82527	-2.575478693	3.79E-11
Pdlim5	3502.84366	-1.264359614	1.53E-09
Pea15a	1451.03622	-1.027187065	8.10E-13
Peli1	21077.0835	-1.483231713	2.41E-06
Pelp1	2046.07831	1.250452501	4.48E-13
Penk	123.551385	1.985431332	2.29E-11
Pepd	4361.32507	-1.036207371	7.26E-07
Perp	252.131409	2.491274616	1.52E-20
Pex11g	949.956614	-2.396616276	1.44E-65
Pfas	4781.96089	1.969134405	2.26E-27
Pfkl	4437.34421	1.041874095	0.00226691
Pgam1	3635.1459	1.486799401	2.19E-08
Pgam2	346.903781	-1.356781207	1.81E-15
Pgk1	14642.3481	1.395622776	0.00021391
Pglyrp1	508.162218	-1.524444343	3.62E-09
Pglyrp2	1458.02946	-2.69271351	9.03E-56
Pgp	651.885161	1.445163163	9.98E-06
Pgpep1l	789.296133	-2.539243817	3.25E-29
Phactr2	3437.33489	2.698305816	2.38E-31
Phb2	2838.85244	1.364840576	6.54E-08
Phf1	721.598853	-1.790594394	9.30E-16
Phf11c	1050.32823	-1.551386212	3.23E-11
Phf21a	8300.85134	-1.110549999	0.00016048
Phgdh	6510.06299	1.553851955	6.01E-13
Phlda3	202.260328	1.775254012	1.61E-08
Phldb1	340.895234	1.427641784	1.97E-07
Phldb3	335.12135	-1.060570406	1.73E-06
Phtf2	2413.9239	1.022054375	1.81E-05
Pi4k2b	771.56173	1.278952392	6.91E-29
Pidd1	901.292984	1.214141076	4.09E-09
Pigv	970.381923	-1.159177164	4.64E-07
Pigz	203.410282	-1.548182072	1.83E-16
Pik3ap1	2262.08821	1.003718987	0.0009806
Pik3c2b	258.498919	1.082818583	2.80E-08
Pik3ip1	3258.40359	-2.543626567	9.79E-27
Pik3r2	510.106017	2.169704426	2.60E-26
Pik3r5	10971.4842	-1.508254525	1.26E-33
Pilrb2	92.1646515	-1.317057188	1.44E-05
Pim3	1030.41306	1.209608941	3.10E-05
Pink1	1481.82204	-1.44770486	4.26E-09
Pisd-ps1	716.031589	-1.098822646	1.49E-07
Pitpnc1	22864.7563	-1.050843412	2.75E-06
Pitpnm2	5297.43689	1.017263872	9.43E-07
Pkd2	95.3201734	1.365612037	3.22E-11
Pkig	578.64223	1.5903858	3.71E-38
Pkm	35870.2944	1.013063676	0.00947294
Pkmyt1	1192.82082	1.106277059	6.57E-07
Pkn3	313.349622	1.877783684	8.71E-30

Pkp2	169.164216	1.648139972	1.45E-08
Pla1a	493.528209	2.7098868	5.28E-23
Pla2g4f	124.578699	1.465816073	1.79E-12
Plac8	9490.35822	-1.486806524	8.29E-08
Plagl2	2971.84238	1.275749789	1.67E-15
Platr10	81.5527048	-1.12507354	3.26E-05
Plbd1	173.092251	-1.018445005	1.12E-05
Plcb2	2298.68161	-1.764978883	1.63E-17
Plce1	380.311648	-2.771885577	7.64E-25
Plcl1	1767.84578	1.563848764	1.07E-09
Pld4	84.9941604	-1.335760579	1.09E-13
Plek	3620.22871	1.76879265	1.37E-09
Plekha6	770.260544	-2.292914339	6.28E-21
Plekha7	501.341665	1.396165821	2.32E-09
Plekha8	171.647213	1.589674538	4.08E-15
Plekhg3	737.174784	-1.36881567	1.86E-07
Plekhg6	133.432065	-1.091188941	9.39E-09
Plekho1	1141.31264	-1.599739829	1.33E-09
Plin2	479.48697	2.141358377	2.64E-14
Plk1	1838.04773	1.384928872	7.06E-06
Plk2	380.761209	2.362947985	9.70E-13
Plk4	2366.45642	1.216960698	1.22E-07
Plod2	1659.09267	1.273927518	4.78E-07
Plod3	596.213231	1.728133404	3.57E-13
Pls1	701.26224	1.102944251	0.00024114
Plscr1	2273.91886	2.167240754	3.74E-23
Pltp	244.037821	-2.287739735	1.42E-29
Plxdc2	2961.16146	1.026675922	1.81E-05
Plxna1	296.541349	1.756257179	3.59E-16
Plxna3	265.873933	2.548612992	1.97E-18
Plxnb2	173.263236	1.000493582	0.00478615
Plxnc1	4390.71623	2.060629896	8.47E-25
Plxnd1	380.819443	2.336629779	5.42E-09
Pmel	958.360025	-2.155370177	1.05E-56
Pmvk	850.517277	1.441330824	3.74E-18
Pnpla7	3067.46444	-1.86663925	6.67E-15
Pnrc1	4768.01624	-1.074815534	1.49E-07
Poc1a	896.251532	1.216202177	1.25E-11
Podn	215.920303	-1.182148093	1.15E-08
Podxl	230.449803	-1.040282265	3.79E-05
Pola1	10979.9116	1.266860557	0.00080554
Pold1	3594.89267	1.227426626	4.34E-06
Pole	5675.34609	1.268984839	5.10E-09
Pole3	1119.36987	1.341266861	1.72E-08
Polk	2026.99676	1.123427003	3.20E-07
Polq	2765.14322	1.764304912	2.91E-11
Polr2c	1473.16184	1.026892011	1.83E-05
Polr2e	1378.41718	1.400335122	2.43E-10
Polr2f	1133.0097	1.101791287	1.06E-07
Pop1	2541.74682	1.243842582	1.19E-25

Popdc2	140.947093	1.518884052	4.02E-15
Pou6f1	1035.13499	-3.499449175	1.03E-40
Ppa1	2553.31662	1.5480024	3.52E-14
Pparg	100.099209	1.343704002	2.54E-10
Ppargc1b	2652.31708	-1.067910606	0.00135806
Ppat	3244.1502	1.270564974	2.88E-15
Ppfibp1	1120.15163	1.630240193	5.30E-24
Ppil1	1899.83478	1.087087254	6.83E-09
Ppm1h	34371.2265	-1.734601482	9.59E-11
Ppp1r13l	203.830615	1.302552342	3.62E-08
Ppp1r3b	320.356424	1.114561336	0.0036423
Ppp1r9b	4228.1541	-1.069341967	9.56E-07
Pqlc1	343.385549	-1.188945265	1.83E-15
Prag1	1041.2221	-1.166773616	1.29E-05
Prdm1	529.355157	2.167514169	1.75E-10
Prdm11	1179.50041	-1.443527733	1.25E-09
Prdm5	304.261146	2.259646265	2.01E-12
Prdx2	2386.99221	1.092600168	1.41E-05
Prelid2	6207.05343	-1.169307159	1.38E-08
Prex1	14322.9912	-2.407362133	3.39E-50
Prf1	3655.60288	3.97534619	4.63E-27
Prickle2	129.588842	1.702462782	2.08E-15
Prim1	2141.35009	1.19169238	4.54E-07
Prim2	5960.15317	1.179781544	4.17E-06
Prkaca	1737.52817	1.07744887	1.19E-05
Prkacb	9440.63147	-1.61649778	1.16E-53
Prkar1b	209.536319	-1.69893397	2.13E-08
Prkcq	27287.501	-1.298342129	1.37E-11
Prkcz	974.269715	-2.410926639	6.49E-15
Prkd2	5927.52815	-1.081730819	1.44E-10
Prmt1	3641.03129	1.228721547	5.37E-09
Prmt5	2957.75143	1.308071869	2.09E-09
Prmt8	83.4476107	1.094446017	1.38E-08
Prpf19	3958.95074	1.241981544	3.75E-08
Prpf40b	269.938432	1.151783782	1.20E-06
Prr29	161.459921	-1.27126865	7.86E-10
Prr33	203.000948	-1.460727932	6.60E-17
Prr5	660.235447	-1.062886669	0.00045971
Prr7	1150.54191	-1.924381022	7.87E-18
Prrg1	2024.70415	-1.101803843	0.00156344
Prrt1	553.115579	-1.458443304	8.13E-08
Psap	8460.37838	-1.048860854	7.95E-07
Psat1	4834.87525	2.130311926	5.41E-25
Psmb5	1686.0189	1.133996305	4.83E-05
Psmb6	2563.75452	1.287664243	9.28E-07
Psmc1	3603.57493	1.037099099	2.26E-08
Psmc2	3160.50901	1.081088639	1.03E-09
Psmc3	3816.98281	1.044719417	4.85E-05
Psmc3ip	355.628693	1.126325184	1.05E-11
Psmc4	3029.7318	1.090745127	1.19E-06

Psmc5	2349.36225	1.257485927	2.39E-09
Psmd1	4227.60035	1.14676746	2.88E-15
Psmd12	2617.36893	1.057034656	3.38E-14
Psmd2	6567.75033	1.163975143	2.83E-08
Psmd3	3595.77809	1.049080701	1.19E-05
Psmd7	2336.87565	1.181663565	2.88E-12
Psmg1	716.352059	1.220462804	1.74E-16
Psrc1	477.528667	1.611854265	1.05E-10
Ptch1	4924.07597	1.23808765	1.31E-11
Pter	309.428591	1.85794843	8.38E-19
Ptges3	5150.95623	1.025743949	3.26E-10
Ptges3-ps	333.564473	1.002338261	3.75E-05
Ptgfrn	887.176493	3.440042284	1.23E-35
Ptgr1	119.740806	1.334103113	1.67E-08
Ptk2	454.725104	1.079935907	2.08E-07
Ptma	22492.2651	1.002823625	0.00083859
Ptms	1848.03386	1.079723016	0.00168619
Ptpn14	405.382051	-1.303797171	3.79E-08
Ptpn18	1692.38072	-1.319794193	4.64E-06
Ptpn3	1537.24251	2.833125178	1.19E-41
Ptpn5	1353.58291	4.622370409	1.31E-91
Ptpn6	6818.77503	1.091593624	3.09E-07
Ptpn7	3680.55318	1.525337981	7.06E-08
Ptprk	181.856509	1.053795505	0.00017719
Ptprs	5910.70197	1.586738454	1.06E-12
Ptprv	618.980115	1.439704146	7.79E-05
Pts	1235.38024	-1.209373152	2.21E-09
Pycr1	158.827907	2.0927061	5.94E-19
Pygl	463.285668	-2.094889782	0.00215393
Pygm	569.63478	-2.444035869	1.30E-28
Pyroxd2	133.496813	-1.808658781	2.78E-10
Qprt	522.069379	-1.027996492	0.00013263
Qser1	2155.92964	1.27704973	2.49E-11
Rab20	403.355156	-2.006409682	1.10E-17
Rab27b	287.823579	-2.637268501	2.92E-12
Rab34	121.689988	1.857661458	1.03E-09
Rab37	1339.18276	-2.356562325	9.79E-28
Rabggtb	1852.3467	1.200240426	2.24E-23
Rabl2	124.019961	1.145472948	1.17E-06
Rad51	2153.76041	1.57941126	1.10E-10
Rad51ap1	1177.91548	1.241246989	2.37E-07
Rad51b	10837.5383	1.129495796	0.00201378
Rad54b	1350.93981	1.445574152	8.58E-07
Rad54l	1671.74064	1.530349379	1.73E-13
Raet1e	467.167198	-1.078409403	0.00081323
Rai14	249.254246	2.948868942	2.00E-25
Ralyl	214.477885	2.671115997	1.88E-18
Ramp3	186.516496	2.189893996	0.00066036
Ran	9532.99457	1.395795572	7.38E-08
Ranbp1	4229.24944	1.4420607	7.45E-10

Ranbp3l	144.9159	-1.49905954	7.97E-07
Rangap1	6984.18811	1.225864725	6.26E-09
Rap1gap2	3366.94074	-4.123832943	8.25E-58
Rapgef2	5395.82929	-1.103708426	4.40E-07
Rapgef4	4355.66946	-3.125758764	4.53E-19
Rapgef5	262.030054	1.268241723	1.74E-05
Raph1	2174.94994	2.668602306	7.19E-37
Rarb	279.174726	-1.132724195	0.00052206
Rarg	1263.77601	-2.05030185	9.16E-08
Rasa3	17879.3884	-2.392186023	5.10E-80
Rasal2	404.338139	2.897226398	2.21E-14
Rasal3	7549.92972	-1.066127701	3.77E-09
Rasgef1b	1079.06631	1.688334077	4.10E-12
Rasgrf2	653.250674	-1.715940222	7.69E-10
Rasgrp1	18476.4201	-1.29201949	1.47E-10
Rasgrp2	3405.70426	-3.192012812	7.12E-44
Rassf2	4968.66384	-1.309816855	3.45E-19
Raver2	1207.44894	-1.308299156	2.70E-08
Rbm20	405.606075	-1.296506732	3.65E-15
Rbms1	8699.44199	-1.362240788	8.20E-18
Rbpj	12741.6694	2.73355089	3.60E-42
Rcbtb2	1425.76612	-2.14242239	1.24E-54
Rcc1	2504.11917	1.425532301	6.41E-12
Rcc2	11332.5666	1.145342508	1.08E-07
Rcn3	448.8023	-1.353662827	4.50E-09
Rdh11	1547.84826	1.114900966	7.65E-12
Rdh5	108.162181	-1.214547661	1.39E-09
Rec114	1100.1243	-1.100914908	5.76E-05
Reck	739.917099	-1.121106125	1.94E-10
Recql4	640.966291	1.933157095	1.34E-18
Rel	4633.77293	1.048214673	1.70E-08
Rem2	117.520745	1.383943659	2.93E-13
Rere	7028.24761	-1.247674523	1.19E-05
Ret	98.0137474	-1.998879302	6.42E-09
Rfc2	2720.45016	1.022464063	1.84E-08
Rfc3	982.59324	1.430430114	3.08E-09
Rfc4	1446.47828	1.002659252	0.0010934
Rfc5	2284.65044	1.014157293	3.04E-06
Rflnb	500.614936	-1.851884751	1.37E-14
Rfx2	539.204846	-1.81957244	4.82E-14
Rfx3	5771.37189	-1.229436064	0.00012666
Rfx8	101.883296	1.016327721	9.38E-05
Rgcc	1216.89288	1.072540651	0.00056405
Rgs1	1308.10292	1.508948762	0.00060633
Rgs10	2176.04663	-1.136428999	2.67E-15
Rgs16	552.318608	3.511181862	8.64E-45
Rgs2	629.80911	1.333670288	0.02693988
Rgs8	129.585623	2.052968131	1.40E-12
Rhob	269.517519	1.697285138	2.82E-07
Rhoc	346.70479	-1.06387171	7.00E-09

Ric8b	4450.45281	-1.063948782	5.91E-05
Rims2	136.044377	1.694797373	0.000153
Ripor2	24796.9307	-2.561084263	3.00E-38
Ripor3	249.050892	-1.272772397	2.25E-06
Rn7sk	442112.954	-1.477254334	0.00029667
Rnasel	2408.86403	-1.291266922	5.09E-20
Rnaset2b	1299.76371	-1.227947365	7.10E-13
Rnf122	620.039007	-2.132476081	1.41E-31
Rnf126	1742.38963	1.205944392	7.70E-06
Rnf144a	1726.98326	-3.514225707	3.80E-58
Rnf157	5772.07687	1.18242284	9.04E-08
Rnf167	2335.64193	-1.478919858	6.54E-28
Rnu12	392.426076	-1.314722234	0.0011879
Rom1	373.148549	-1.230332056	3.08E-08
Rpa3	866.557281	1.106746129	0.00010658
Rpn1	6030.69223	1.035225953	3.36E-05
Rps11	342.219621	-2.760841721	2.47E-34
Rps19bp1	430.491481	1.096164934	7.49E-08
Rps27	29828.6988	-1.098583154	0.00114186
Rps27l	2459.5911	1.324303812	0.00042922
Rps6ka2	319.348452	-1.622407367	4.10E-13
Rras2	13087.973	-1.114416897	2.41E-11
Rrm1	9464.97918	1.497721257	7.60E-07
Rrm2	6070.29322	1.121778533	0.00044397
Rsrp1	8414.80918	-1.050042097	6.15E-05
Rtn4rl1	1204.51297	-2.81249501	1.31E-29
Rtp4	455.110565	-2.066477354	2.87E-19
Rundc3b	1552.53137	-1.022034245	8.58E-07
Runx1	6687.43974	-1.698143856	9.84E-06
Ruvbl1	2070.37871	1.075601909	6.21E-19
Ruvbl2	2667.10952	1.585469216	6.67E-12
Rxra	968.79414	-1.492520534	2.47E-09
Ryk	709.056836	2.022529625	6.47E-33
Ryr3	543.230259	-1.235821263	1.67E-10
S100a4	207.656662	-1.736968831	0.0066326
S100a6	1491.54986	-2.697388884	3.95E-06
S1pr1	5885.27281	-4.436957572	9.46E-101
S1pr4	2696.6303	-1.650151573	1.40E-07
Samd3	565.286963	-1.145152096	0.00430932
Samd9l	4632.73593	-1.931035245	1.59E-36
Samhd1	20701.294	-1.985413694	4.23E-48
Samsn1	2005.14792	1.010038333	2.20E-07
Sap30	760.30368	1.636324257	1.81E-06
Saraf	9197.26107	-1.30391113	7.53E-12
Sarm1	109.384729	-1.262537942	1.72E-09
Sbf2	247.342392	1.07006863	0.00053406
Sbk1	1470.10009	-1.112032724	5.49E-06
Sbk2	264.578719	-2.140481266	4.02E-13
Scamp1	488.000835	1.64317324	1.35E-22
Scarb2	5051.82612	-1.202726901	4.55E-31

Scarf1	185.213546	-1.691412057	5.21E-07
Sccpdh	140.322146	1.801175722	1.89E-16
Scd1	2100.65026	3.481016981	1.18E-09
Scd2	15573.4017	1.630845208	1.01E-08
Scd4	123.492688	-1.192791174	1.58E-07
Scml4	10024.6111	-2.16016005	1.17E-12
Sco1	1164.24816	1.189644437	4.43E-16
Scrib	1809.20653	1.224321009	1.65E-08
Sdc1	621.908362	4.403511426	1.10E-44
Sdf2l1	1151.9444	1.681697401	6.47E-06
Sdk1	546.307998	-2.621812487	1.74E-15
Sec22c	1140.71123	-1.007759877	1.70E-06
Sec24a	6089.22557	-1.19720414	8.69E-09
Sec31b	231.6492	-2.020211629	3.98E-11
Selenop	1726.37901	-2.068525997	1.61E-32
Sell	18918.395	-1.709862508	1.72E-43
Sema4c	479.41911	3.055068073	2.02E-26
Sema4f	2068.90474	-2.08654638	6.32E-27
Sema6d	479.529725	3.324054792	2.09E-15
Sema7a	534.788091	2.618784823	4.14E-21
Senp7	3978.25332	-1.280655127	1.11E-05
Sephs2	1424.10076	1.074790592	4.75E-05
Sept11	13360.2065	1.412103518	8.69E-22
Sept8	391.828361	-1.947846298	3.86E-18
Serf1	113.61028	1.02401304	1.16E-05
Serpina3g	1259.67368	-1.316330136	3.76E-09
Serpinb1a	182.754159	-2.33163378	1.93E-15
Serpinb6a	1653.1592	1.00486942	1.29E-08
Serpinb6b	1485.64404	2.286157861	1.14E-10
Serpinb9	1406.26071	2.012044814	3.28E-14
Serpine1	174.730514	2.564460178	1.71E-13
Serpine2	1719.23997	5.075930433	3.17E-83
Serpinf1	189.968442	2.072598198	4.39E-20
Serpini1	1364.62837	-2.424867399	1.41E-24
Sesn2	569.950498	1.610968433	9.86E-08
Sestd1	922.125552	2.630695354	1.54E-64
Set	15039.0087	1.210844396	2.62E-08
Sez6l2	147.893497	-1.024049198	1.65E-06
Sf3a3	2724.24934	1.048778481	4.93E-10
Sf3b4	1809.15294	1.053881035	1.95E-05
Sfn	1315.07562	-1.244799105	2.19E-07
Sfrp2	489.290299	-1.542416049	2.64E-07
Sfxn3	1825.30363	-1.517648671	4.24E-19
Sgk1	766.035633	-1.14162566	0.00303502
Sgk3	1777.70013	-2.433179918	5.06E-25
Sgsh	863.182362	-1.372618609	3.07E-14
Sh2b2	114.225435	1.023315815	4.13E-06
Sh2d3c	4000.14072	-1.099202698	8.50E-12
Sh3bp5	7319.21533	-3.318250071	5.11E-72
Sh3gl3	86.8091966	1.098985199	7.39E-05

Sh3pxd2a	1371.42822	-2.100584736	1.25E-27
Sh3rf1	2167.62091	1.402950271	2.89E-12
Shcbp1	1885.18532	1.041044895	0.00092083
She	725.989785	-3.174940985	3.80E-48
Shisa3	117.65709	-1.046903186	0.00017348
Shmt1	2283.73963	1.499131403	3.73E-32
Shmt2	4884.74913	1.736580851	6.02E-11
Shpk	372.067058	-1.268097802	2.28E-09
Siah1a	2662.20272	-1.383675559	2.40E-10
Sidt1	10637.4217	-2.640218435	3.39E-19
Sike1	2477.33325	-1.619737938	8.48E-26
Sit1	771.744133	-1.485785342	3.02E-06
Ska1	717.962483	1.150380258	6.17E-07
Ska3	955.154264	1.112351226	3.66E-06
Skap1	33572.3079	-1.112482143	1.57E-05
Sla2	3393.14417	-1.404236184	6.18E-13
Slamf6	3310.96849	-1.581076154	6.08E-07
Slamf7	1170.02037	-1.284549414	3.64E-08
Slc12a2	1190.67081	1.07652835	1.96E-10
Slc12a4	752.583564	1.150739593	6.13E-10
Slc12a7	7784.12201	-2.53189972	5.23E-32
Slc12a8	245.51959	1.7245469	1.29E-11
Slc14a1	2472.15471	-3.531754414	3.28E-26
Slc15a1	179.772039	2.192436224	1.07E-12
Slc15a2	646.232642	-2.233488662	1.51E-09
Slc15a3	102.803262	1.298226401	1.73E-07
Slc16a10	1877.91868	1.629536675	4.77E-21
Slc16a11	196.230057	1.364278357	4.40E-09
Slc16a2	124.349034	-1.25435082	1.76E-06
Slc16a3	3580.03933	2.193073148	3.32E-07
Slc16a5	549.415715	-2.546699131	1.26E-33
Slc17a6	205.980597	2.901544799	2.94E-27
Slc17a9	2040.50002	-1.63166481	6.33E-11
Slc19a2	592.759424	1.192299828	2.24E-11
Slc1a2	227.321778	3.024540775	1.83E-34
Slc1a4	418.503271	3.381293816	6.18E-34
Slc22a15	616.877663	1.523147293	1.77E-08
Slc25a13	2121.87932	1.462332681	2.74E-11
Slc25a18	566.370542	-1.204191529	2.97E-10
Slc25a45	888.267665	-2.160901944	3.73E-44
Slc25a5	10984.504	1.069166369	1.15E-05
Slc25a53	1375.34365	-1.178300002	1.32E-08
Slc26a11	627.43266	-1.284259887	6.12E-09
Slc28a2	6497.4946	-1.612113461	1.34E-12
Slc29a1	2123.08059	1.26303619	3.75E-10
Slc2a3	5561.84702	1.16786323	3.85E-07
Slc2a4rg-ps	777.877807	-1.122530372	2.40E-05
Slc2a9	1812.93803	-1.775378091	3.35E-10
Slc30a4	5753.86963	-1.860386274	1.67E-24
Slc35d3	124.999	1.635028387	5.24E-07

Slc35f5	169.776709	1.46495363	2.00E-16
Slc35g1	3427.93827	-1.214738148	2.97E-11
Slc35g2	123.644217	-1.588486972	1.75E-07
Slc36a3	195.208799	-1.06404868	0.0056841
Slc37a2	343.322656	1.592061834	1.62E-33
Slc39a10	1607.1627	1.321691352	5.49E-19
Slc39a4	334.320267	1.286838967	2.27E-06
Slc3a2	4218.83057	1.002222055	2.63E-06
Slc43a1	445.520981	-1.513073115	7.46E-11
Slc43a2	3648.87114	-1.657355587	5.85E-24
Slc43a3	1124.85351	1.537981144	6.84E-11
Slc46a3	645.489257	-1.02811806	1.29E-16
Slc4a10	200.054397	-1.75600215	2.26E-13
Slc4a11	170.831277	1.02183993	2.14E-06
Slc4a4	306.190992	-2.270171375	1.21E-20
Slc5a10	222.595272	-1.222328689	4.13E-11
Slc6a19	552.644548	-2.498450241	5.68E-34
Slc7a1	9174.37846	1.144533391	8.94E-14
Slc7a3	172.165525	2.451291179	1.62E-17
Slc9a5	607.856194	1.5473001	6.62E-12
Slc9a7	1124.6895	-1.378110292	0.00010485
Slc9a9	14081.5723	-1.724611922	1.33E-08
Slc9b2	271.856364	2.488474748	1.80E-47
Slco3a1	15518.779	-2.988810893	1.20E-34
Slco4a1	1645.64289	1.795685061	2.36E-13
Slfn1	2442.29027	-1.81988658	3.87E-14
Slfn2	1054.15217	-1.015226867	0.00013094
Slfn9	1256.2152	1.215835634	1.88E-05
Slpi	118.742675	-1.903792292	3.46E-12
Slurp1	135.651585	-1.02488672	3.91E-10
Smad3	4193.25885	-3.669574402	2.28E-41
Smad7	3729.46082	-1.438735483	7.09E-20
Smap2	8594.01843	-1.377034854	1.50E-26
Smarca2	6670.68198	-1.185842499	2.46E-08
Smc2	6795.68609	1.122062195	5.72E-05
Smim24	200.727646	-1.597356133	1.05E-19
Smo	323.995209	1.953468099	2.86E-08
Smpd4	2071.39745	1.288198594	1.81E-18
Smpdl3a	1084.45039	-1.693470124	2.11E-13
Sms	3594.78397	1.059122539	9.27E-23
Smurf1	3744.10687	-1.232427527	2.36E-08
Smyd5	1803.12738	1.957592336	9.63E-46
Snrpd2	1862.65465	1.027352439	0.00062477
Sntb1	7179.88951	-2.91521861	9.66E-15
Sntb2	3542.87473	-1.976468514	4.95E-24
Sntg2	290.847887	3.499343668	7.71E-25
Snx24	94.1929884	1.149549169	3.40E-08
Snx29	3715.62165	-1.080107443	2.78E-05
Snx8	370.156142	1.388216068	6.00E-22
Sobp	115.346238	1.442483724	1.91E-11

Socs3	1712.01781	-1.845405701	1.79E-15
Soga1	132.451697	1.235769216	3.61E-09
Sorbs1	3066.26636	-1.431696468	5.72E-07
Sorcs1	760.288366	4.750982778	4.27E-93
Sorcs2	2161.41588	-5.089620595	7.59E-95
Sorl1	7861.44459	-2.13997282	2.09E-60
Sort1	127.262855	1.449150591	2.67E-15
Sp100	14162.4608	-1.439228653	2.76E-18
Sp4	5118.9555	-1.219613217	1.59E-08
Spag5	2602.05039	1.109393601	0.00010795
Spata1	472.80418	-1.076726962	5.39E-05
Spata24	405.095532	1.036060722	3.05E-06
Spata6	3018.45211	-1.169268657	5.58E-07
Spata7	242.822196	1.789104248	1.16E-16
Spats2	427.912439	1.414708541	1.02E-06
Spc24	1070.8304	1.178981146	2.24E-05
Spcs3	3727.1197	1.158453881	6.47E-08
Spdl1	1205.26397	1.348796833	2.24E-10
Spef2	727.366807	1.674919591	2.00E-10
Spin4	336.003702	3.233205484	1.84E-40
Spink13	263.77849	-2.889592446	4.01E-14
Spire1	704.433931	2.158227691	7.50E-86
Spock2	373.646963	-1.15064419	0.00083679
Spon1	2038.94033	-1.81148141	1.05E-15
Spp1	462.059852	4.171115718	3.05E-68
Spred1	1217.26434	2.230083499	1.11E-31
Spred2	4780.9486	1.326911208	3.43E-10
Spry1	441.798458	1.404093058	1.43E-06
Spry2	614.370323	3.635871464	1.07E-74
Sqle	2544.30372	1.11889729	8.34E-06
Srd5a3	875.789603	-1.03958393	9.17E-16
Srgap3	6185.97786	1.071156444	4.70E-06
Srm	1846.81718	1.683213493	2.21E-10
Srsf9	1692.19344	1.132870214	4.46E-11
Srxn1	102.297963	1.372191101	1.51E-08
Ssbp2	11613.2729	-1.818882033	3.48E-08
Ssh2	15303.351	-1.038960241	3.30E-08
Ssr2	2690.78952	1.283225939	5.66E-07
Ssrp1	9344.18872	1.000050349	4.83E-05
Ssx2ip	587.354058	1.617328287	4.24E-29
St13	5016.15165	1.459822422	2.27E-28
St14	1225.75303	2.352602102	2.11E-25
St3gal1	2710.61665	-2.286614964	9.65E-30
St3gal2	442.839364	1.822535042	1.71E-44
St3gal6	1165.18498	-1.208471107	8.58E-05
St8sia1	3429.80509	-2.344178216	1.24E-09
Stard13	89.527807	1.038137143	5.11E-06
Stard5	856.420913	-1.12987996	2.20E-15
Stat1	3182.99704	-1.371798689	3.14E-15
Stat3	13649.2679	1.705152971	2.36E-14

Stau2	211.570431	1.967764717	9.84E-20
Stil	2877.26298	1.053103935	4.74E-05
Stim1	9516.90353	-1.071848641	6.81E-07
Stip1	5743.41193	1.30590864	7.17E-10
Stk32c	268.936076	1.664138579	9.90E-14
Stk38	7637.14684	-1.542040656	1.30E-13
Stk38l	2501.17669	1.134881669	5.80E-07
Stk39	16724.3654	1.458139118	1.69E-10
Stkld1	320.062497	-1.021649825	7.48E-11
Stom	126.892421	1.114964263	2.33E-06
Stoml2	1923.37428	1.291119622	6.24E-07
Ston1	284.589953	1.922533861	4.52E-14
Ston2	615.635432	2.533110717	1.59E-18
Stx11	1303.53398	1.708712775	3.37E-10
Stx1a	1234.57246	-1.155878156	0.00043275
Stx2	1363.53513	-1.678573826	3.52E-12
Stxbp1	272.483338	1.528878647	9.79E-11
Styk1	164.51139	1.221500347	0.00010903
Styxl1	125.185745	-1.074421883	0.00037765
Sugct	346.09776	-1.104557863	0.00054401
Sun2	8659.37432	-1.020833278	1.23E-06
Syce2	671.972807	1.01240567	1.81E-05
Syne1	5521.61996	-1.886971731	4.75E-29
Syne2	1796.24491	-1.636595205	8.83E-36
Syngr1	162.704837	1.684585639	2.87E-14
Synpo	1811.72008	1.378304711	8.86E-38
Sypl	463.788784	1.306590178	2.76E-11
Syt6	1750.6776	-4.758720274	3.42E-17
Sytl1	785.685878	-1.896750263	2.53E-22
Sytl3	2382.84409	-1.097246664	2.24E-05
Tacc3	2990.4746	1.205074932	2.50E-06
Tagln2	9937.36158	1.173699548	5.76E-05
Tanc1	5643.82698	-1.326765503	9.46E-13
Tanc2	1451.46081	1.021672643	1.37E-05
Tars	3105.44174	1.305643053	4.16E-17
Tbc1d10c	6407.9104	-1.283986139	1.57E-14
Tbc1d2	209.920856	-1.07527366	1.76E-05
Tbc1d30	248.199832	1.048278384	1.08E-05
Tbc1d31	1478.73801	1.419144015	6.77E-10
Tbc1d7	406.568422	1.0715575	9.65E-17
Tbcel	3677.66469	-1.216063899	3.31E-09
Tbl2	1269.07957	1.126105044	1.65E-08
Tbl3	1317.16003	1.185874528	6.91E-10
Tbx6	236.167569	-1.267424739	3.47E-09
Tbxa2r	1096.40158	-1.157415266	1.29E-07
Tbxas1	109.365521	-1.079112973	1.45E-05
Tcf4	1157.6918	1.292386902	8.82E-09
Tcf7	1330.44459	-2.716813292	6.60E-13
Tcp1	8666.69461	1.369639343	4.79E-11
Tcp11	131.997555	-1.063153362	4.26E-09

Tcp11l2	1868.95905	-3.517774812	7.72E-80
Tcrg-C1	573.643776	-2.641750567	7.00E-14
Tcrg-C2	419.017379	-1.543921844	4.53E-06
Tcrg-C4	320.474828	-1.674205841	5.69E-05
Tctex1d1	165.992397	-1.411448388	0.00015415
Tctex1d2	111.923887	1.281036339	2.66E-09
Tdo2	220.866001	-1.781501686	2.45E-14
Tdrd7	222.69332	1.553020053	8.97E-14
Tdrkh	246.260115	1.624146592	9.87E-13
Tdrp	1369.86288	-2.459157939	5.55E-14
Tead2	245.859027	-1.609563422	7.43E-14
Tecpr1	6717.39753	-1.172699133	4.99E-11
Teddm2	195.334242	-1.938254985	9.12E-13
Tenm4	118.297584	1.37045313	1.07E-12
Terc	980.829002	-1.018096887	0.02358094
Tert	202.212233	1.112640727	3.16E-07
Tet1	330.549927	-1.131482154	8.85E-07
Tex15	414.744459	2.17922185	5.37E-18
Tex45	155.674371	-1.601414664	2.45E-05
Tfdp1	7861.23655	1.248822725	3.66E-19
Tfrc	19803.8741	1.801692762	1.06E-13
Tg	2320.45847	1.675756699	3.47E-12
Tgfb1	5095.71278	1.028741204	2.72E-06
Tgfb1i1	119.545261	-1.296480417	6.31E-07
Tgfbr1	2686.48845	-1.045770309	0.0001297
Tgfbr2	11340.5957	-1.345574473	2.01E-24
Tha1	285.780739	-2.253463605	9.67E-46
Thap3	956.5961	-1.672964668	3.53E-13
Thop1	1063.58362	2.168154021	1.07E-16
Thra	965.908713	-1.230896903	1.98E-06
Thumpd2	951.407581	-1.050842733	5.77E-06
Ticam2	135.672449	1.049876537	1.26E-06
Ticrr	2786.82331	1.545017195	3.63E-09
Tigit	2003.97747	3.056802351	1.03E-37
Timeless	2597.21497	1.278098626	9.33E-09
Timm13	885.909896	1.159862422	8.92E-05
Timm50	1218.16037	1.659167093	2.37E-11
Timp2	1524.35021	-1.937624551	3.30E-13
Tipin	2466.11775	1.237581245	2.04E-11
Tirap	289.108343	1.337392445	1.54E-07
Tjp2	928.316686	1.495858988	1.51E-09
Tk2	1618.41038	-1.655982639	1.59E-17
Tlcd2	139.192769	1.827556761	1.17E-13
Tldc2	352.636625	-1.969411562	3.04E-32
Tln2	336.10382	2.285740051	8.23E-35
Tlr1	1848.47131	-3.009020612	2.01E-73
Tlr12	183.018125	-2.231024798	3.02E-29
Tlr6	208.30712	-1.318970473	1.65E-12
Tmem106c	407.594634	1.067459021	1.63E-05
Tmem108	497.48425	3.855513608	2.28E-50

Tmem109	1683.61972	1.673902909	8.24E-12
Tmem132b	109.686895	-1.312317148	4.81E-06
Tmem140	255.258462	-1.507116977	5.65E-11
Tmem163	4432.35007	2.088520469	7.45E-10
Tmem176a	160.253292	-1.468068833	2.24E-05
Tmem176b	170.309606	-1.510497219	1.73E-05
Tmem2	933.623256	1.07219846	1.80E-06
Tmem221	288.259345	-1.533730348	2.04E-12
Tmem237	374.636495	1.228969369	2.41E-12
Tmem37	98.6597536	-1.191265483	2.31E-08
Tmem50b	851.733771	-1.152492626	8.36E-24
Tmem63a	1010.52197	-1.082938515	5.58E-13
Tmem67	289.078081	1.230552338	3.97E-14
Tmem71	4362.84315	-2.818746009	1.48E-69
Tmem97	1262.14458	1.528609541	2.67E-11
Tmod1	258.166488	1.791319647	2.79E-23
Tmod4	123.239898	-1.064590764	5.14E-11
Tmtc3	345.324987	1.374048149	1.47E-07
Tnc	97.2514503	1.439438883	8.67E-08
Tnfaip1	987.161009	1.000757587	1.01E-15
Tnfaip8l1	844.349728	-1.26360324	4.81E-21
Tnfaip8l2	1155.76117	-1.320077727	1.17E-08
Tnfrsf11a	221.677923	-2.063057376	1.55E-15
Tnfrsf11b	88.4085527	1.261532541	2.69E-06
Tnfrsf14	1445.76651	-1.378680755	1.82E-12
Tnfrsf25	139.413445	-1.287672721	6.13E-06
Tnfrsf26	1849.86294	-2.52279652	5.25E-69
Tnfrsf4	2284.37047	3.70266619	1.88E-53
Tnfsf11	717.835004	3.357379704	2.85E-26
Tnfsf4	233.105166	2.283541131	1.18E-15
Tnfsf8	6286.52152	-1.206299499	1.54E-06
Tnfsf9	118.26649	1.01750016	1.12E-07
Tnk2	1393.42903	1.279962182	3.91E-06
Tnn	258.937284	-1.622527793	8.38E-06
Tnnt3	178.824985	-1.769122295	5.18E-20
Tns1	1624.81172	1.21744632	1.24E-06
Tns3	117.318418	1.491308378	4.15E-11
Tomm40	2481.86781	1.296522515	2.35E-12
Tox	11556.9665	1.557806796	4.11E-06
Tpbgl	102.159888	-1.23071809	6.28E-08
Tpcn2	1828.06137	-1.683666074	5.26E-07
Tpi1	16559.3598	1.69324456	0.00013449
Tpmt	189.53206	1.127236994	2.89E-12
Tpst2	4600.84	-1.454188475	9.20E-16
Tpx2	4971.32834	1.185615343	3.33E-05
Traf3ip3	6722.862	-1.187539661	5.87E-17
Traf4	1659.87605	1.094520409	8.18E-06
Traip	1040.83879	1.294957246	2.43E-10
Tram2	1429.04834	-1.17794857	1.02E-10
Trat1	455.236045	-1.515258212	1.44E-10

Trav14-1	263.713801	-1.204694492	6.16E-06
Trav15-2-dv6-2	172.054012	-1.093193364	5.40E-07
Trbj1-1	798.425151	-1.023908878	2.35E-10
Trdc	505.5109	-1.176206106	1.85E-10
Trib2	2300.74807	-1.290538921	2.28E-15
Trim12a	3150.91086	-1.466456711	8.61E-12
Trim15	174.762977	-1.030930706	1.51E-09
Trim28	8267.03747	1.034680575	0.00011052
Trim30c	434.235263	-3.298643413	2.30E-29
Trim47	148.411481	-1.332522606	5.52E-07
Trim5	1284.29487	-1.407142038	7.22E-15
Trim65	304.470057	-1.085460953	1.09E-09
Trip13	1421.4728	2.011919412	5.49E-19
Troap	645.123422	1.057638058	7.45E-05
Trp53cor1	137.24016	1.614070309	7.21E-10
Trp53inp1	5800.198	-1.388579056	2.00E-13
Tsc22d3	2046.37456	-1.532260086	3.25E-11
Tspan13	4384.41509	-1.239675977	8.13E-26
Tspan2	1256.58651	1.014080414	1.27E-05
Tspan6	139.613764	1.610387549	8.62E-14
Tspan9	122.099091	-1.172283409	0.00081831
Ttc17	4100.75551	-1.040952373	1.05E-06
Ttc26	118.017312	1.615195796	8.50E-16
Ttc38	1083.95581	-1.495613534	1.40E-26
Ttc39c	1948.0114	4.645748346	2.39E-54
Ttc7	6783.32094	-1.036283469	3.88E-08
Ttk	1317.31545	1.159323535	9.50E-06
Ttyh3	2820.58011	-1.457728759	3.91E-15
Tuba1b	11787.2214	1.378410785	0.00020246
Tuba1c	3107.72138	1.179857894	0.00017251
Tubb2a	329.772729	1.004769817	0.000333
Tubb2b	176.674084	1.476811973	6.25E-09
Tubb4b	6269.32948	1.275544503	0.00022631
Tubb6	1303.69409	3.133914723	5.74E-28
Tube1	614.731415	1.22010937	6.18E-10
Tubg1	1265.13938	1.465758401	9.57E-08
Tufm	1666.7358	1.411459178	1.38E-08
Twf2	2719.2369	-1.039002146	1.19E-07
Twist2	171.10528	2.595617757	7.09E-35
Twsg1	2382.73598	2.098599868	1.78E-71
Txk	7742.13798	-2.514810688	3.84E-28
Txn1	5914.99438	1.246898051	1.24E-06
Tyrobp	101.641187	-1.337822847	2.27E-07
Uba7	1779.77452	-1.509014278	6.33E-12
Ubald1	1832.20539	-1.152573374	3.23E-11
Ubash3b	12594.3066	1.18071899	7.70E-09
Ube2c	2178.98498	1.023657808	0.00354072
Ube2e2	423.074085	2.119231806	1.94E-16
Ube2h	9915.40684	-1.226797532	3.20E-10
Ube2m	2825.16964	1.220319473	1.53E-07

Ubqln4	1818.42093	1.209974119	6.30E-12
Ubtd1	837.219437	-1.205728753	0.00013268
Uchl5	2184.29931	1.11128704	1.28E-07
Uhrf1	9453.14895	1.64272603	1.33E-07
Uhrf1bp1	776.87649	1.27421099	6.87E-11
Umps	2059.70574	1.391089822	1.38E-10
Unc119	385.051393	1.010851074	0.00037799
Unc5a	183.212878	-1.559304501	9.68E-13
Unc5cl	1807.96032	-2.039640259	8.19E-07
Ung	1085.14296	1.356101987	9.25E-09
Upp1	146.541986	1.993733872	1.01E-10
Uqcr10	1750.6351	1.102948135	0.00035342
Uqcrq	1891.26699	1.13393752	0.00040476
Use1	1723.5184	-1.042646076	0.0001778
Usp20	1139.36917	-1.020049302	1.71E-13
Usp28	6147.32797	-2.01585978	5.91E-13
Usp3	14454.9534	-1.747454501	2.49E-18
Usp50	270.662999	-1.115412515	4.23E-12
Usp6nl	2071.0444	1.183593125	1.52E-05
Ust	9082.39353	-1.424864643	3.91E-06
Utp11	1276.56884	1.076892801	5.31E-21
Utp20	4678.33136	1.014886655	3.74E-12
Utrn	35605.6445	-1.31180473	2.73E-08
Vash1	134.378784	1.191046166	1.64E-07
Vat1	1118.40684	1.041138975	2.94E-06
Vav2	1597.71382	1.491919247	5.56E-10
Vcan	163.362358	1.153113863	2.60E-08
Vcl	1298.00568	1.220674818	3.99E-08
Vdac1	5735.05355	1.092338493	1.65E-08
Vegfa	888.162505	1.117414142	0.01198287
Vipr1	961.114915	-3.436004096	1.73E-65
Vldlr	159.608153	1.628967767	0.00012199
Vmac	389.396593	-1.290081953	6.68E-09
Vmn2r96	278.41662	-1.69345204	2.14E-07
Vmn2r97	114.649979	-1.108000184	7.40E-05
Vrk3	1743.57767	-1.683183038	5.53E-29
Vsig2	154.725088	2.023748349	7.04E-17
Vwa3b	312.199734	-2.968112341	6.87E-30
Vwa5a	1738.12363	-1.446333331	7.05E-15
Vwa8	4072.28829	1.056890297	8.01E-05
Wdfy2	829.832232	1.036229097	6.99E-11
Wdhd1	3389.77045	1.433337041	2.74E-13
Wdpcp	615.391296	1.088819004	0.00020754
Wdr18	1491.42434	1.372993476	9.93E-15
Wdr31	110.218831	1.134355965	5.87E-08
Wdr35	344.763711	2.112731552	6.95E-34
Wdr37	5546.37862	-1.020956818	7.01E-05
Wdr41	832.789679	-1.18322254	1.34E-05
Wdr49	168.092506	-1.386923977	2.20E-07
Wdr54	105.43829	1.048884977	0.0001178

Wdr60	215.335724	2.189688839	2.62E-50
Wdr62	1064.9845	1.189765022	8.04E-06
Wdr66	150.525322	-1.185863139	1.63E-06
Wdr76	2990.63661	1.05229435	9.12E-07
Wdr78	892.616968	-1.138933089	1.47E-05
Wdr86	180.81292	-1.332166358	3.38E-12
Wdr95	204.954659	-2.065367684	1.33E-09
Wnk3	191.545726	-1.062273804	0.0017177
Wnt10b	436.063262	3.436232417	1.80E-47
Xbp1	2329.0352	1.370453515	2.14E-06
Xkr5	334.910512	1.118517963	6.06E-07
Xpot	3864.01643	1.280116388	2.27E-27
Xrra1	219.291454	-1.034113574	1.47E-07
Yap1	144.345003	1.557603214	1.18E-19
Yars	3014.90448	1.496837374	1.31E-25
Ybx1	14482.4541	1.637367192	1.46E-12
Ybx3	8615.30496	1.733783386	6.72E-23
Ypel1	563.44189	-1.572524695	4.12E-14
Ypel2	866.976248	2.077999358	2.64E-22
Ypel3	1408.71087	-2.189405468	4.50E-23
Ypel5	1603.45684	-1.313690846	1.97E-13
Zan	195.469518	1.499549061	1.11E-08
Zbp1	1941.61346	-1.649836454	3.50E-12
Zbtb10	257.108331	-1.140220154	9.45E-08
Zbtb20	20200.6993	-1.415307045	5.42E-05
Zbtb32	2300.139	4.230201795	1.07E-85
Zbtb4	1961.2313	-2.088037341	5.38E-23
Zbtb7b	424.75006	1.598386688	5.40E-06
Zbtb8a	439.197845	-1.088249273	2.79E-13
Zc3h12a	2008.14979	-1.389644428	2.88E-08
Zc3h12c	100.148234	1.250434157	5.00E-11
Zc3h6	1927.07965	-1.577518213	0.00051642
Zdhhc15	2025.87262	-1.243844073	2.27E-05
Zdhhc17	2055.07023	-1.283026452	1.70E-07
Zdhhc19	146.567533	-1.159810055	1.52E-07
Zdhhc2	1170.98791	1.144440354	1.99E-06
Zeb2	954.440628	3.096862327	5.10E-35
Zfand4	530.101024	1.051282993	8.06E-05
Zfp12	941.458963	-1.028203273	2.70E-06
Zfp219	1419.41447	1.851142555	8.42E-26
Zfp260	1757.22765	-1.0954164	5.16E-09
Zfp280b	3439.82876	1.815336187	1.49E-20
Zfp36	2519.49832	-1.48027565	4.87E-07
Zfp362	1686.93057	-1.099783083	1.53E-07
Zfp386	2481.33773	-1.950946439	3.20E-26
Zfp41	429.369351	1.024273834	2.24E-06
Zfp429	212.297026	-1.080032709	7.70E-08
Zfp455	233.797461	-1.080449123	2.37E-06
Zfp518b	175.180346	1.004725986	1.49E-07
Zfp52	1049.81408	1.693307071	5.58E-13

Zfp608	3303.22426	1.432178901	8.29E-07
Zfp652	5703.41057	-1.929393652	1.01E-19
Zfp710	1329.03011	-1.229904583	1.74E-07
Zfp768	145.919021	1.658316982	1.23E-13
Zfp773	230.539564	-1.194074287	9.78E-05
Zfp831	3073.29587	-1.231769353	5.96E-05
Zfp862-ps	344.424951	1.675557355	7.51E-21
Zfp870	135.845694	1.881968438	3.53E-23
Zfp874b	454.276814	-1.198764743	6.19E-12
Zfp945	1012.7188	-2.492680701	4.97E-21
Zfp948	718.463564	1.652253301	7.96E-06
Zfp976	113.176877	1.28307821	4.75E-07
Zfr2	217.001496	-1.438129588	8.65E-07
Zgrf1	2885.80535	1.157649105	3.54E-06
Zmynd12	136.226969	-1.360375617	1.83E-07
Znrf2	9463.49173	-1.180446505	9.92E-10
Zwilch	1279.41126	1.693535843	8.69E-17
