# Supplementary material for: Rapid in vitro generation of bona fide exhausted CD8+ T cells is accompanied by Tcf7 promotor methylation
Source: PLoS Pathog. 2020 Jun 24;16(6):e1008555. doi: 10.1371/journal.ppat.1008555 (PMC7340326; doi:10.1371/journal.ppat.1008555)
Supplement: S2 Table — Fold change is show for repeat peptide OT-I versus single peptide stimulated OT-I. After 5 day cultures, cells were sorted and MeD-seq was performed. Greater than 2-fold changes shown. (PDF) [file ppat.1008555.s008.pdf]

S2 Table

Differentially methylation of TSS regions was determined by MeD-seq. Fold change is show for repeat peptide OT-I versus single peptide stimulated OT-I. After 5 day cultures, cells were sorted and MeD-seq was performed. Greater than 2-fold changes shown.

Gene name	Fold Change
Haghl	6.10
Ccdc78	6.10
Hic1	5.02
Terf2	4.53
mmu-mir-7075	4.53
Trav13d-2	3.99
Gm22	3.90
Dnajc11	3.81
Rara	3.64
Trim65	3.52
A630014C17Rik	3.30
1700067K01Rik	3.19
Unc80	3.12
Col8a2	3.09
Gm16534	3.09
Plekho1	3.08
Olfr94	3.05
Acp5	3.04
C4b	2.91
Gtf3c2	2.90
Hnf1a	2.87
2310042D19Rik	2.81
Fes	2.79
Gm11695	2.78
Slc16a5	2.78
H2-Ke6	2.77
Cactin	2.77
Gm23547	2.76
Ush1c	2.75
Ttll3	2.73
Hras	2.69
Lrrc56	2.69
Gm26551	2.68
Pnpla1	2.66
P4htm	2.65
Mapk13	2.55
2310011J03Rik	2.53
Hnf1a	2.53
Mef2b	2.51
Gm15932	2.51
Fitm1	2.51
Emc8	2.51
Tmem54	2.49
Tekt4	2.48
Gm15821	2.47

mmu-mir-7005	2.46
Ssh3	2.44
Fam179a	2.44
Gm20492	2.43
Hoga1	2.41
Lnx2	2.41
Polr1d	2.41
Mir219-2	2.39
Zcchc14	2.38
Gm27030	2.38
Bcat2	2.37
Mak16	2.37
Fam83g	2.36
Nfatc2	2.35
Gm24958	2.34
Slc11a1	2.34
Mfrp	2.34
Ncmap	2.34
Raet1e	2.33
H60b	2.33
Gnmt	2.33
Gm24366	2.32
H2-Ke2	2.32
Ccnd3-ps	2.32
Gm10687	2.31
Mcam	2.31
Mrps24	2.31
Pxn	2.31
Tor2a	2.30
Apoe	2.30
Col11a2	2.30
Vps51	2.29
BC048609	2.29
Gm26592	2.29
BC100451	2.29
Gm16069	2.28
Fam189b	2.28
AU023871	2.28
Ly6g6c	2.28
Hras	2.27
Lrrc56	2.27
Gm11959	2.27
mmu-mir-7687	2.27
Caskin2	2.26
Tsen54	2.26
Padi3	2.26
Gm13032	2.26

Rpl18	2.25
Dbndd1	2.25
C920011F04Rik	2.25
mmu-mir-6927	2.24
GNAS-AS1_1	2.24
Ctr9	2.23
Pdxk	2.23
mmu-mir-6913	2.22
Gm13294	2.22
Zfp575	2.20
Ethe1	2.20
Ocrl	2.19
mmu-mir-6996	2.19
Shd	2.18
Grina	2.18
Gm15598	2.17
Tssk3	2.17
Fam229a	2.17
Mmp14	2.17
Efcab4a	2.16
Gm10814	2.15
Gm13784	2.15
Chrng	2.14
Cygb	2.14
Grb7	2.14
Bcl6b	2.14
Ttyh3	2.14
Padi3	2.13
Gm13032	2.13
Hirip3	2.12
C1qtnf5	2.10
Gm20444	2.10
Six5	2.10
Gm26519	2.10
Gm11405	2.09
Bak1	2.09
Sapcd1	2.08
Gm16120	2.08
mmu-mir-8093	2.08
Fam101a	2.08
Cul7	2.08
Arhgap27	2.08
Gm11641	2.08
Kat2a	2.08
Hspb9	2.08
Rab3a	2.08
Hspb1	2.07

Lcat	2.07
Mir219-2	2.07
mmu-mir-7235	2.07
Mir338	2.07
Itpripl2	2.06
Apba3	2.06
Sms	2.06
Gm11537	2.05
Ifitm1	2.05
Ghdc	2.05
Caskin2	2.05
Fam83f	2.05
Lamb2	2.05
n-R5s151	2.04
Gm11959	2.04
Gm20444	2.04
Pde4c	2.03
Kctd17	2.03
Kcnmb1	2.03
Tenc1	2.03
Mir3057	2.03
Gm13826	2.02
Tcrg-V3	2.02
Col11a2	2.02
Celsr1	2.02
Spaca4	2.02
mmu-mir-6939	2.02
Tpsb2	2.02
RP24-312B12.1	2.01
Cyba	2.01
Itgae	2.01
Agap2	2.00
Gm23135	2.00
mmu-mir-7032	2.00
Tcf7	2.00
mmu-mir-6990	2.00
1700120G11Rik	-2.00
Gm9839	-2.01
Gm14458	-2.02
Gm14231	-2.03
Slc37a1	-2.03
Usp7	-2.04
Arhgap28	-2.06
1700080E11Rik	-2.06
Gm14920	-2.07
Cep120	-2.07
1700031F05Rik	-2.07

C230088H06Rik	-2.07
Gm13152	-2.07
Olfr139	-2.07
9330158H04Rik	-2.08
Pam	-2.08
Atic	-2.08
Gm26644	-2.10
Gm20706	-2.10
Casp6	-2.10
Cobll1	-2.11
Gm13594	-2.11
Khdc1b	-2.11
Plaur	-2.11
Ryk	-2.12
Gm13046	-2.12
Mkx	-2.14
Pdgfrl	-2.14
Stxbp1	-2.15
Gm13524	-2.15
Tbc1d4	-2.16
Trav9-1	-2.16
Rpl31	-2.17
Sv2c	-2.17
Gm13967	-2.18
Rfpl4b	-2.19
Gm15164	-2.19
Rpl22-ps1	-2.20
Gm15477	-2.20
Dclk3	-2.20
Pdcd1	-2.20
Fam216a	-2.21
Gpn3	-2.21
Gm24196	-2.22
Tnf	-2.22
App	-2.22
Gm15138	-2.23
Mob2	-2.23
Usp7	-2.23
Sh2d1a	-2.23
Pgm3	-2.23
Rwdd2a	-2.23
Gm20482	-2.24
Gm10742	-2.25
Gm5174	-2.25
n-R5s68	-2.26
Gm24850	-2.26
Lap3	-2.26

Gm13943	-2.27
Zdhhc14	-2.28
Gm23479	-2.28
Ndufs1	-2.28
Eef1b2	-2.28
Clec14a	-2.29
Gm14651	-2.30
Gm4910	-2.31
2410137F16Rik	-2.32
Fbxo41	-2.33
Gm10713	-2.33
Gm15904	-2.34
Rcan2	-2.34
App	-2.35
Itgb6	-2.38
uc_338	-2.38
Gm26457	-2.38
Snora41	-2.38
Ndufs1	-2.39
Eef1b2	-2.39
Arhgef4	-2.40
Unc80	-2.40
Mau2	-2.40
Sugp1	-2.40
Pawr	-2.40
Gm26814	-2.42
Ier3	-2.42
Flot1	-2.42
Prdm6	-2.44
Gm26500	-2.45
Gm40	-2.46
Wdfy1	-2.46
Mrpl44	-2.46
Glra1	-2.46
Gm11608	-2.48
Gm24513	-2.48
Zdbf2	-2.48
Rps6ka2	-2.48
Sh2b1	-2.49
Grb14	-2.50
Cdk13	-2.51
A730020E08Rik	-2.51
Ccser1	-2.51
App	-2.52
Prkce	-2.52
Gm14120	-2.52
Nlrp5	-2.53

Usp46	-2.54
PVT1_5	-2.54
R3hdm4	-2.54
Kiss1r	-2.54
5330438I03Rik	-2.55
Gm24281	-2.56
Gm27003	-2.56
Gm7113	-2.57
Surf4	-2.57
Gm711	-2.57
Gm9946	-2.59
Olfr115	-2.60
Lmbrd1	-2.61
Serinc5	-2.63
Maf	-2.63
Zcrb1	-2.63
Pphln1	-2.63
Gm15762	-2.64
AI646519	-2.65
Pax1	-2.65
Steap1	-2.65
Olfr90	-2.68
Gm11992	-2.68
Taar9	-2.69
Gm13963	-2.70
Tub	-2.70
Rab12	-2.70
Gzmb	-2.71
Mettl25	-2.71
Ccdc59	-2.71
Rpl31	-2.73
Tex13a	-2.74
Prdm6	-2.80
Cckbr	-2.80
Taar9	-2.82
Mboat7	-2.83
Tsen34	-2.83
Gm21411	-2.83
Gm3654	-2.83
8030462N17Rik	-2.83
4930465K10Rik	-2.83
Paxip1	-2.84
Gm24462	-2.85
Tnfrsf4	-2.87
Ston1	-2.87
Tcf7l2	-2.87
Mboat7	-2.88

Tsen34	-2.88
1700015F17Rik	-2.89
Jmy	-2.90
Wnt5a	-2.94
Defa-ps12	-2.95
Sv2c	-2.99
Csnk2a1-ps	-2.99
Fam135b	-2.99
Nxf7	-3.00
Sctr	-3.04
Cdh11	-3.06
Olfr2	-3.08
1700024P16Rik	-3.12
Rad51	-3.14
Dmrt2	-3.14
Sorcs1	-3.14
Cdk13	-3.18
Ccdc43	-3.28
Fras1	-3.29
Tyw5	-3.29
9430016H08Rik	-3.29
Fam207a	-3.34
H2-Q2	-3.37
Lrrtm4	-3.39
Tm7sf2	-3.41
Gm12710	-3.43
Tom1l1	-3.44
Grpr	-3.48
Gm5742	-3.49
Cux1	-3.56
Asb1	-3.58
Edaradd	-3.60
AF357341	-3.67
Mir882	-3.67
Gm22981	-3.67
Gm25856	-3.67
Gm13732	-3.71
Lphn2	-3.78
Ehhadh	-3.81
Gm10742	-3.86
Gm23982	-3.89
Chst9	-3.90
Rprd1a	-3.93
Zadh2	-3.99
Cd93	-4.02
Melk	-4.07
Rcan2	-4.07

Lpcat1	-4.17
Traj29	-4.17
Traj28	-4.17
Traj27	-4.17
Ensa	-4.28
Cachd1	-4.28
Acpp	-4.35
1700104B16Rik	-4.37
Gtf2e2	-4.37
Sept10	-4.43
Sowahc	-4.43
Sowahc	-4.43
Grm8	-4.45
Wapal	-4.45
Cldn26	-4.50
Zbtb21	-4.53
Gm10742	-4.55
Jmy	-4.63
Prkd1	-4.67
Gm5334	-4.68
Ccdc39	-4.85
Elovl1	-4.94
Bag2	-4.97
Leprot	-5.12
Pdcd1	-5.20
Gm24382	-5.28
4930525G20Rik	-5.32
Calcr	-5.82
Sv2c	-5.96
Gm22313	-6.03
E330034G19Rik	-6.27
Scn1a	-6.39
Gm13629	-6.39
Kdm4a	-6.40
Fbxl3	-6.72
Gm23965	-7.10
Fkbp3	-7.24
Fancm	-7.24
Melk	-9.27
Pon2	-9.70
Ces1d	-9.90
Gm14736	-11.56
Cux1	-12.71
